# Polyoxometalate Functionalized Sensors: A Review

**DOI:** 10.3389/fchem.2022.840657

**Published:** 2022-03-08

**Authors:** Marta I. S. Veríssimo, Dmitry V. Evtuguin, M. Teresa S. R. Gomes

**Affiliations:** ^1^ CESAM, Department of Chemistry, University of Aveiro, Aveiro, Portugal; ^2^ CICECO, Department of Chemistry, University of Aveiro, Aveiro, Portugal

**Keywords:** polyoxometalate (POM), POM hybrid materials, electrochemical sensors, optical sensors, piezoelectric sensors

## Abstract

Polyoxometalates (POMs) are a class of metal oxide complexes with a large structural diversity. Effective control of the final chemical and physical properties of POMs could be provided by fine-tuning chemical modifications, such as the inclusion of other metals or non-metal ions. In addition, the nature and type of the counterion can also impact POM properties, like solubility. Besides, POMs may combine with carbon materials as graphene oxide, reduced graphene oxide or carbon nanotubes to enhance electronic conductivity, with noble metal nanoparticles to increase catalytic and functional sites, be introduced into metal-organic frameworks to increase surface area and expose more active sites, and embedded into conducting polymers. The possibility to design POMs to match properties adequate for specific sensing applications turns them into highly desirable chemicals for sensor sensitive layers. This review intends to provide an overview of POM structures used in sensors (electrochemical, optical, and piezoelectric), highlighting their main functional features. Furthermore, this review aims to summarize the reported applications of POMs in sensors for detecting and determining analytes in different matrices, many of them with biochemical and clinical relevance, along with analytical figures of merit and main virtues and problems of such devices. Special emphasis is given to the stability of POMs sensitive layers, detection limits, selectivity, the pH working range and throughput.

## 1 Introduction

### 1.1 Polyoxometalates

Polyoxometalates (POMs) are negatively charged polyoxoanions of general formula [M_m_O_y_] ^n–^, where M represents the metal centre surrounded by oxygen atoms (O). They are typically composed of transition metal ions in their highest oxidation state (e.g. M = V^V^, Mo^VI^, W^VI^, Ta^V^, Nb^V^), bridged by oxo ligands (O^2–^), to form closed 3-dimensional frameworks (Pope and Müller, 1991). Other elements, mainly heteroatoms, acting as coordination centres, usually labelled as X, can be part of the POM framework [X_x_M_m_O_y_]^n−^ (Pope and Müller, 1991; Hutin et al., 2013). The growing interest in POMs is focused on two main features: 1) the structural diversity due to the coordination flexibility in their metal-oxo structures, and 2) the vast number of elements of the periodic table that can be incorporated inside POM clusters, leading to an overwhelming diversity of molecular structures, of various shapes and sizes, with a diverse range of physical and chemical properties.

### 1.2 Historical Pathway of Polyoxometalate

Briefly, the history of POMs started in 1783, when Scheele (Long et al., 2010) studied reduced molybdenum salts and discovered what are now known to be the first examples of *Molybdenum Blues*, followed by the ammonium phosphomolybdate, a yellow precipitate containing the anion [PMo_12_O_40_]^3−^, discovered by Berzelius in 1826 (Hutin et al., 2013). However, only in 1864, with the discovering of the tungstosilicic acids and their salts, now known as [H_4_SiW_12_O_40_]·xH_2_O, the analytical composition of the 12:1 heteropoly species were precisely determined by Galissard de Marignac (Marignac, 1864). By 1908, around 750 POMs were known. Yet, it was only in the early 30s that J.F. Keggin, using powder X-ray diffraction measurements, revealed the structure of the phosphotungstic acid H_3_ [PW_12_O_40_)].29H_2_O, which carry his name and is known as the Keggin structure (Keggin, 1934).

**FIGURE 1:**
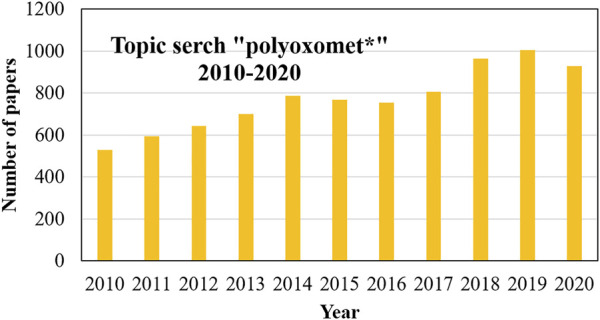
Number of polyoxometalate publications from 2010 to 2020 (Web of Science, August 2021).

In 1991, Pope and Müller (Pope and Müller, 1991) summarised the key features of POMs, highlighting the structural diversity due to the coordination flexibility in their metal-oxo structures and their ability to be functionalized by incorporating virtually any metal from the periodic table. This paper led to an impressively increase in POM’s popularity, reaching an average of 500 publications/year in 2010 (Hutin et al., 2013). Nevertheless, with the advances in material science and nanotechnology, POMs are still seen as promising unique chemical species that could turn very special molecules into very useful materials, as evidenced by the average of around 1,000 articles in each of the last 3 years. Figure 1 shows the evolution in the number of POMs publications (Web of Science, August 2021).

### 1.3 Classification and Structure

In general, POMs are divided into three main classes:

i) Isopolyoxoanions are POMs of the general formula [M_m_O_y_]^n–^containing only one type of high-valent group V or VI transition metal (M) ion, which is called the addenda atom, and oxygen (O). Commonly, those are much more unstable than their heteropolyoxoanions counterparts (Long et al., 2010).
ii) Heteropolyoxoanions are POMs of the general formula [X_x_M_m_O_y_]^n–^, containing a high atomic proportion of one type of transition metal atom (M) and a much smaller proportion of the other types of atoms (X), called heteroatoms. X is usually a *p* or *d* block element, such as B, S, P or Co. The heteroatom can be either primary, whenever it is essential to the POM structure, or secondary, whenever it fills external vacancies of lacunary structures and can be used, for instance, to link the POM structures to form larger aggregates. Lacunary structures are obtained by the selective removal of one or more metal ions that can be occupied. This strategy of occupying the lacunes with other metal or non-metal atoms is commonly used to modify the structure and properties of POMs. A vast number of POMs derivatives with fascinating architectures have been reported over the years through the self-assembly of purely inorganic building blocks and/or the bridging functions of metal ions and organic ligands (e.g., transition metal-inserted POMs, Ln-substituted POMs, heterometallic POMs, and organic ligand modified POMs) (Wang et al., 2020a).
iii) The third class are the Mo-blue and Mo-brown reduced POM clusters, which are easily recognized by their giant nanosized polymolybdates, such as {Mo_154_} “big-wheel” or the {Mo_132_} “big ball” clusters (Hutin et al., 2013).

**FIGURE 2:**
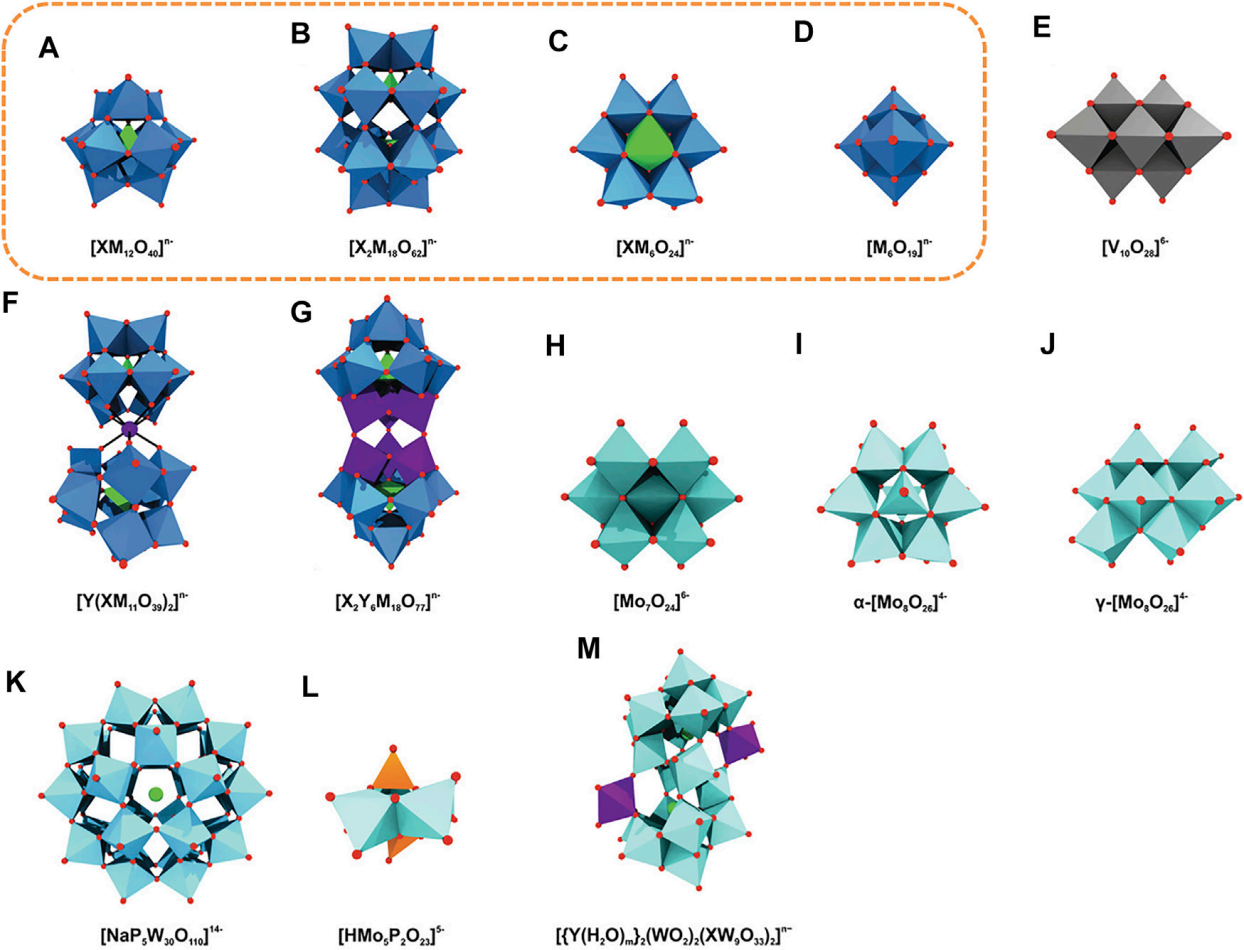
Overview of various archetypal POM anions. **(A)** Keggin, **(B)** Wells–Dawson, **(C)** Anderson, **(D)** Lindqvist, **(E)** decavanadate, **(F)** sandwich Keggin, **(G)** double Keggin, **(H)** heptamolybdate, **(I)** *α*- and **(J)** *γ*-octamolybdate, **(K)** Preyssler, **(L)** Strandberg, and **(M)** Krebs-type structure. Blue polyhedra are {MO_6_} (M = any addenda atom), light green polyhedra {XO_*n*_} (X = heteroatom), light green spheres sodium, light blue polyhedra {WO_6_}, light cyan polyhedra {MoO_6_}, gray polyhedra {VO_6_}, purple polyhedra and spheres {YO_*n*_} and Y (Y = second heteroatom), orange polyhedra {PO_4_}, red spheres oxygen. The most common polyoxometalates archetypes are highlighted with a yellow box. Adapted from Bijelic et al. (2019).

POMs are seen as the largest non-biologically derived molecules structurally characterized, and these building blocks can be used to construct the systems represented as observed in Figure 2 [adapted from Bijelic et al. (2019)]. Although POMs exhibit a vast diversity in size and structure, the majority of POMs can be identified as one of the four distinct structural families: Lindqvist, Anderson, Keggin, and Well-Dawson type clusters, highlighted in Figure 2 with a yellow box. Such structures are dominant in the field because of their high reproducibility and can be formed by several different types of addenda metal atoms. The Lindqvist structure is the smallest of the four POM types and is adopted by isopolyoxometalates of formula [M_6_O_19_]^n–^. It consists of an octahedral arrangement of six octahedra. Each octahedron, consisting of a metal ion with its coordination sphere, shares four edges with four neighbouring octahedra. The Keggin structure, the most popular structure for heteropolyoxometalates, has a general formula of [XM_12_O_40_]^n–^, or XM_12_, with tetrahedrally coordinated heteroatoms and four trimetallic groups arranged around a central tetrahedron. The Anderson-Evans structure, also commonly designated as Anderson structure, is the smallest of the common heteropolyoxoanions, incorporating a single heteroatom, X, with the formula [XM_6_O_24_]^n–^, or XM_6_, with six edge-sharing octahedra arranged into a planar hexagon around the central heteroatom, X. The Well-Dawson structure, commonly known as Dawson structure, is a heteropolyoxometalate with the general formula [X_2_M_18_O_62_]^n–^, or X_2_M_18._ The structure can be seen as a connection of two Keggin units, each of them lacking a {M_3_O_13_} unit and connected by a shared corner.

### 1.4 POM Synthesis, Hybrid Materials and Immobilization

#### 1.4.1 Synthesis

The synthesis of POMs is easy to carry out. Basically, all that is needed is an acidic solution containing the relevant metal oxide anions. The reaction either takes place in a single step and is said to be a “one-pot” synthesis or in a small number of multiple steps, using common and relatively inexpensive reagents. Still, a few parameters should be considered in POMs synthesis, namely, the concentration/type of metal oxide anion, pH, ionic strength, heteroatom type/concentration, presence of additional ligands, the reducing agent, the temperature of the reaction, and the process (e.g., microwave, hydrothermal, refluxing) (Long et al., 2010).

POM properties, including molecular composition, size, shape, charge density and redox potentials, are easily tailored by defining the synthesis parameters. Besides, POMs can be rendered soluble in nearly any media, from H_2_O to hydrocarbons, by properly choosing counter-cations.

#### 1.4.2 POM-Based Composite Materials

One remarkable feature of POMs is their ability to be functionalized by incorporating practically any metal ion from the periodic table. Not many inorganic materials achieve such chemical and structural diversity. Although using pristine POMs can have several disadvantages for specific applications, such as poor conductivity, low specific surface area, leaching, degradation, aggregation, and solubility in aqueous solutions, their functionalizing flexibility can be used to improve the materials. It is possible to obtain highly redox-active materials able to undergo complex electron transfer, making POMs highly desirable functional materials for a myriad of applications.

A very effective way to obtain POMs with specific properties is to form hybrid composite materials by loading POMs onto different supports. The most interesting POM-based composite materials recently reviewed (Miras et al., 2014; Ji et al., 2015a; Herrmann et al., 2015; Wang et al., 2020a; Khalilpour et al., 2021) include POMs-nanocarbon composites that enhance electronic conductivity, POMs-metal composites that increase catalytic and functional sites, POMs-conductive polymers composites that increase conductivity and develop flexible and easily processable materials, and POMs-metal-organic frameworks (POMOFs) composites that increase surface area, expose more active sites and improve stability. Lately, the synergetic effect observed on POM-based multi-material composites (usually triple-materials) has gained increased attention. The combination of the respective advantages of different materials can sometimes endow the composite with unexpectedly improved properties where each material works synergistically, giving superior performances.

#### 1.4.3 Immobilization Procedures

POMs are often anchored or immobilized onto substrates. POMs can be attached to substrate surfaces by covalent, electrostatic, or supramolecular bonds and can be present in 3D matrices, on nanostructures, or on flat surfaces. The five main strategies for attaching POM or POM-based materials to a substrate (Cherevan et al., 2020) are dip-coating, Layer-by-Layer (LbL) process, electrochemical deposition, solvothermal deposition and drop-casting, and are briefly described below.

i) Dip-coating is the most simple, easy, and straightforward method, where the substrate is immersed in a solution containing the POM. It has the disadvantages of being prone to leaching, non-homogeneous distribution on the substrate surface, and lack of reproducibility.
ii) The Layer by layer (LbL) process consists of alternate adsorption of opposite charges layers, held in place by electrostatic and Van der Walls forces. This process is known for its simplicity, thickness controlled by adjusting the number of deposited layers, high stability and mechanical strength, and very uniform morphology. Though, depending on the intended thickness, it could be time-consuming.
iii) Electrochemical deposition is limited to conductive substrates and is performed under controlled potential or current, with POMs being deposited on the anodes due to their negative charge, forming monolayers or multilayers. In addition, it enables the obtention of direct electrochemical information about the deposition process.
iv) The Solvothermal deposition occurs in a closed system and requires high temperature and pressure to anchor POM on the substrates. It has the advantage of enabling highly condensed, insoluble lattices while preventing or decreasing leaching and avoiding reversible deposition. However, it is impossible to observe the reaction process (“black box”), and the harsh conditions could lead to structural re-arrangements of POMs.
v) Drop-casting is an easy and fast immobilization method, where POMs are dispersed in a suitable solvent which is then dropped onto a flat surface, followed by evaporation of the solution, forming a thin solid film. This technique is frequently used to modify electrode surfaces for electrocatalysis. Unfortunately, it is not easy to get a uniform coating with a controlled thickness.

### 1.5 POMs Applications: Overview

The unique versatility in size, thermal stability, multiple and fast redox reactions, photochemical response redox, and magnetic properties are some of the physical and chemical properties that make POMs promising candidates for a wide range of applications. By far, the most popular application of POM-hybrid materials is as catalysts due to their super acidity and excellent structural stability undergoing multi-electron redox cycles (Katsoulis, 1998; Sadakane and Steckhan, 1998; Ren et al., 2015; Patel et al., 2016).

POM anions are also versatile inorganic building blocks for the construction of solid functional materials (Miras et al., 2012). Due to the unique properties of POM hybrid systems, combining redox-active POMs as electron storage sites with nanostructured carbon conducting materials with a high surface, they find applications in the energy field, such as energy storage, energy conversion, and fuel cells (Chen et al., 2015a; Ji et al., 2015a; Herrmann et al., 2015).

POMs also exhibit many ideal properties for use in biological and medical disciplines. They could be extremely small (sub 5 nm), therefore liable to be cleared by the renal system, exhibit low toxicity and are stable in biological media. A great variety of roles have been reported to POMs in combination with natural polymer molecules (proteins, peptides, and amino acids) by taking advantage of the different characteristics of both moieties (Arefian et al., 2017; Bijelic and Rompel, 2018). Additionally, POMs have been gaining relevance in medicine due to their application as antiviral, antibacterial, and antitumor agents and as radiosensitizers in cancer therapy (Bijelic et al., 2019; Guedes et al., 2020; Aureliano et al., 2021).

POMs are also ideal for substrate sensing, and this review aims to present and discuss functionalized POM sensors, explore their applications, and assess their feasibility and contribution to the sensors field.

## 2 Polyoxometalates Functionalized Sensors

**FIGURE 3:**
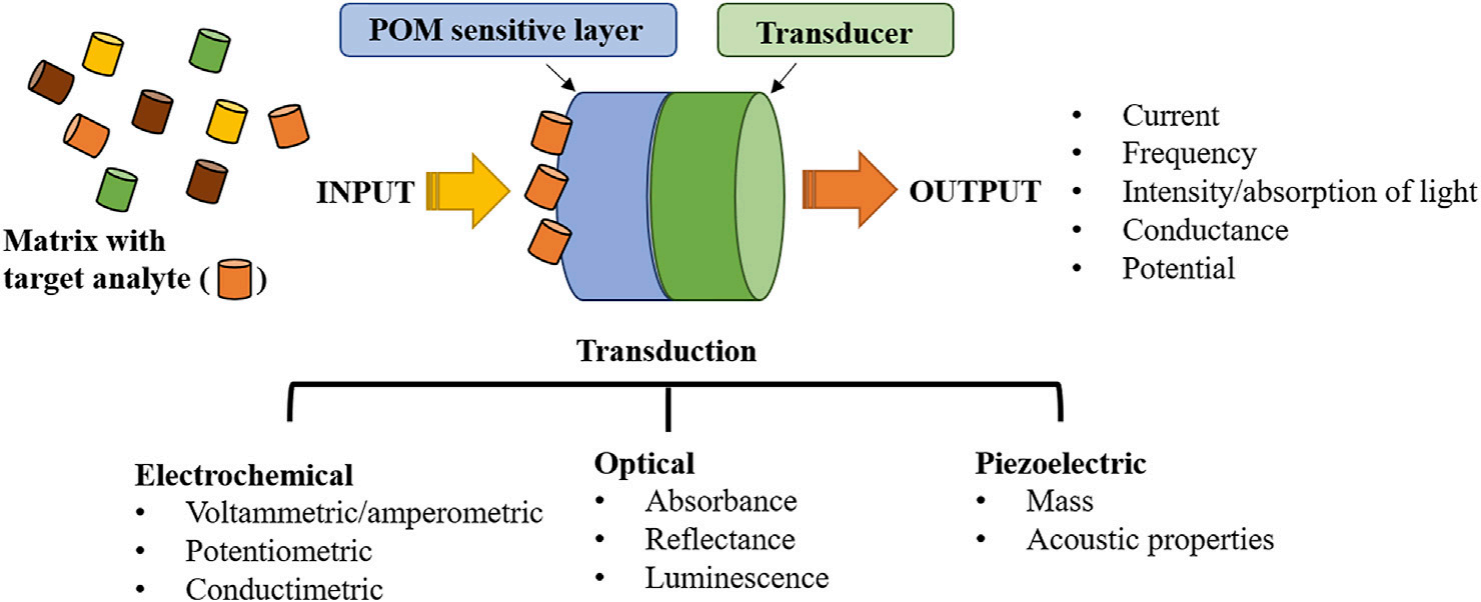
Overview of the sensor composition: from polyoxometalate analyte recognition layer to the transducer and measured signal.

A POM-based sensor can be defined as an analytical device comprising an immobilized layer of POM on a transducer (Ammam, 2013), as depicted in Figure 3. While POM will be responsible for sensor sensitivity and selectivity, the transducer will be an electrical device responsible for converting one form of energy into another, handling different types of energies such as mechanical, electrical, light, chemical, thermal, acoustic, electromagnetic, etc. After POM immobilization, it will recognize and interact with the analyte. The physical and chemical changes induced by the analyte onto the immobilized POM will be then transformed into an electrical signal, amplified, and converted by the signal processing equipment into a readout signal (Ammam, 2013) (schematically presented in Figure 3). In general, sensitivity depends on the success in POM immobilization and on the deposited POM activity towards a specific analyte (Veríssimo et al., 2010; Gamelas et al., 2018). Selectivity deals with the POM’s preference towards the analyte regarding other species in the sample matrix. The limit of detection (LOD) is the smallest quantity that can be reliably detected. Despite the existence of several quantitative definitions, it is based on the detection of a signal over noise (usually a concentration corresponding to a signal 3 times the noise is accepted) (Veríssimo et al., 2020a). The linear working range of a sensor is the range of concentrations going from the lowest concentrations that can be reliably quantified, the quantification limit (LOQ), and the concentration at which the signal dependence to concentration is no longer linear. Stability deals with the degree to which sensor characteristics remain constant over time. The final goal of any sensor is to reliable detect or quantify an analyte in real samples and, therefore, adequate sensitivity must be assured (Veríssimo et al., 2020b). Sensors are an indispensable tool for our lives, whether detecting a highly toxic metal in lake waters, detecting food frauds, and assuring its safety, or providing clinical tests and detecting cancer at early stages. They can provide security, save lives and improve quality of life.

**FIGURE 4:**
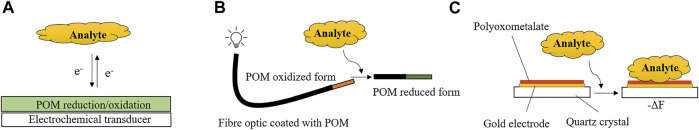
Schematic overview of the most common operation principle of a POM-based electrochemical sensor **(A)**, POM-based fibre optic sensor **(B)** and POM-based mass sensor **(C)**.

POM-based sensors will be divided and discussed according to their transducer principle: electrochemical, optical and piezoelectric (mass). Figure 4 schematically shows one possible arrangement of a POM-based device for each transducer.

### 2.1 POM-Based Electrochemical Sensors

Electrochemical sensors extract information about the analyte from the measurement of some electrical parameters. They can be categorized according to the measured electrical parameter: potential (potentiometric sensors), current (amperometric sensors), and resistance or conductance (conductimetric sensors, namely chemiresistors and semiconductor metal oxide sensors).

#### 2.1.1 POM-Based Conductimetric Sensors

Albeit conductometric/chemiresistive sensors have the advantage of low-cost fabrication, only a few articles were found reporting chemiresistors or conductimetric POM-based sensors. A resistive humidity sensor based on a Keggin H_3_PMo_12_O_40_- polypyrrole nanocomposite was reported by Miao et al. (2018). To prepare the humidity sensing material by co-electrodeposition, the polyoxoanion of the phosphomolybdic acid was chosen as the anionic building block and the protonated polypyrrole (PPy) was selected as the cationic building block because of its relatively large size and hydrophobicity. The optimized resistive humidity sensor showed a rapid response and recovery time (1.9/1.1 s at a 98% RH level, respectively), a sensing range of 11–98% RH, excellent durability, and repeatability with little hysteresis, superior to commercial thermosetting polyester. Other POMs have also been used to enhance gas sensing, such as the Keggin H_5_PMo_10_V_2_O_40_ that was proposed to mediate the key Pt (II)-Pt (IV) oxidation while itself being regenerated by O_2_, for detection of methane in chemiresistive sensors (Bezdek et al., 2021), the cyanometalate-functionalized POM (C_4_H_10_ON)_23_ [HN(CH_2_CH_2_OH)_3_]_10_H_2_ [Fe^III^(CN)_6_ (*α*_2_-P_2_W_17_O_61_Co^II^)_4_]·27H_2_O to improve photoconductivity and gas sensing performances for formaldehyde and methylbenzene in SnO_2_-based gas sensors (Wang et al., 2017), and the Keggin H_4_SiW_12_O_40,_ used as a dopant for detecting a series of chemical vapours in polyaniline (PANI) nanotubes (Gao et al., 2007). Also, Amman *et al.* (Ammam and Easton, 2011a) reported a POM hybrid compound, the [K_4_(Py)_2_(P_2_Mo_18_O_62_)], for detection of NOx, a generic term for nitric oxide NO and nitrogen dioxide (NO_2_), both toxic gases that can be harmful to health in various ways. Starting with a Dawson K_6_P_2_Mo_18_O_62_. nH_2_O, which was used as an oxidizing agent to polymerize pyrrole (Py) in the presence of the mild reducing agent potassium iodide (KI), they accomplished to generate a hybrid material with low Py content. The resulting semiconducting composite illustrated a selective and sensitive response to NOx gases when exposed to various gases and extended linearity up to 5,500 ppm.

#### 2.1.2 POM-Based Amperometric Sensors

It must be emphasized that most POM-based electrochemical sensors found in the literature are amperometric. The standard electrochemical cell consists of a working electrode, a counter electrode, and a reference electrode connected to a potentiostat that controls the working electrode potential and measures the current. The working electrode is the one on which the reaction of interest occurs, the oxidation or reduction of species. Therefore, the correct choice of the working electrode is vital for a successful application. Cost, electrical conductivity, chemical stability, activity towards the analyte, and wide potential range are the most important prerequisites that should be considered in choosing the electrode.

The high redox activity of POMs makes them ideal for the electrocatalytic transfer of electrons to or from a substrate while retaining their structural integrity. However, the application of ordinary POMs in chemically modified electrodes (CMEs) is not straightforward. Ordinary POMs-based CMEs present low stability due to POM’s high solubility in aqueous solutions. Practical applications of POMs for the preparation of CMEs depend on the successful immobilization of these compounds. The solution lies in fabricating organic-inorganic hybrid materials, allying the insolubility of organic compounds with the excellent catalytic properties of inorganic POMs.

##### 2.1.2.1 Sensing of Hydrogen Peroxide

Hydrogen peroxide (H_2_O_2_) is an oxidant widely used in food and pharmaceutical proceedings as a sterilizing agent and released from several industrial processes. In addition, H_2_O_2_ is the product of the reactions catalysed by many oxidases, which is fundamental in food, pharmaceuticals, and environmental analysis. H_2_O_2_ can be detected electrochemically, and its concentration can be readily monitored and used to measure the amount of a particular biological species (Ammam and Easton, 2012). To date, a significant number of enzyme-based electrochemical sensors have been developed to detect H_2_O_2_. However, the enzymatic biosensors are limited by the poor stability, high cost, complicated immobilization procedure, and critical operational conditions inherent to the nature of enzymes. Nevertheless, enzyme-free electrochemical sensors for H_2_O_2_ have gained special attention and have become a current trend. POM-based electrochemical sensors for H_2_O_2_ have been reported as appealing non-enzymatic alternatives due to their fast response and high sensitivities, achieved by exploring the synergetic effect of hybrid materials enhancing electrocatalytic activity and stability. Table 1 summarizes the electrochemical sensors for H_2_O_2_ found in the literature for the last 2 decades, using electrodes modified with POMs. Table 1 provides information about the POM-hybrid material used to modify the electrode (POM-hybrid@electrode), the archetypal of POM anions, the working pH, the limit of detection, the stability of the sensor and if the sensor was applied to real samples (more details can be found in Supplementary Table S1 in Supplementary Material). In Table 1, notice the work of Guo et al. (2015a), who reported a good example of a POM-modified electrode for H_2_O_2_ consisting of a nanocomposite film containing the Dawson K_6_P_2_W_18_O_62_ (P_2_W_18_), carbon nanotubes (CNTs), and Au nanoparticles (AuNPs), abbreviated as P_2_W_18_/CNTs/AuNPs, applied on an indium tin oxide (ITO) electrode (listed in Table 1 as P_2_W_18_/CNTs/AuNPs@ITO). The sensor showed a good linear range (1–98 mM), an excellent detection limit (52 nM), and a response time to H_2_O_2_ of less than 1s. Another good example of a non-enzymatic H_2_O_2_ sensor was reported by Berbéc *et al.* (Berbeć et al., 2018), and this one was based on a Keggin PMo_12_/AuNPs/reduced graphene oxide (rGO) over a glassy carbon electrode (GCE) and can be found in Table 1 under the designation of PMo_12_/AuNPs/rGO@GCE. Comparing the figures of merit of H_2_O_2_ analytical determination in Supplementary Table S1 with the two electrodes, it could be concluded that linear range and detection limits were similar, but in what concerns to sensitivity (listed in Supplementary Table S1, the Berbéc electrode was superior (sensitivity of 596.1 μA mM^−1^ cm^−2^ and 740.8 μA mM^−1^ cm^−2^, for Guo (Guo et al., 2015a) and Berbéc (Berbeć et al., 2018) electrodes, respectively) due to the rGO layer on the AuNPs/GC electrode, which significantly improved the sensitivity of AuNPs to H_2_O_2_. Nevertheless, both modified electrodes showed that the combination of carbon materials and AuNPs was responsible for significant enhancement of the sensor’s performance. Despite those figures of merit, those electrodes have a limitation related to the usual POMs instability at neutral pH, which constitutes a major drawback as they are restricted to work in acidic media, and therefore cannot be used in physiological systems.

**TABLE 1:** POM-based electrochemical sensors for H_2_O_2_ detection.

Hybrid material@Electrode	POM archetype	Matrix	pH	Limit of detection	Stability studies	References
P_2_W_17_V/graphite/organoceramic@CPE	b	no	acidic	4 × 10^−5^ M	3 months	Wang et al. (2000)
PMo_12_@Pt	a	no	acidic	7 × 10^−6^M	NR	Song et al. (2000)
Fe_4_POM^a^/poly (1,8 DAN)@GE	a	no	2.5	2 mM	no	Turdean et al. (2002)
(H_6/5_bppy)_5-_P_2_W_18_@CPE	b	no	acidic	1.3 × 10^−5^ M	1 month	Tian et al. (2007)
P_2_Mo_18_/OMC@GCE	b	no	acidic	53.4 μΜ	NR	Zhou et al. (2007)
SWCNTs/SiMo_12_/[Cu(bpy_)2_]^2+^@GCE	a	no	1	1 nM	30 days	Salimi et al. (2009)
APS/PFeW_11_@CPE	a	no	2	7.4 μΜ	NR	Hamidi et al. (2009)
VMo_12_/[BMIM][PF_6_]@CPE	a	no	4	2.33 μΜ	2 weeks	Ji et al. (2009)
MWCNTs/[C_8_Py][PF_6_]/PMo_12_@GCE	a	no	1	12 μΜ	20 times a day/5 days	Haghighi et al. (2010)
MPS/B/PFe_3_Mo_9_@Au	a	no	6.2	NR	NR	Turdean and Popescu, (2012)
K_5_ [Ru (bpy)_3_]-PW_18_@GCE	b	no	7	0.5 μΜ	5 weeks	Ammam and Easton, (2012)
P_2_W_17_Fe/PdNPs@ITO	b	no	2	1 μΜ	1 month	Zhu et al. (2013)
P_8_W_48_/chitosan/PEI@ITO	n	no	5	1.3 μΜ	2 months	Kang et al. (2013)
AuNPs/PW_12_/OMC@GCE	a	disinfectant solution	7	0.36 μΜ	2 weeks	Zhang et al. (2015)
P_2_W_18_/CNTs/AuNPs@ITO	b	no	2	52 nM	20 days	Guo et al. (2015a)
PW_12_/PEI@ITO	a	no	5	8.4 × 10^−4^ mg/ml	NR	Xu et al. (2015)
PtNPs/PMo_12_/OMC@GCE	a	no	7	1.9 μΜ	2 weeks	Li et al. (2016)
PMo_12_/PANI@Au	a	no	acidic	8.1 μΜ	NR	Yang et al. (2016b)
PMo_12/_rGO@GCE	a	no	acidic	10.2 μΜ	NR	Yang et al. (2016a)
PMo_12_/PEI@ITO	a	no	5	0.2 μg ml^−1^	100 cycles	Hao et al. (2017)
NENU5^b^-KB@GCE	a	no	7.4	1.03 μΜ	4 h	Wang et al. (2018a)
PMo_12_/AuNPs/rGO@GCE	a	no	6	56 nM	NR	Berbeć et al. (2018)
Ag_4_L^a^_5_SiW_12_@CPE	a	no	acidic	5.54 × 10^−6^ M	NR	Tian et al. (2018a)
Ag_3_L^a^_4_PW_12_@CPE	a	no	acidic	1.28 × 10^−6^ M	NR	Tian et al. (2018a)
Ag_6_L^b^_6_PMo_12_@CPE	a	no	acidic	4.95 × 10^−6^ M	NR	Tian et al. (2018a)
Ag_4_L^a^_2_L^b^_4_GeMo_12_@CPE	a	no	acidic	5.45 × 10^−6^ M	NR	Tian et al. (2018a)
Cu_2_(H_2_bdpm)_2_P_2_W_18_@CPE	b	no	acidic	1.4 × 10^−5^ M	NR	Tian et al. (2018b)
(Cu_3_(pdp)_6_Cl_2_)PCuMo_11_@CPE	a	no	acidic	1.7 × 10^−5^ M	NR	Tian et al. (2018b)
PEI/rGO/AuNPs/P_8_W_48_@ITO	n	no	7	0.31 μΜ	NR	Zhang et al. (2019a)
MWCNTs/[C_12_Py][PF_6_]/PMo_12_@GCE	a	no	1	241 μΜ	100 cycles day/5 days	Feizy and Haghighi, (2019)
{K(H_2_O)}_2_{Cu_2_(bim)_2_}_2_P_2_W_18_@GCE	b	no	acidic	72.1 mM	1 month	Gao et al. (2020a)
[Ag (bpy)][{Ag(Hbpy)}_2_AlW_12_@GCE	a	no	acidic	0.93 μΜ	1,000 cycles	Gong et al. (2020)
[H_2_en][{Cu(bpy)}_3_AlW_12_@GCE	a	no	acidic	0.86 μΜ	1,000 cycles	Gong et al. (2020)
[Mo-oxo]_n_/N-MPC@GCRDE	a	no	7	0.23 μΜ	2 months	Liu et al. (2020a)
Ag-Fe_2_O_3_/PMo_12_/rGO@GCE	a	local river	6.8	0.2 μΜ	NR	Ross and Nqakala, (2020)
(Ag_7_bpy_7_Cl_2_)AsW_12_@GCE	a	human serum	7.4	0.48 μΜ	3 days	Cui et al. (2020)
{P_5_W_30_}/Mn/H_2_bimb@GCE	k	no	7	0.44 mM	No	Zhu et al. (2021)
{P_5_W_30_}/Co./H_2_bimb@GCE	k	no	7	0.13 mM	10 h	Zhu et al. (2021)
{P_5_W_30_}/Cu/H_2_bimb@GCE	k	no	7	0.47 mM	no	Zhu et al. (2021)
{P_5_W_30_}/Zn/H_2_bimb@GCE	k	no	7	0.62 mM	no	Zhu et al. (2021)
[Cu(MET)_2_]Mo_8_@CPE	j	no	acidic	6.65 × 10^−5^ M	NR	Zhang et al. (2021a)
[Cu(bpy)]Mo_2_@CPE	—	no	acidic	8.9 × 10^−4^ M	NR	Zhang et al. (2021a)
Cu_3_(OH)_4_(Ptla)_2_TeMo_6_@CPE	c	no	acidic	9.77 × 10^−4^ M	NR	Ying et al. (2021)
Cu_2_(OH) (Ptep)_2_Mo_8_@CPE	—	no	acidic	4.52 × 10^−3^ M	NR	Ying et al. (2021)

Abbreviations as reported by authors. [BMIM][PF_6_], 1-butyl-3-methylimidazolium hexafluorophosphate; [C_8_Py][PF_6_], n-octylpyridinium hexafluorophosphate; APS, 3-aminopropyl(triethoxy)silane; Au, gold; AuNPs, Au nanoparticles; B, ethylamine; bim, biimidazole; bimb, 1,4-bis(1H-imidazol-1-yl)benzene; bppy, 4-(5-(4-bromophenyl)pyridin-2-yl-)pyridine); bpy, 4,40-bipyridyl; CPE, carbon paste electrode; en, ethylenediamine; GCE, glassy carbon electrode; GCRDE, glassy carbon rotating disk electrode; GE, graphite electrode; H_2_bdpm, 1,1′-bis(3,5-dimethyl-1H-pyrazolate)methane; ITO, indium tin oxide electrode; KB, ketjenblack; L^a^, 2,3-diphenylpyrazine; L^b^, 2,3-diphenylquinoxaline; MET, 4-(3-imidazol-1-yl-ethyl)-4H-[1,3,4]triazole; MPS, 3-mercapto-1-propanesulfonic acid; MWCNTs, multi walled carbon nanotubes; N-MPC, nitrogen-doped mesoporous carbon; NR, not reported; OMC, ordered mesoporous carbon; PANI, polyaniline; PdNPs, Pd nanoparticles; pdp, 4-propyl-4, 5-dihydro-1H-pyrazole; PEI, Poly(ethyleneimine); Pt, platinum; Ptep, 1-{2-[3-pyridin-4-yl-(1,2,4)triazol-4-yl]-ethyl}-piperazin; Ptla, 2-[3-pyridin-4-yl-(1,2,4)triazol-4-yl]-ethylamine; rGO, reduced graphene oxide; SWCNTs, single walled carbon nanotubes.

a Na_6_ [H_4_Fe_4_(PMo_9_O_34_)_2_(H_2_O)_2_]H_2_O.

b [Cu_2_(BTC)_4/3_(H_2_O)_2_]_6_ [H_3_PMo_12_O_40_].

POM archetype according to the legend of Figure 2: a) Keggin, b) Dawson, c) Anderson, j) *γ*-octamolybdate, also including n) crown-shape, k) Preyssler and -) unspecified type.

Salimi et al. (2009) reported another type of POM-based hybrid composite that showed not only better performance but extended the pH working range a bit further. The new material was a combination of the Keggin SiMO_12_, single-walled carbon nanotubes (SWCNTs), and a cationic copper complex resulted in a three-component hybrid composite SWCNTs/SiMo_12_/[Cu(bpy)_2_]^2+^. Due to electrostatic attraction between anions and cations, the stability of adsorbed heteropolyanion and Cu-complex increased at somewhat higher pH values (pH < 7). Even so, authors performed the amperometric detection of H_2_O_2_ at pH 1, where the system (as represented in Table 1 SWCNTs/SiMo_12_/[Cu(bpy)_2_]^2+^@GCE) was able to detect nanomolar concentrations (1 nM), with values comparable or even better than other electrodes modified with Cu^2+−^complexes reported in the literature (Salimi et al., 2009). In addition, this system showed a linear range of 10 nM–18 mM, stability for 30 days, and proved to be successful towards bromate reduction (reported ahead in Table 3). Although less sensible (at μM level), other authors (Ammam and Easton, 2012; Zhang et al., 2015; Li et al., 2016; Zhang et al., 2019a; Liu et al., 2020a; Zhu et al., 2021; Cui et al., 2020; Wang et al., 2018a) achieved to improve electrochemical properties and stability at high pH (physiological level) by pairing POM anions (Keggin, Dawson, crown-shape and Preyssler) with organic or organometallic compounds (Ammam and Easton, 2012; Zhu et al., 2021), with carbon materials decorated with metal nanoparticles (Zhang et al., 2015; Li et al., 2016; Zhang et al., 2019a) or nitrogen (Liu et al., 2020a), and with metal-organic frameworks (MOFs) (Cui et al., 2020; Wang et al., 2018a), that enhanced POM stability at less acidic pH. It is worth mentioning that for the application of POM complexes in an aqueous solution, a thorough insight into the solution chemistry is essential in order to understand the reaction mechanism. The recent work from Gumerova and Rompel (Gumerova and Rompel, 2020) summarizes the species that are present in isopoly- and heteropolyvanadates, -niobates, -molybdates and -tungstates aqueous solutions and covers their stability and transformations, presenting ion distribution diagrams over a wide pH range. These diagrams showed the POM species that are in equilibrium at the given pH value and could help researchers to design the POM according to desired target. Nevertheless, among the sensors for H_2_O_2_ represented in Table 1, working at physiological levels (between pH 7.0 and 7.4), the one exhibiting the best performance was a modified glassy carbon rotating disk electrode (GCRDE) with a POM combined with a Nitrogen-doped mesoporous carbon (N-MPC) (Liu et al., 2020a). This [Mo-oxo]_n_/N-MPC@GCRDE (as listed in Table 1) modified electrode showed a good sensitivity of 2.2 mA mM^−1^ cm^−2^, a detection limit of 0.23 μM, a wide linear range from 50 μM to 5 mM, a response time of 2s, and excellent stability along 2 months. In addition, it was unaffected by many common contaminants. Besides, it is worth noting that among these sensors for H_2_O_2_ operating at physiological levels, the sensor based on K_5_ [Ru (bpy)_3_]-PW_18_@GCE, a combination of a Dawson type POM and an organometallic moiety, allowed the determination of H_2_O_2_ either by reduction or oxidation, displaying an attractive and rare bifunctional catalytic property.

##### 2.1.2.2 Sensing of Nitrite

Nitrite is commonly used as a food preservative and a fertilizing agent. It is widely present in the soil, water, food, and physiological systems and plays a role in the global nitrogen cycle. However, it is highly toxic because it interacts with amines to form carcinogenic nitrosamines in the stomach, prone to cause gastric cancer. Excessive nitrite in food products, including vegetables, drinking water, and beverages, is a severe threat to human health and has become a global issue, with the European Community stipulating guideline limits of 0.1 mg L^−1^ (∼2.2 mM) for drinking water (The European Parliament and the Council of the European Union, 2020). Analytical techniques used to detect nitrites were often spectrophotometry or spectrofluorimetry, and sometimes a chromatographic or electrophoresis separation is necessary. These techniques require somewhat bulky instrumentation, tedious and time-consuming sample pre-treatment, expensive reagents, or organic and toxic solvents. POM-based electrochemical sensors have been successfully used for nitrite detection due to their low cost, easy operation, and high sensitivity. Table 2 summarizes the POMs modified electrodes for NO_2_^−^ found in the literature. Table 2 reveals detailed information about the POM-hybrid material used to modify the electrode (POM-hybrid@electrode), the POM archetype, the working pH, the limit of detection, the sensor lifetime, and the matrix where the sensor was tested (more details can be found in Supplementary Table S2 in Supplementary Material). It is noteworthy that the best performance was reported by Zuo et al. (2016), using an electrochemical sensor for nitrite based on a Keggin-type POM, H_6_ [PMo_9_V_3_O_40_] (PMo_9_V_3_), a poly (3,4-ethylenedioxythiophene) (PEDOT) and gold nanoparticles (AuNPs) fabricated by a combination of electrodeposition with self-assembly approach. Due to the synergistic contributions of POMs and PEDOT/AuNPs, the composite film electrode exhibited increased electrocatalytic activity towards the oxidation of nitrite and a faster transfer rate than the single-component film. The PEI/PMo_9_V_3_/PEDOT/AuNPs@GCE sensor (as listed in Table 2) showed a wide linear range (2.5 × 10^−9^–1.43 × 10^−3^ M) and a low detection limit of 1 nM, which are much better than most of the reported nitrite sensors (Zuo et al., 2016). Besides, the sensor presented a fast response time of 0.6 s and very good stability for long-term applications. Acceptable recoveries were obtained in a variety of samples (tap water, mineral water, apple juice, milk, and yoghurt) when spiking them with nitrite standards. Although many of the reported sensors were not tested in real samples, most of the nitrite Keggin or Dawson type POM-based sensors in contact with standards presented good stability and showed sensitivity and selectivity adequate to quantify nitrite at the established guideline values for drinking water.

**TABLE 2:** POM-based electrochemical sensors for NO_2_^−^ detection.

Hybrid material@Electrode	POM archetype	Matrix	pH	Limit of detection	Stability studies	References
P_2_Mo_18_/OMC@GCE	b	no	acidic	1.78 μM	NR	Zhou et al. (2007)
PMo_12_/BC@PE	a	no	acidic	1.0 × 10^−4^ M	100 cycles	Liang et al. (2009)
RuSiW_10_/PEI@ITO	a	no	acidic	0.1 mM	NR	Ma et al. (2010)
P_2_W_18_/PVA@ITO	b	no	acidic	0.96 μM	100 cycles/2 months	Cao et al. (2012)
PEI/PSS/PDDA/P_2_W_17_V/CNTs@ITO	b	juices, milk, sausage, pickled vegetable	7.0	0.0367 μM	150 cycles/50 days	Zhang et al. (2013)
SiMo_12_/rGO@ITO	a	tap water	acidic	7.73 μM	100 cycles	Guo et al. (2014)
PPD/SiW_11_@BDDE	a	river water	acidic	20 μM	no	Sahraoui et al. (2015)
PMo_11_/ox-SWCNTs@GCE	a	no	1	3.0 × 10^−5^ M	1 month	Boussema et al. (2016)
PEI/PMo_9_V_3_/PEDOT/AuNPs@GCE	a	tap and mineral water, apple juice, milk, yoghurt	5.1	1 nM	100 cycles/20 days	Zuo et al. (2016)
Cu_2_(H_2_bdpm)_2_P_2_W_18_@CPE	b	no	acidic	4.9 × 10^−5^ M	NR	Tian et al. (2018b)
(Cu_3_(pdp)_6_Cl_2_)PCuMo_11_@CPE	a	no	acidic	8.7 × 10^−5^ M	NR	Tian et al. (2018b)
Ag_4_L^a^_5_SiW_12_@CPE	a	no	acidic	9.22 × 10^−5^ M	NR	Tian et al. (2018a)
Ag_3_L^a^_4_PW_12_@CPE	a	no	acidic	3.19 × 10^−5^ M	NR	Tian et al. (2018a)
Ag_6_L^b^_6_PMo_12_@CPE	a	no	acidic	7.55 × 10^−6^ M	NR	Tian et al. (2018a)
Ag_4_L^a^_2_L^b^_4_GeMo_12_@CPE	a	no	acidic	8.74 × 10^−6^ M	NR	Tian et al. (2018a)
P_2_W_18_/Zn/dbt@CPE	b	no	acidic	2.6 × 10^−5^ M	NR	Ying et al. (2019)
PW_12_/Cd/dbt@CPE	a	no	acidic	3.3 × 10^−5^ M	NR	Ying et al. (2019)
SiW_12_/Cd/dbt@CPE	a	no	acidic	2.2 × 10^−5^ M	NR	Ying et al. (2019)
MWCNTs/[C_12_Py][PF_6_]/PMo_12_@GCE	a	no	1	57 μM	100 cycles day/5 days	Feizy and Haghighi, (2019)
SWNTs/ILC_12_/PMo_12_@GCE	a	no	acidic	1.3 μM	100 cycles	Wang et al. (2019b)
SWNTs/ILC_8_/PMo_12_@GCE	a	no	acidic	1.3 μM	100 cycles	Wang et al. (2019b)
SWNTs/ILC_4_/PMo_12_@GCE	a	no	acidic	1.3 μM	100 cycles	Wang et al. (2019b)
PMo_12_/MoS_2/_rGO@GCE	a	lake water	acidic	0.2 μM	1 month	Xu, (2019)
rGO/PANI/As_2_Mo_2_@GCE	—	beverages, cucumber extract, water	4	10.71 μM	2 months	Suma et al. (2019)
Zn_2_ (bte)_4_SiMo_12_@CPE	a	no	acidic	6.1 × 10^−3^ M	NR	Mou et al. (2019)
Cu^II^_4_(btmc) (ctcm)_4_Mo_8_at CPE	—	no	acidic	1.4 × 10^−7^ M	NR	Wang et al. (2020b)
Cu^II^_4_(mct)_2_ (ctcm)_2_(H_2_O)_6_Mo_8_@CPE	—	no	acidic	5.6 × 10^−7^ M	NR	Wang et al. (2020b)
Cu^II^(dm_4_bt)Mo_3_@CPE	—	no	acidic	1.135 × 10^−7^ M	NR	Wang et al. (2020b)
Co^II^(dm_4_bt)Mo_2_@CPE	—	no	acidic	1.264 × 10^−6^ M	NR	Wang et al. (2020b)
Co^II^(H_2_bdpm)Mo_2_@CPE	—	no	acidic	4.26 × 10^−8^ M	NR	Wang et al. (2020b)
Ag(Py_2_Piz)_2_PW_12_@GCE	a	no	acidic	2.2 × 10^−4^ M	NR	Mou et al. (2020)
Ag_4_(AcyPh_)4_SiMo_12_@GCE	a	no	acidic	2.0 × 10^−4^ M	NR	Mou et al. (2020)
Ag_2_(Py_3_Piz)_2_(H_2_O)_2_SiMo_12_@GCE	a	no	acidic	2.26 × 10^−4^ M	NR	Mou et al. (2020)
Ag/Py_2_TTz/PMo_12_@GCE	a	no	acidic	1.2 × 10^−5^ M	NR	Mou et al. (2020)
[Cu(MET)_2_]Mo_8_@CPE	j	no	acidic	8.45 × 10^−5^ M	NR	Zhang et al. (2021a)
[Cu(bpy)]Mo_2_@CPE	—	no	acidic	8.75 × 10^−4^ M	NR	Zhang et al. (2021a)
Cu_3_(OH)_4_(Ptla)_2_TeM_o6_@CPE	c	no	acidic	1.57 × 10^−4^ M	NR	Ying et al. (2021)
Cu_2_(OH) (Ptep)_2_Mo_8_@CPE	—	no	acidic	1.02 × 10^−2^ M	NR	Ying et al. (2021)
{Cu^I^_5_ [4-atrz]_6_}^5+^-PMo_12_@GCE	a	no	acidic	1.3 × 10^−5^ M	1,000 cycles	Yang et al. (2021)
{Cu^I^_5_ [4-atrz]_6_}^5+^-PW_12_@GCE	a	no	acidic	2.2 × 10^−5^ M	1,000 cycles	Yang et al. (2021)
{Cu^I^_5_ [4-atrz]_6_}^5+^-SiW_12_@GCE	a	no	acidic	1.2 × 10^−5^ M	1,000 cycles	Yang et al. (2021)
(bdpy)PW_11_Co/MWCNTs-COOH@GCE	a	mineral and industrial water	1.5	0.63 μM	220 cycles/1 month	Karimi-Takallo et al. (2021)

Abbreviations as reported by the authors. [C_12_Py][PF_6_], n-dodecyl pyridinium hexafluorophosphate; 4-atrz, 4- amino-triazole; AuNPs, Au nanoparticles; BC, bacterial cellulose; BDDE, boron doped diamond electrode; bdpy, 1,10-(1,4-Butanediyl)dipyridinium; bpy, 4,40-bipyridyl; bte, 1,2-bis(1,2,4-triazol-1-yl)ethane; btmc, 1,4-bis(1,2,4-triazol-1-methyl)cyclohexane; CNTs, carbon nanotubes; CPE, carbon paste electrode; ctcm, C-[4-(1,2,4)Triazol-4-ylmethylcyclohexyl]-methylamine; dbt, 2,2′-dimethyl-4, 4′-bithiazole; dm_4_bt, 2,2′-dimethyl-4,4′-bithiazole; GCE, glassy carbon electrode; H_2_bdpm, 1,1′-bis(3,5-dimethyl-1H-pyrazolate)methane; ILC_n_, CH_3_N(CH_2_CH_2_OH)_2_(C_n_H2_n+1_) Br (*n* = 4, 8, 12); ITO, indium tin oxide electrode; L^a^, 2,3-diphenylpyrazine; L^b^, 2,3-diphenylquinoxaline; mct, 4-(4-Methyl-cyclohexylmethyl)-4H-[1,2,4]triazole; MET, 4-(3-imidazol-1-yl-ethyl)-4H-[1,3,4]triazole; MWCNTs, multi walled carbon nanotubes; NR, not reported; OMC, ordered mesoporous carbon; ox-SWCNts, oxidized single walled carbon nanotubes; PANI, polyaniline; PDDA, poly diallyl dimethyl ammonium; pdp, 4-propyl-4,5-dihydro-1H-pyrazole; PE, plastic electrode; PEDOT, poly(3,4-ethylenedioxythiophene); PEI, Poly(ethyleneimine); PPD, p-phenylenediamine; PSS, poly(styrenesulfonate); Ptep, 1-[2-(3-pyridin-4-yl-[1,2,4]triazol-4-yl)-ethyl]-piperazine; Ptla, 2-[3-pyridin-4-yl-(1,2,4)triazol-4-yl]-ethylamine; PVA, poly(vinyl alcohol); Py_2_Piz, 4,5-bis(2-pyridinyl)imidazole; Py_2_TTz, 2,5-bis(4-pyridyl)thiazolo[5,4-*d*]thiazole; Py_3_Piz, 2-(4-pyridyl)4,5-di(2-pyridinyl)imidazole; rGO, reduced graphene oxide; SWCNTs, single walled carbon nanotubes.

POM archetype according to the legend of Figure 2: a) Keggin, b) Dawson, c) Anderson, j) *γ*-octamolybdate, and -) unspecified type.

##### 2.1.2.3 Sensing of Other Oxidant Species

Bromate is generally found in drinking water as a by-product of ozone disinfection, and it is widely used as a food additive for the maturation of flour and the production of fish paste and fermented beverages. Yet, bromate is a carcinogen. Iodine is an essential micronutrient, which is a crucial part of the thyroid hormones that play an essential role in the development of brain function and cell growth. Potassium iodate has been extensively used for the iodination of commercial table salts as a source of iodine. Deficiency or excess of iodine can cause serious health problems. These are examples of the importance of some of the ionic species (non-metallic oxides) listed in Table 3, and for which several electrochemical sensors based on POMs have been designed. Table 3 also includes information about the POM-hybrid materials used to modify the electrodes (POM-hybrid@electrode), the POM archetype and the figures of merit obtained with those electrodes when analysing standard solutions (more details can be found in Supplementary Table S3 in Supplementary Material). A small number of the reported studies include analysis of real samples. Again, the synergetic effect of the different combinations of POMs, carbon materials, organic compounds, ionic liquids, and metals allowed to improve the limits of detection of the CMEs.

**TABLE 3:** POM-based electrochemical sensors for other oxidants species.

Target	Hybrid material@Electrode	POM archetype	Matrix	pH	Limit of detection	Stability studies	References
BrO_3_^−^	MWNTs/PMo_12_@PGE	a	no	acidic	0.5 μM	NR	Li et al. (2006)
	P_2_Mo_18_/OMC@GCE	b	no	acidic	0.922 μM	NR	Zhou et al. (2007)
	SWCNT/SiMo_12_/[Cu(bpy)_2_]^2+^@GCE	a	no	1	1.1 nM	30 days	Salimi et al. (2009)
	SiNiW_11_/cysteamine@Au	a	no	acidic	14.88 μM	no	Chen et al. (2009)
	Cu_2_(H_2_bdpm)_2_P_2_W_18_@CPE	b	no	acidic	1.8 × 10^−5^ M	NR	Tian et al. (2018b)
	(Cu_3_(pdp)_6_Cl_2_)PCuMo_11_@CPE	a	no	acidic	2.3 × 10^−6^ M	NR	
	Ag_4_L^a^_5_SiW_12_@CPE	a	no	acidic	5.61 × 10^−6^ M	NR	Tian et al. (2018a)
	Ag_3_L^a^_4_PW_12_@CPE	a	no	acidic	1.69 × 10^−5^ M	NR	
	Ag_6_L^b^_6_PMo_12_@CPE	a	no	acidic	2.28 × 10^−6^ M	NR	
	MWCNTs/[C_12_Py][PF_6_]/PMo_12_@GCE	a	no	1	21 μM	100 cycles a day/5 days	Feizy and Haghighi, (2019)
	SWNTs/ILC_12_/PMo_12_@GCE	a	no	acidic	1.3 μM	100 cycles	Wang et al. (2019b)
	SWNTs/ILC_8_/PMo_12_@GCE	a	no	acidic	1.3 μM	100 cycles	
	SWNTs/ILC_4_/PMo_12_@GCE	a	no	acidic	1.3 μM	100 cycles	
IO_3_^−^	P_2_Mo_18_/OMC@GCE	b	no	acidic	0.377 μM	NR	Zhou et al. (2007)
	MWCNTs/[C_8_Py][PF_6_]/PMo_12_@GCE	a	no	2.59	15 μM	20 times a day/5 days	Haghighi et al. (2010)
	CoSal/SiW_12_@CPE	a	no	0.5	48 nM	50 cycles	Kakhki and Shams, (2013)
	PMo_12_/PEI@ITO	a	table salt	5	0.1 μg ml^−1^	100 cycles	Hao et al. (2017)
	P_2_W_17_V/CNTs/CuONPs	b	table salt	2.5	1.5 × 10^−8^ M	100cycles/60 days	Wang et al. (2018b)
	MWCNTs/[C_12_Py][PF_6_]/PMo_12_@GCE	a	no	2.50	2 μM	100 cycles day/5 days	Feizy and Haghighi, (2019)
	SWNTs/ILC_12_/PMo_12_@GCE	a	no	acidic	0.9 μM	100 cycles	Wang et al. (2019b)
	SWNTs/ILC_8_/PMo_12_@GCE	a	no	acidic	0.9 μM	100 cycles	
	SWNTs/ILC_4_/PMo_12_@GCE	a	no	acidic	0.9 μM	100 cycles	
	(bdpy)SiNiW_11_/P-rGO@GCE	a	mineral and tap water, iodized salt	1.5	0.47 nM	200cycles/1 month	Sharifi et al. (2021)
IO_4_^−^	MWCNTs/[C_12_Py][PF_6_]/PMo_12_@GCE	a	no	2.50	4 μM	100 cycles a day/5 days	Feizy and Haghighi, (2019)
ClO_3_^−^	PMo_11_ V/PR@ITO	a	no	2.5	220 μM	8 weeks	Trammell et al. (2017)
	MWCNTs/[C_12_Py][PF_6_]/PMo_12_@GCE	a	no	1	486 μM	100 cycles a day/5 days	Feizy and Haghighi, (2019)
S_2_O_8_^2-^	SiMo_12_/rGO@ITO	a	tap water	acidic	0.129 μM	100 cycles	Guo et al. (2014)
	SiMo_12_/CS/rGO@ITO	a	tap and lake water	acidic	0.05 μM	10 min	Guo et al. (2015b)
	SiMo_12_/PEDOT/rGO@ITO	a	tap and lake water	acidic	0.48 μM	15 min	Guo et al. (2020)
PO_4_^3-^	Mo_8_@PE	—	saline and seawater	acidic	6.1 nM	NR	Figueredo et al. (2021)

Abbreviations as reported by the authors. [C_12_Py][PF_6_], n-dodecyl pyridinium hexafluorophosphate; [C_8_Py][PF_6_], n-Octylpyridinium hexafluorophosphate; Au, gold electrode; bdpy, 1,10-(1,4-butanediyl)dipyridinium; bpy, 4,40-bipyridyl; CNTs, carbon nanotubes; CoSal, N,N′-bis(salicylidene)-1,2-phenylenediaminocobalt (III); CS, chitosan; CuONPs, CuO nanoparticles; GCE, glassy carbon electrode; H_2_bdpm, 1,1′-bis(3,5-dimethyl-1H-pyrazolate)methane; ILCn, CH_3_N(CH_2_CH_2_OH)_2_(C_n_H_2n+1_) Br (*n* = 4, 8, 12); ITO, indium tin oxide electrode; L^a^, 2,3-diphenylpyrazine; L^b^, 2,3-diphenylquinoxaline; NR, not reported; OMC, ordered mesoporous carbon; pdp, 4-propyl-4, 5-dihydro-1H-pyrazole; PE, plastic electrode; PEDOT, poly(3,4-ethylenedioxythiophene); PEI, Poly(ethyleneimine); P-rGO, phosphorus-doped electrochemically reduced graphene oxide; PGE, pencil graphite electrode; PR, *para*-rosaniline acetate dye; SWCNTs, single walled carbon nanotubes.

POM archetype structure according to the legend of Figure 2: a) Keggin, b) Dawson and -) unspecified type.

The bifunctional sensor previously reported for H_2_O_2_ by Salimi et al. (2009), that used a combination of a Keggin SiMo_12_, SWCNTs, and a cationic copper complex (listed as SWCNTs/SiMo_12_/[Cu(bpy)_2_]^2+^@GCE in Table 3) did also present an excellent electrocatalytic activity towards the reduction of BrO_3_^−^ at lower over-potential, due to the copper-complex that catalyses the reduction of bromate. Linear range, detection limit, and stability were 10–200 nM, 1.1 nM, and 30 days, respectively. Even so, no interference or validation studies with real samples were reported.

Sharifi et al. (2021) reported a modified GCE with a tetra-component nanocomposite consisting of a [1,10-(1,4-butanediyl) dipyridinium] ionic liquid (bdpy), the Keggin-type SiW_11_O_39_Ni(H_2_O) (abbreviated as SiW_11_Ni), and Phosphorus-doped electrochemically reduced graphene oxide (P-rGO), by electrodeposition technique for iodate determination. The presence of bdpy provided an additional advantage for increasing the loading of POM, improving the stability of the nanocomposite due to the strong electrostatic attraction between SiW_11_Ni and positively charged bdpy. The (bdpy)SiW_11_Ni/P-rGO@GCE sensor, as listed in Table 3, showed very good stability (1 month), good repeatability (200 cycles), and reproducibility. Furthermore, the modified electrode showed improved analytical figures of merit, such as low limit of detection (0.47 nM), high sensitivity (28.1 μA mM^−1^), good selectivity, and a wide linear range (10–1,600 μM^−1^), compared to other CMEs. It was validated by measuring IO_3_^−^ in mineral and tap water and in a commercial edible iodized salt, proving that it could be efficiently applied to quantifying trace-level IO_3_^−^ in real samples.

It is noteworthy to highlight another POM-based sensor listed in Table 3: a plastic electrode (PE) decorated with a tetrabutylammonium (TBA) derivative salt of octamolybdate, [N(C_4_H_9_)_4_]_4_Mo_8_O_26_, reported as MO_8_@PE and used for PO_4_^−^ detection (Figueredo et al., 2021). This POM-based sensor, specially designed with TBA to be only soluble in organic solvents, achieved a remarkably LOD of 6.1 nM, better than the conventional analytical approaches reported by the authors. Plus, the standard spectrophotometric method used for PO_4_^3-^ detection takes 1 h for colour to develop. In contrast, this single-use MO_8_@PE sensor takes less than 5 min to show a result, and it was successfully applied in saline and seawater samples, being an affordable alternative for phosphate determination.

##### 2.1.2.4 Sensing Biomolecules and Bio-Related Species

The excellent biocompatibility of POMs makes them extremely valuable in electrochemical biosensors, and POM-composite materials have been reported as highly successful electrocatalysts for the oxidation of biomolecules and bio-related species. Table 4 shows a list of POM-based electrodes used for a series of biomolecules, including the respective information about the POM-hybrid materials used to modify the electrodes (POM-hybrid@electrode), the POM archetype, and the figures of merit obtained with those electrodes when analysing standard solutions (more details can be found in Supplementary Table S4 in Supplementary Material). Among those biomolecules reported in Table 4, dopamine (DA) is a naturally occurring catecholamine that plays a very important role as a neurotransmitter in the mammalian central nervous system and plays a central role in Parkinson’s disease. For a healthy individual, the DA level lies in the range of 0.01–10 μM (Thakur et al., 2018). The easy electro-oxidation of DA turns electrochemical methods attractive. However, the coexistence of uric acid (UA) and a high concentration of ascorbic acid (AA) in the extracellular fluids of the central nervous system can cause significant interference because of its oxidation potentials which are close to that of DA on bare electrodes, resulting in poor selectivity. Accordingly, surface modification of electrodes with suitable electrocatalysts had been used to improve both the sensitivity and the selectivity of DA detection over AA and UA. Thakur et al. (2018) developed a novel POM-based sensor for highly selective and ultra-sensitive detection of DA, using a sandwich POM Na_12_ [WCo_3_(H_2_O)_2_(CoW_9_O_34_)_2_] (abbreviated as Co_5_POM) and poly (vinylimidazolium) cation [PVIM^+^] in combination with nitrogen-doped carbon nanotubes (N-CNTs), listed in Table 4 as PVIM−Co_5_POM/N-CNTs@GE. This combination provides the synergy between PVIM−POM catalyst and N-CNTs as conductive support, which enhances the electron transport at the electrode/electrolyte interface. Besides, it eliminates the interference of AA at physiological pH (7.4). The novel PVIM−Co_5_POM/N-CNTs composite achieved high selectivity and sensitivity, low detection limit (500 pM), and a wide linear working range of 0.0005–600 μM, even in the presence of a higher concentration of AA (500 μM), being one of the best catalysts reported so far for the selective electrochemical detection of DA. In an attempt to explore this sensor for practical applications, the PVIM−Co5POM/N-CNTs composite was analysed for the detection of DA in real sample using commercially available DA hydrochloride injections (40 mg ml^−1^) by standard addition method, and the recovery of the sample was in the range of 95–102%, which demonstrated the applicability of the composite for real-time analysis. The stability of the PVIM−Co_5_POM/N-CNTs composite was demonstrated during 100 cycles. Additionally, Zhou et al. (2013) reported a Dawson-type POM [P_2_W_16_V_2_O_62_]^8-^ decorated by Au-Pd alloy NPs, applied onto ITO electrodes, by LbL self-assembly technique, used to simultaneously determine AA and DA at pH 7 (listed in Table 4 as P_2_W_16_V_2_/Au-PdNPs@ITO). AA is also an important biomolecule present in the mammalian brain, and it is a vital component in the human diet, used for the prevention and treatment of some diseases (common cold, mental illness, infertility, cancer, and AIDS). The sensing composite film exhibited high electrocatalytic activity towards the oxidation of AA and DA by decreasing the oxidation over-potentials and remarkably increasing the peak currents, attributed to the combining effect of P_2_W_16_V_2_ and Au-Pd in the composite film. DA and AA detection limits were 0.83 and 0.43 μM, respectively, and the sensor showed high selectivity and sensitivity. The sensor could be used to determine in real samples. However, the highest sensibility for AA was achieved by Ammam and Easton (Ammam and Easton, 2011b) with a hybrid material based on a 1-butyl-3-methylimidazolium tetrafluoroborate ionic liquid [(BMIM)(BF_4_)] and the Dawson-type ion [P_2_Mo_18_O_62_]_6_^−^, immobilized on glassy carbon electrode (GCE). The resulting AA sensor, [BMIM]_6_-P_2_Mo_18_@GCE as listed in Table 4, presented a significant sensitivity of ∼63 nA/μM to AA, a fast response time (<9 s), low detection limit (<0.1 μM), high selectivity towards endogenous interferences such as uric acid, acetaminophen and DA, a linear range from 0.1 μM to at least 22 mM, and was stable for at least 2 weeks. In addition, this AA sensor operated in a pH range from 0 to at least 7, which was attributed first to the presence of the ionic liquid cation in the hybrid material, and second to the porous morphology of the deposited film, which allowed a facile charge equilibrium within the film.

**TABLE 4:** POM-based electrochemical sensors for biomolecules and bio-related species.

Target	Hybrid material@Electrode	POM archetype	Matrix	pH	Limit of detection	Stability studies	References
Dopamine	P_2_W_16_V_2_/Au-PdNPs@ITO	b	serum	7	0.83 μM	300 cycles	Zhou et al. (2013)
	PMo_9_V_3_/PtNPs@ITO	a	dopamine hydrochloride injection	6.5	1.3 × 10^−7^ M	100 cycles	Li et al. (2013)
	PMo_11_V/PEI/CoTsPc-@ITO	a	blood serum	6.5	1.3 × 10^−8^ M	500 cycles	Zhu et al. (2013)
	PMo_12_/PEI@ITO	a	serum	5	0.2 μg ml^−1^	100 cycles	Hao et al. (2017)
	Cu_3_Mo_5_P_2_/rGO@GCE	—	artificial cerebrospinal fluid, human blood serum	7	80.4 × 10^−9^ M	1 week	Zhang et al. (2017b)
	PMo_9_V_3_/Pd-PtNPs/MWCNTs@ITO	a	human serum and dopamine hydrochloride injections	7.3	1.25 × 10^−8^ M	100 cycles	Jiao et al. (2018)
	PVIM-Co_5_POM^a^/N-CNTs@GE	a	dopamine hydrochloride injections	7.4	500 pM	100 cycles	Thakur et al. (2018)
	GeW_12_/CFMWCNTs/Nafion@GCE	a	no	3.6	1.23 μM	180 cycles	Shi et al. (2019)
	PtNPs/IMo_6_/GO@GCE	c	human serum	1.3	0.22 μM	100 cycles/20 days	Zhang et al. (2019b)
	P_2_W_17_V/CS@ITO	b	human serum	7.0	0.18 μM	100 cycles	Wang et al. (2019c)
	Ce-POM^b^/CFMWCNTs@GCE	—	no	7.0	1.61 μM	180 cycles	Liu et al. (2020b)
	V_10_O_28_/NU-902@FTO	e	no	4.5	2.1 μM	20 cycles	Ho et al. (2020)
	Ce-POM^c^/CFMWCNTs@GCE	—	no	3.0	0.053 μM	100 cycles/7 days	Jiang et al. (2020)
	[Ag_5_ (trz)_4_]_2_·PMo_12_/SWCNTs-COOH@GCE	a	human serum	7.0	8.6 nM	100 cycles/1 month	Zhou et al. (2021)
	PMo_12_ [6]catenane/rGO@GCE	a	human serum	2.0	0.065 μM	50 cycles/1 week	Han et al. (2021)
Ascorbic acid	PEI/RuSiW_10_@ITO	a	no		0.08 mM	NR	Ma et al. (2010)
	[BMIM]_6_-P_2_Mo_18_@GCE	b	no	0–7	<0.1 μM	2 weeks	Ammam and Easton, (2011b)
	SiNiW_11_/cysteamine@Au	a	no		14.60 μM	no	Chen et al. (2009)
	P_2_W_16_V_2/_Au-PdNPs@ITO	b	fruit juice	7	0.43 μM	300 cycles	Zhou et al. (2013)
	PMo_12_/GS@GCE	a	vitamin C tablets	7.2	0.5 × 10^−6^ M	1 month	Zhang et al. (2014)
	PW_12_/PEI@ITO	a	soft fruit drinks	5	6.4 × 10^−4^ mg/ml	NR	Xu et al. (2015)
	PMo_12_/PEI@ITO	a	fruit juice	5	0.43 μg ml^−1^	100 cycles	Hao et al. (2017)
	PtNPs/IMo_6_/GO@GCE	c	human serum	1.3	6.42 μM	100 cycles/20 days	Zhang et al. (2019b)
	P_2_Mo_17_V/Ru (bpy)_3_/CS-PdNPs@ITO	b	juice	7	0.1 μM	30 days	Zhang et al. (2021b)
Creatinine	MIP/AgNPs/PW_12_/rGO@GCE	a	saliva and serum	6	1.51 × 10^−11^ M	10 days	Zhang et al. (2018)
Cholesterol	PVIM-Co_5_POM^a^/N-MPC@GE	a	human blood serum	7.4	1 fM	100 cycles	Thakur et al. (2019)
Bilirubin	MIP/PW_12_/C_3_N_4_NTs@GCE	a	human plasma	4.0	0.1 p.m.	60 days	Yola et al. (2017)
Xanthine	Fc/PMo_6_W_6/_rGO@GCE	a	human urine	6.0	10.1 nM	100 cycles/2 weeks	Zhu et al. (2019)
Glucose	Fe_4_POM^d^/poly (1,8 DAN)/GOx@GE	a	no	2.5	1.2 mM	no	Turdean et al. (2002)
	MPS/B/PFe_3_Mo_9_/B/GOx@Au	a	no	6.2	NR	NR	Turdean and Popescu, (2012)
	PMo_12_/rGO/GOx@GCE	a	no		67.9 μM	NR	Yang et al. (2016a)
	P_2_Mo_18_/PMA/MWCNTs@GCE	b	no	7.0	NR	15 days	Boussema et al. (2018)
	PW_9_/PAAC/GOx@GE	a	Fizzy drink, Cherry juice	6.0	0.099 mM	4 weeks	Ayranci et al. (2018)
	Co_2_W_11_/MWCNTs@GE	a	coke, juice		1.21 μM	5 weeks	Ayranci et al. (2019)
Uric acid	Ce-POM^b^/CFMWCNTS@GCE	a	no	7.0	5.41 μM	180 cycles	Liu et al. (2020b)
	PtNPs/IMo_6_/GO@GCE	c	human serum	1.3	0.72 μM	100 cycles/20 days	Zhang et al. (2019b)
	Cubix/P_2_W_18_@GCE	b	no	6.0	4.97 × 10^−7^ M	50 cycles/30 days	Xu et al. (2021)
	rGO/AuNPs/P_2_W_18_@ITO	b	human serum	7.0	0.15 μM	50 cycles/30 days	Bao et al. (2020b)
	bix/P_2_W_18_@GCE	b	human urine	3.0	5.85 × 10^−7^ M	5 cycles/4 weeks	Liu et al. (2020c)
	AM-LnSTsPOM/CFMWCNTs@GCE	a	no	7.0	1.69 μM	160 cycles	Cui et al. (2021)
NADH	AuNPs/PW_12_/OMC@GCE	a	no	7	0.41 μM	2 weeks	Zhang et al. (2015)
	Ru (bpy)_3_^2+^/PMo_12_@ITO	a	no	7.0	1.67 × 10^−8^ M	21 cycles/2 weeks	Li et al. (2012)
	Ru (bpy)_3_^2+^/PMo_12_/mrGO@mGCE	a	yes	7.4	0.1 nM	28 cycles/1 month	Qian et al. (2014)
*Yersinia pestis*	SiW_11_Sn-dATPs@Au	a	no		0.6 nM	NR	Ortiz et al. (2017)
	SiW_11_Sn-dGTP@Au	a	no		0.3 nM	NR	
	SiW_11_Sn-dATP/dGTP@Au	a	no		0.7 nM	NR	
	P_2_W_17_Sn-dATP@Au	b	no		1.12 nM	NR	
	P_2_W_17_Sn-dGTP@Au	b	no		1.70 nM	NR	
	P_2_W_17_Sn-dATP/dGTP@Au	b	no		1.50 nM	NR	
miRNA21	PMo_12_-MoS_2/_ *β*-FeOOH@Au	a	human serum	7.4	0.11 fM	10 cycles/15 days	Jia et al. (2020)
Guanine and Adenine	PNiW_11_/PDDA/MWCNTs@GCE	a	salmon sperm	2	0.24 μM and	NR	Ensafi et al. (2017)
					0.1 μM	NR	
Osteopotin	PPy/Ti_3_C_2_Tx/PMo_12_@GCE	a	human serum	7.4	0.98 fg ml^−1^	10 cycles/15 days	Zhou et al. (2019)
L-cysteine	VMo_12_/[BMIM][PF_6_]@CPE	a	food supplement		0.085 mM	NR	Ji et al. (2009)
	CoSal/SiW_12_@CPE	a	human serum, urine, N-acetylcysteine effervescent tablets	5.0	4.9 nM	2 months	Kakhki et al. (2013)
	CoSal/SiW_12_@CPE	a	no	5	967 nM	50 cycles	Kakhki and Shams, (2013)
L-tyrosine and L- tryptophan	PW_12_/rGO@GCE	a	human serum	6	2 × 10^−12^ M	45 days	Yokuş et al. (2016)
Folic acid	PPy/PMo_2_W_9_/AuNPs@Au	a	human serum, vitamin supplements	6.0	0.12 nM	NR	Babakhanian et al. (2014)
	PEI/P_2_Mo_16_V_2_/rGO@GCE	b	human serum	7.4	2.84 × 10^−10^ M	60 days	Xu et al. (2017)
Cardiac troponin I	{Mo_368_}/FeOOH/Bi_2_S_3_/AuNPs@ITO	q	human serum		0.76 pg ml^−1^	NR	Bao et al. (2020a)

Abbreviations as reported by the authors.[BMIM][PF_6_], 1-butyl-3-methylimidazolium hexafluorophosphate; AgNPs, silver nanoparticles; AM-LnSTsPOM, alkali-metal–lanthanide embedded selenotungstates; AuNPs, gold nanoparticles; Au-PdNPs, gold and palladium nanoparticles; B, ethylamine; bix, 1,4-bis(imidazol-1-ylmethyl) benzene; CFMWCNTs, carboxyl functionalized multi-walled carbon nanotubes; CoSal, N,N′-bis(salicylidene)-1,2-phenylenediaminocobalt (III); CoTsPc, cobalt(II) tetrasulfonate phthalocyanine; CS, chitosan; CS-PdNPs, Chitosan and palladium nanoparticles; Fc, ferrocene; FTO, fluorine doped tin oxide; GCE, glassy carbon electrode; GO, graphene oxide; GOx, glucose oxidase; GS, graphene sheets; ITO, indium tin oxide electrode; mGCE, magnetic glassy carbon electrode; MIP, molecularly imprinted polymer; MPS, 3-mercapto-1-propanesulfonic acid; mrGO, magnetic reduced graphene oxide; MWCNTs, multi walled carbon nanotubes; N-CNTs, nitrogen-doped carbon nanotubes; N-HCSs, nitrogen-doping hollow carbon spheres; N-MPC, nitrogen-doped mesoporous carbon; NR, not reported; OMC, ordered mesoporous carbon; PAAC, 3-Amino-9-ethylcarbazole polymer film; PDDA, poly diallyl dimethyl ammonium; PdNPs, palladium nanoparticles; PEI, Poly(ethyleneimine); PMA, 1-pyrenemethylamine; PPy, polypyrrole; PtNPs, platinum nanoparticles; Pt-PdNPs, platinum and palladium nanoparticles; PVIM^+^, poly(vinylimidazolium) cation; rGO, reduced graphene oxide; SWCNTs-COOH, carboxyl functionalized single walled carbon nanotubes; trz, 3-mercapto-1, 2,4-triazole.

a Na_12_ [WCo_3_(H_2_O)_2_(CoW_9_O_34_)_2_].

b [H_2_N(CH_3_)_2_]_8_Na [CeNa(H_2_O)_4_(OH)WO(H_2_O) (B-*α*-SeW_9_O_33_)2]⋅18H_2_O.

c Na_16_H_6_{[Ce_3_W_4_O_10_(H_2_O)_9_ (CH_3_COO)_3_]_2_ (Se_2_W_7_O_30_) (B-*α*-SeW_9_O_33_)_4_}·(C_5_H_8_NBO_3_)·119H_2_O.

d Na_6_ [H_4_Fe_4_(PMo_9_O_34_)_2_(H_2_O)_2_].H_2_O.

POM archetype structure according to the legend of Figure 2: a) Keggin, b) Dawson, c) Anderson, d) Lindqvist, e) decavanadate, and also including q) hedgehog-shape, and -) unspecified type.

Glucose amperometric biosensors, based on the immobilization of the glucose oxidase enzyme (GOx) on POM-hybrid composites, have been reported in the literature (Turdean et al., 2002; Turdean and Popescu, 2012; Yang et al., 2016a; Boussema et al., 2018; Xu et al., 2017), and are listed in Table 4. However, the best sensor performance for glucose detection was achieved by Ayranci et al. (2019) with a non-enzymatic electrochemical sensor. The Keggin-type K_7_ [Co^III^Co^II^(H_2_O)W_11_O_39_].15H_2_O, abbreviated as Co_2_W_11_, composed of unique mixed-valence Co(III) and Co(II) structures, was confined in a matrix of multi-walled carbon nanotubes (MWCNTs) on graphite electrodes (GEs) (listed in Table 4 as Co_2_W_11_/MWCNTs@GE). The proposed non-enzymatic sensor showed a wide linear range, from 0.1 to 10.0 mM of glucose. Besides, it exhibited a low detection limit of 1.21 μM, a fast response time of 6 s, high sensitivity (256.4 μA mM^−1^ cm^−2^), and good stability (5 weeks). These good results were explained by the authors with the improvement of electroactive surface area and the synergistic electrocatalytic activity resulting from the combination of Co-POM and MWCNTs.

Another successful non-enzymatic electrochemical sensor was reported by Thakur et al. (2019) for cholesterol. Cholesterol is an essential lipid of the human body and remains one of the most frequently analysed in clinical practice due to its association with various cardiovascular and brain disorders. The normal level of total cholesterol in blood serum is approx. 200 mg dl^−1^ and values higher than 240 mg dl^−1^ are responsible for damage of the arteries and diseases such as arteriosclerosis, heart diseases, hypertension, and cerebral thrombosis. Thakur (Thakur et al., 2019) proposed a non-enzymatic electrochemical sensor for cholesterol based on a sandwich POM [WCo_3_(H_2_O)_2_-(CoW_9_O_34_)_2_]^12-^ (Co_5_POM) combined with poly (vinylbutylimidazolium) [PVIM^+^], which acted as a conductive matrix, simultaneously balancing the high negative charge (−12) of the POM. After, the PVIM–Co_5_POM conjugate was supported on nitrogen-rich mesoporous carbon (N-MPC) materials to enhance the activity. The modified graphite electrode (GE), listed in Table 4 as PVIM-Co_5_POM/N-MPC@GE, demonstrated high selectivity and sensitivity for cholesterol, with a wide detection range from 1 fM to 5 mM and a response time around 5 s. The linear response for cholesterol ranged from 1 fM to 200 nM, and sensitivity was 210 μA mM^−1^ cm^−2^. Moreover, interferent species, such as glucose, UA, and AA, showed no significant effect on cholesterol sensing. The sensor was applied for the quantitative analysis of cholesterol in human blood serum at physiological pH.

All these successful examples of non-enzymatic sensors for important biological species proved that the drawback of enzyme-based sensors, such as restricted immobilization and easy inactivation, can be overcome, and POM-hybrid materials may be precursors for producing non-enzymatic electrode materials in the coming years.

Furthermore, POM-based aptasensors, meaning biosensors that use aptamers as recognition elements, have been developed. Jia et al. (2020) reported a novel nanohybrid of polyoxometalate-derived MoS_2_ nanosheets (PMO_12_-MoS_2_ NSs) tightly and vertically grown over *ß*-FeOOH nanorods (NRs) that were exploited as platforms to immobilize the complementary DNA (cDNA) strands of microRNA-21 (miRNA) for further detection. Compared with other sensing systems referred by Jia et al. (2020), the PMO_12_-MoS_2_/*β*-FeOOH@Au modified electrode (as listed in Table 4) had superior sensing performance toward miRNA-21 with an incredible detection limit of 0.11 fM, a broad linear range from 1 fM to 5 nM, high selectivity, good stability (15 days), excellent reproducibility, and acceptable feasibility. Bao et al. (2020a) also reported a photoelectrochemical sensor based on the matrix FeOOH/Bi_2_S_3_/AuNPs and using the hedgehog-shape{Mo_368_} cluster as an electron donor for the ultrasensitive detection of cardiac troponin I (cTnI). Combined {Mo_368_}/FeOOH/Bi_2_S_3_/AuNPs with the specific recognition of antigen and antibody, a novel sensor based on a modified ITO, and listed in Table 4 as {Mo_368_}/FeOOH/Bi_2_S_3_/AuNPs@ITO, was constructed, showing a wide detection range of 1.00 pg ml^−1^–100 ng ml^−1^ and a low detection limit (0.76 pg ml^−1^). In general, the content of cTnI in normal human serum is below 0.2 ng ml^−1^, but direct damage to the myocardium occurs when the concentration of cTnI is higher than 2.0 ng ml^−1^. Therefore, the new sensor was able to detect cTnI at the early stages of cardiovascular disease. Despite the complex preparation procedures, these new strategies can open new routes for biosensing in clinical diagnosis by detecting other targets for which suitable probes (biomarkers) need to be anchored. Table 4 list POM-based electrochemical sensors for other biomolecules such as creatinine, bilirubin, xanthine, L-tyrosine, and L-tryptophan, among others. These sensors have in common to operate in the nM or pM range, and most of them were evaluated for stability and interferents and applied to real sample analysis, with reliable results, showing to be valuable alternatives to more costly and sophisticated analytical techniques.

##### 2.1.2.5 Sensing Medicines, Pesticides and Toxic Contaminants

Table 5 summarises the POM-based composite electrochemical sensors developed for medicines, pesticides, and toxic contaminants, listing the details about the POM-hybrid material used to modify the electrode (POM-hybrid@electrode), the POM archetype, working pH, the limit of detection, the stability of the sensor and information about tests with real samples (more details can be found in Supplementary Table S5 in Supplementary Material). Highlights go to an ultrasensitive electrochemical sensor for the selective measurement of trace ceftizoxime (CFX), proposed by Rouhani and Soleymanpour (Rouhani and Soleymanpour, 2021), using a thin film of Preyssler nanocapsules (PNCs) on pencil graphite electrode (PGE) surface modified with reduced graphene oxide (rGO). Under the optimized conditions, the PNCs/rGO@PGE sensor, as listed in Table 5, presents a wide linear concentration range, from 1.0 × 10^−11^ to 3.0 × 10^−8^ M, and an excellent detection limit of 1.8 pM. The outstanding electrochemical performance of the PNCs/rGO@PGE sensor was related to the synergistic influence of PNCs/r-GO/PGE thin film, which enhanced efficiency in drug encapsulation, stability, and effective surface area of the electrode for the CFX oxidation. The novel sensor showed better sensitivity than the earlier reported techniques for the CFX measurement, and it was successfully used to determine the trace amounts of CFX in pharmaceutical formulations and blood serum with suitable recoveries.

**TABLE 5:** POM-based electrochemical sensors for medicines, pesticides, and toxic contaminants.

Target	Hybrid material@Electrode	POM archetype	Matrix	pH	Limit of detection	Stability studies	References
Clenbuterol and Ractopamine	PV_8_Mo_4_/ZrO_2_@GCE	a	pork	1.0	5.03 × 10^−9^ M and 9.3 × 10^−7^ M	2 weeks	Zhang et al. (2019c)
Acetaminophen	AuNPs/PW_12_/OMC@GCE	a	paracetamol tablets	7	0.29 μM	NR	Zhang et al. (2015)
	PMo_11_V/N-CNTs@GCE	a	no	2.5	1.0 × 10^−6^ M	NR	Fernandes et al. (2017)
	PdNPs/PW_12_/N-HCSs@GCE	a	paracetamol tablets	7.4	3 nM	1 h/2 weeks	Wang et al. (2019d)
	La-GeW_12_/CFMWCNT@GCE	a	no	8.0	1.07 μM	180 cycles	Li et al. (2019)
	Tb-GeW_12_/CFMWCNT@GCE	a	no	8.0	1.08 μM	180 cycles	
	AuNPs/SiW_11_Cu/MWCNTs@GCE	a	paracetamol tablets, mineral and river water	7	0.42 μM	12 days	Dong et al. (2019)
	Ce-POM^a^/CFMWCNTs@GCE	—	no	3.0	2.03 μM	100 cycles/7 days	Jiang et al. (2020)
Triclosan	AuNPs/PW_12_/rGO@GCE	a	wastewater, lake water	7.0	0.15 nM	30 days	Yola et al. (2015)
Ceftizoxime	PNC/rGO@PGE	k	ampoules, blood serum	3.0	1.8 p.m.	1 month	Rouhani and Soleymanpour, (2021)
Methyldopa	PMo_12_/rGO@PGE	a	human blood serum, urine, and milk	2.8	1.2 × 10^−10^ M	2 weeks	Dehnavi and Soleymanpour, (2020)
Paroxetine	PW_12_/rGO@PGE	a	paroxetine tablets, human serum, urine	7.0	9.0 × 10^−10^ M	15 days	Oghli and Soleymanpour, (2020)
Sildenafil	MIP/AuNPs/NaP_5_W_30_/MWCNTs@PGE	k	human plasma, milk	7.0	0.033 nM	10x, 1 month	Rouhani and Soleymanpour, (2020)
Simazine	MIP/PtNPs/PW_12_/MWCNTs@GCE	a	industrial wastewater	4.0	2.0 × 10^−11^ M	NR	Ertan et al. (2016)
Hydrazine	P_2_W_17_Fe/PdNPs@ITO	b	no	2	1.5 μM	1 month	Ma et al. (2012)
Hydrazine sulfate and Nitrobenzene	PtNPs/PMo_12_/OMC@GCE	a	no	7	3.41 μM and 3.82 μM	2 weeks	Li et al. (2016)
Hydroquinone, Catechol and Resorcinol	rGO/SiW_12_@GCE	a	diphenolic compounds, underground and lake water	4.5	50 nM, 40 nM and 90 nM	6 weeks	Cao et al. (2011)
N-hydroxysuccinimide	PtNPs/PW_12_/2D-hBN@CPE	a	drinking, lake, and river water	8.0	60 nM	45 days	Karimi-Maleh et al. (2020)
Chlorogenic acid	AuNPs/PW_12_/MacroPC@GCE	a	pharmaceutical	7.0	2.15 nM	2 weeks	Zhang et al. (2017c)
Mycertin	P_2_W_18/_SnO_2/_AuNPs@ITO	b	juice	3	67 nM	20 cycles/1 week	Xing et al. (2019)
Ochratoxin A	MIP/AgNPs/PW_12_/rGO@GCE	a	grape juice and wine	6.0	1.6 × 10^−11^ M	30 days	Yola et al. (2016)
Citrinin	MIP/PtNPs/PW_12_/rGO@GCE	a	rye samples	6.0	2.0 × 10^−13^ M	45 days	Atar et al. (2016)
Propylparaben	PPy/β-CD/PMo_12_@PGE	a	cleansing micellar solution	6.0	0.04 μM	5 cycles	Hatami et al. (2021)
Diphenylamine	PMo_12_/GO@GCE	a	apple juice	7.0	6.0 nM	2 weeks	Gao et al. (2020b)
Diazinon	MIP/AuNPs/PW_12_/2D-hBN@GCE	a	fruit juice	6.0	3.00 × 10^−12^ M	45 days	Medetalibeyoğlu et al. (2020)
Bisphenol A	AgPMo_12_@Au	a	river water, milk, human serum	7.4	0.2 fg ml^−1^	7 cycles/15 days	Song et al. (2020)
	AuNPs/SiW_11_Cu/MWCNTs@GCE	a	Mineral and local river water	7	0.89 μM	12 days	Dong et al. (2019)
*γ*-Lindane	MIP/PW_12_/C_3_N_4_NTs@GCE	a	orange juice	7.0	2.0 × 10^−11^ M	60 cycles/60 days	Pelin Böke et al. (2020)

Abbreviations as reported by the authors. 2D-hBN, two dimensional hexagonal boron nitride nanosheets; *β*-CD, *ß*-cyclodextrin; AgNPs, silver nanoparticles; Au, gold electrode; AuNPs, gold nanoparticles; C_3_N_4_NTs, carbon nitride nanotubes; GCE, glassy carbon electrode; GO, graphene oxide; ITO, indium tin oxide electrode; MacroPC, macroporous carbon; MIP, molecularly imprinted polymer; MWCNTs, multi-walled carbon nanotubes; N-CNTs, nitrogen-doped carbon nanotubes; NR, not reported; OMC, ordered mesoporous carbon; PdNPs, palladium nanoparticles; PGE, pencil graphite electrode; PNC, preyssler nanocapsules; PPy, polypyrrole; PtNPs, platinum nanoparticles; rGO, reduced graphene oxide.

a Na_16_H_6_{[Ce_3_W_4_O_10_(H_2_O)_9_-(CH_3_COO)_3_]_2_(Se_2_W_7_O_30_) (B-*α*-SeW_9_O_33_)_4_}·(C_5_H_8_NBO_3_)·119H_2_O.

POM archetype structure according to the legend of Figure 2: a) Keggin, b) Dawson, and including k) Preyssler, and -) unspecified type.

POM-based electrochemical sensors have been reported using molecularly imprinted polymers (MIPs) to attain the selectivity required to determine hazardous compounds. Besides the MIP, they have been combined with carbon materials and metal nanoparticles to overcome the restrictions in the conductivity of POMs. A MIP is a polymer that has been synthesized using a molecular imprinting technique with a mould molecule, leaving cavities in the polymer matrix with an affinity for that chosen mould molecule. POM hybrid materials combined with MIPs, mostly Keggin-type, have been reported for sildenafil (Rouhani and Soleymanpour, 2020), simazine (Ertan et al., 2016), ochratoxin A (Yola et al., 2016), and *γ*-lindane (Pelin Böke et al., 2020), and are listed in Table 5, all presenting excellent detection limits (10^−11^ M) and wide linear ranges. The sensors have been tested for common interferents and validated by testing their application in real sample analysis. Emphasis goes to POM/MIP-based electrochemical sensors for diazinon and citrinin, which proved to be able to determine these targets in food samples. Diazinon (DIA) is an organophosphorus pesticide and is considered very risky and harmful because of its noxious nature. Their recognition at ultra-trace levels in environmental samples and foodstuff is a serious analytical challenge. Medetalibeyoğlu *et al.* (Medetalibeyoğlu et al., 2020) reported the use of gold nanoparticles (AuNPs) incorporated Keggin-type POM/two-dimensional hexagonal boron nitride (2D-hBN) nanosheets and molecularly imprinted polymer (MIP) for the electrochemical detection of DIA molecule in fruit juice samples. The modified GCE electrode, listed as MIP/AuNPs/PW_12_/2D-hBN@GCE in Table 5, showed high selectivity and stability, and reliability for DIA detection. The linearity range and detection limit were 1.00 × 10^−11^ -1.00 × 10^−8^ M and 3.0 × 10^−12^ M, respectively, and sensitivity was better than other reported sensors (Medetalibeyoğlu et al., 2020). Another ultra-sensitive POM/MIP-based electrochemical sensor was reported for Citrinin (CIT). CIT is a toxic mutagenic and carcinogenic secondary metabolite of fungi, resistant to decomposition, and it is found in diverse food samples such as cheese, barley, red yeast rice, and apples. Atar et al. (2016) reported a molecular imprinted voltammetric sensor for CIT based on GCE modified with platinum nanoparticles (PtNPs), involving the Keggin polyoxometalate H_3_PW_12_O_40_ functionalized with reduced graphene oxide (rGO). The developed system, listed in Table 5 as MIP/PtNPs/PW_12_/rGO@GCE, showed a performance comparable to other CIT-imprinted sensors and conventional analytical methods. It is ultra-sensitive, with a detection limit of 2.0 × 10^−13^ M, rapid, easy, shows very good stability (45 days), and it might be preferred to other published methods.

##### 2.1.2.6 Sensing Heavy Metals

Heavy metals have been early identified as primary environmental contaminants due to their non-biodegradability, bioaccumulation, and toxicity. Because of their harmful effects above the permissible limits, it is necessary to measure the concentration of these heavy metals to preserve the environment and health of individuals. Traditional methods for metal analysis include UV-Vis absorption spectrometry, surface-enhanced Raman spectrometry (SERS), atomic absorption spectrometry (AAS), atomic fluorescence spectrometry (AFS), ion chromatography (IC), inductively coupled plasma mass spectrometry (ICP-MS), and inductively coupled plasma optical emission spectrometry (ICP-OES). However, these techniques require expensive instruments, costly operations, and well-skilled operators to perform the multi-step sample preparation and complex analytical procedures, which are unsuitable for on-site and timely measurements necessary to monitor transient phenomena.

Hexavalent chromium (VI) is one of the most toxic heavy metal ions with high solubility in water. It has gained wide attention due to its high poisonousness and mutagenic-carcinogenic effects on human health. Therefore, the Cr(VI)-containing compounds were listed as human carcinogens by International Agency for Research on Cancer (IARC), and the World Health Organization (WHO) has established a maximum permissible concentration of total Cr(VI) in drinking water and industrial water of 0.05 and 0.5 ppm, respectively. POM-based electrochemical sensors were reported as alternatives for metal analysis, especially for chromium (VI), and are summarized in Table 6. Table 6 includes the respective information about the POM-hybrid materials used to modify the electrodes (POM-hybrid@electrode), the POM archetype, and the figures of merit obtained with those electrodes when analysing standard solutions (more details can be found in Supplementary Table S6 in Supplementary Material). Recently, Niu et al. (2021) reported the synthesis of two hourglass-type phosphomolybdate hybrids with different heterometallic centres, achieving an efficient electrochemical detection of ultra-trace Cr(VI) in wide pH ranges of 0–5. The reported sensors, {P_4_Mo_6_}/Cu/Mn/BBTZ@GCE (1) and {P_4_Mo_6_}/Na/Mn/BBTZ@GCE (2), at pH 0 displayed prominent sensitivities of 111.08 μA μM^− 1^ and 119.87 μA M^−1^, along with ultra-low detection limits towards Cr(VI) of 1.59 nM (0.17 ppb) and 2.91 nM (0.30 ppb), respectively, which fully satisfy the WHO standards for drinking water. The activity origin of both hybrids for impressive electrochemical behaviours was originated from the synergistic effect between reduced {P_4_Mo_6_} cluster and heterometallic centres at the molecular level. In the pH range of 1–5, good sensitivities and low detection limits (<25 nM) were also achieved by both sensors. Moreover, both were insensitive to common interferences and very stabile.

**TABLE 6:** POM-based electrochemical sensors for metal ions.

Target	Hybrid material@Electrode	POM archetype	Matrix	pH	Limit of detection	Stability studies	References
Cr^6+^	Co/{P_4_Mo_6_}_2_@GCE	r	lake water	acidic	0.026 μM	5.5 h	Wang et al. (2020c)
	Ni/{P_4_Mo_6_}_2_@GCE	r	no		0.321 μM	NR	
	Cd/{P_4_Mo_6_}_2_@GCE	r	no		0.082 μM	NR	
	Cu^II^_4_(btmc) (ctcm)_4_Mo_8_@ CPE	—	no	acidic	7.4 × 10^−8^ M	NR	Wang et al. (2020b)
	Cu^II^_4_(mct)_2_ (ctcm)_2_(H_2_O)_6_Mo_8_@CPE	—	no		2.5 × 10^−7^ M	NR	
	Cu^II^(dm_4_bt)Mo_3_@CPE	—	no		6.5 × 10^−7^ M	NR	
	Co^II^(dm_4_bt)Mo_2_@CPE	—	no		7.35 × 10^−6^ M	NR	
	Co^II^(H_2_bdpm)Mo_2_@CPE	—	no		1.03 × 10^−6^ M	NR	
	(H_2_bpp)_2_ [Na_4_Fe(H_2_O)_7_]FeP_4_Mo_6_@GCE	r	lake water	acidic	0.174 μM	NR	Xin et al. (2020)
	(H_2_bpp)_6_ (bpp)_2_]FeP_4_Mo_6_@GCE	r	no		0.33 μM	NR	
	H_3_ [Cu_2_(4-dpye)_2_PMo_12_@CPE	a	no	acidic	1.27 × 10^−7^ M	NR	Liu et al. (2021)
	H [Cu_2_(4-Hdpye)_2_PMo_12_@CPE	a	no		1.71 × 10^−7^ M	NR	
	{P_4_Mo_6_}/Cu/Mn/BBTZ@GCE	r	lake water	pH 0	1.59 nM	10 h	Niu et al. (2021)
	{P_4_Mo_6_}/Cu/Mn/BBTZ@GCE	r		1–5	<15 nM		
	{P4Mo_6_}/Na/Mn/BBTZ@GCE	r		0	2.91 nM	10 h	
	{P_4_Mo_6_}/Na/Mn/BBTZ@GCE	r	lake water	1–5	<24 nM		
	Cu_2_(OH) (Ptep)_2_Mo_8_@CPE	—	no	acidic	1.34 × 10^−4^ M	NR	Ying et al. (2021)
	{Cu^I^_5_ [4-atrz]_6_}^5+^-PMo_12_@GCE	a	no	acidic	5.4 × 10^−6^ M	1,000 cycles	Yang et al. (2021)
	{Cu^I^_5_ [4-atrz]_6_}^5+^-PW_12_@GCE	a	no		5.4 × 10^−6^ M	1,000 cycles	
	{Cu^I^_5_ [4-atrz]_6_}^5+^-SiW_12_@GCE	a	no		4.2 × 10^−6^ M	1,000 cycles	
Cd^2+^ and Pd^2+^	PW_12_/Cys@Au	a	industrial wastewater	acidic	9.0 nM and 4.0 nM	1 month	Dianat et al. (2019)

Abbreviations as reported by the authors. 4-atrz, 4- amino-triazole; Au, gold, BBTZ, 1,4-bis(1,2,4-triazol-1-ylmethyl) benzene; bpp, 1,3-bi(4-pyridyl)propane; btmc, 1,4-bis(1,2,4-triazol-1-methyl)cyclohexane; CPE, carbon paste electrode; ctcm, C-(4-[1,2,4]Triazol-4-ylmethylcyclohexyl)-methylamine; cys, cysteine; dm_4_bt, 2,2′-dimethyl-4, 4′-bithiazole; dpye, N,N′-bis (4-pyrimidinecarboxamido)-1,2-ethane; GCE, glassy carbon electrode; mct, 4-(4-Methyl-cyclohexylmethyl)-4H-[1,2,4]triazole; NR, not reported; Ptep, 1-[2-(3-pyridin-4-yl-[1,2,4]triazol-4-yl)-ethyl]-piperazine.

POM archetype structure according to the legend of Figure 2: a) Keggin and including r) hourglass type and -) unspecified type.

Cadmium (Cd) and lead (Pb) are used extensively in industry and are carcinogenic agents. Their accumulation in the human body can cause serious harm to internal organs such as lungs, kidneys, liver, bones, and central nervous system. Dianat *et al.* (Dianat et al., 2019) reported a novel sensitive L-cysteine Keggin tungstophosphate-modified polycrystalline gold electrode, listed in Table 6 as PW_12_/Cys@Au, developed for the electrochemical detection of Cd^2+^ and Pb^2+^ in trace amounts. The (Cys)PW_12_ hybrid compound was selected to fabricate inorganic self-assembled monolayers (SAMs) on the Au surface, which benefited from high stability due to the strong Au–S interaction. The modified electrode showed a wide linear range (0.01–0.2 μM) for both analytes, excellent reproducibility, high sensitivity, and reasonable detection limit (9.0 and 4.0 nM for Cd^2+^ and Pb^2+^, respectively), comparable to the other electrochemical techniques or modified gold electrodes (Dianat et al., 2019). The PW_12_/Cys@Au sensor was employed for Cd^2+^ or Pb^2+^ determination in industrial wastewater samples.

### 2.2 POM-Based Optical Sensors

Optical sensors are a broad class of devices detecting light and producing an electrical output. The principle of an optical sensor is based on shifts in the characteristic optical signal of an optical platform, resulting from interactions with analyte molecules which are used for quantitative or qualitative measurements. Most optical sensors are based on light absorption. Reflectance measurements may be made in opaque mediums that interact with the analyte giving rise to a colour change, while scattering is the phenomenon observed when the direction and or frequency of light is changed upon interaction. Surface-enhanced Raman scattering and luminescence, including fluorescence and phosphorescence, are good scattering examples. Fluorescence deserves special emphasis due to its superior selectivity and sensitivity compared to the more common absorption phenomena. Chemiluminescence allows detecting an analyte after a chemical reaction yielding an electronically excited species that emit when returning to the ground state. Surface plasmon resonance is based on the increase of the intensity of the evanescent wave by the collective oscillation of the free-electron plasma at an insulator/metal surface after the adsorption of the analyte. Often, the angle of incidence of light is changed, and the intensity of reflected light is being measured while molecules are attaching the chemically modified surface.

#### 2.2.1 POM-Based Absorption Sensors

POMs emerge as great promising species for absorption spectrophotometry, as accepting electrons gives rise to coloured mixed-valence state species while retaining their structural integrity. Table 7 lists the reported POMs-based absorption optical sensors, detailing the POM or the POM-hybrid composite, the POM archetype, the chromogenic substrate or reagents used, the working pH, the detection limit of the sensor, the sensor stability, and its application to real samples (more details can be found in Supplementary Table S7 in Supplementary Material).

**TABLE 7:** POM-based absorption optical sensors.

Targets	POM or POM hybrid material	POM archetype	Matrix	Chromogenic substrates/reagents	pH	Limit of detection	Stability studies	References
H_2_O_2_ and Glucose	PW_12_	a	no	TMB	3.0	NR	NR	Wang et al. (2012)
				GOx	7.0 and 3.0	NR	NR	
	SiW_12_	a	human blood	TMB	4.0	0.4 μM	NR	Liu et al. (2012)
				GOx	7.0 and 4.0	0.5 μM	NR	
H_2_O_2_ and Citric acid	Ni_4_(Trz)_6_/SiW_12_/PDDA-rGO	a	orange juice	TMB	2.5	0.49 μM	NR	Tong et al. (2020)
				H_2_O_2_, TMB	2.5	2.07 μM	5 runs	
H_2_O_2_ and Sarcosine	FA-PMo_4_V_8_	a	urine	TMB	4.0	0.012 μM	NR	Mbage et al. (2020)
					7.3 and 4.0	0.311 μM	NR	
H_2_O_2_	PW_12_/GO/FF	a	no	TMB	3	0.11 μM	10 batches	Ma et al. (2015)
NH_3_	Lcys/SiW_12_	a	no		> 5.2	NR	NR	Shen et al. (2012)
Hg^2+^	MLPOM^a^	d	industrial sewage	methanol		0.05 μM	NR	Chen et al. (2015b)
cancer cells	FA-PV_n_Mo_12-n_	a	3 types of cancer cells	TMB	7	NR	NR	Ji et al. (2015b)
UV light	PW_12_/SPS/PP	a		gly, ethanol		NR	NR	Lawrie et al. (2015)
	PMo_12_/LA	a				NR	8 weeks	Zou et al. (2018)
Dopamine and Ractopamine	SiW_9_Co_3_	a	no	H_2_O_2_		5.38 × 10^−6^ M and 7.94 × 10^−5^ M	NR	Duan et al. (2018)
Formaldehyde	PMo_10_V_2_/PVC/NPOE	a	commercial milk			0.2 mg L^−1^	8 days	Veríssimo et al. (2020a)
Dimethoate	PW_12_/Myr	a	lake water and juice			0.9 ng/ml	NR	Qi et al. (2020)
ZnCl_2_.2H_2_O	imi-SiMo_12_	a	no			0.15 μM	NR	Sabarinathan et al. (2021)
Glutathione	Mo-based POM/CR	—	mice		7.4	0.51 mM	48 h	Tang et al. (2019)

Abbreviations as reported by the authors. CR: croconaine; FA, folate acid; FF, diphenylalanine; Gly, glycerol; GO, graphene oxide; GOx, glucose oxidase; imi, imidazole; Lcys, L-cysteine; LA, lactic acid; Myr, myristoylcholine; NPOE, 2-nitrophenyl octyl ether; NR, not reported; PDDA, polydiallyldimethylammonium chloride; PP, polypropylene film; PVC, polyvinyl chloride; rGO, reduced graphene oxide; SPS, sulphonated polystyrene; TMB, 3,3′,5,5′-tetramethylbenzidine; Trz, 1,2,4-triazole.

a (n-Bu_4_N)_2_ [Mo_5_NaO_13_(OCH_3_)_4_(NO)].

POM archetype structure according to the legend of Figure 2: a) Keggin, d) Lindqvist and -) unspecified type.

“Heteropoly blues” are POMs of early transition metals [Mo(VI), W(VI) and V(V)] that exhibit a characteristic deep-blue colour after their reduction and have been reported for naked-eye colorimetric sensing. An example was the UV dosimeter indicator for solar water disinfection systems based on a Keggin [PW_12_O_40_]^3-^ in the presence of a sacrificial electron donor, such as glycerol, that allowed the identification of the point at which microbiologically contaminated water was solar-disinfected. It had advantages over other reported methods, such as the POM-based indicator’s ability to recover colour overnight and its reusability (Lawrie et al., 2015). Another example was the reduction of the Keggin phosphomolybdic acid [H_3_ (PMo_12_O_40_)] by lactic acid (LA) in the presence of different UV radiations. Reducing PMo_12_ to varying extents allowed the development of a skin-specific personalized UV dosimeter for spectrally selective colorimetric differentiation of UVA, UVB, and UVC by the naked eye (Zou et al., 2018). In addition, L-cysteine-doped tungstosilicate (represented in Table 7 as Lcys-SiW_12_) microtubes have been used to detect ammoniac gas by a colour change from light purple to dark blue (Shen et al., 2012). The result was confirmed by the new absorption bands that appear at 500 and 750 nm, attributed to the *d*–*d* transition and the W(V)-W(VI) intervalence-charge transfer that occurred in the doped tungstosilicate microtubes. Plus, by doping the heteropolyoxometalate with the amino acid L-cysteine (C_3_H_7_NO_2_S) containing sulphydryl groups, which are essential in biological processes, the biocompatibility of the POM microtubes was improved.

“Heteropoly blues” have also been reported as inorganic building blocks for fabricating organic-inorganic hybrids to mimic peroxidase, followed by colour sensing. Peroxidase is an enzyme that can catalyse the transfer of two electrons from a substrate to hydrogen peroxide to generate water and an oxidized substrate, and it is widely used in biochemistry applications. The peroxidase-like activity of POMs has been reported to catalyse 3,3,5,5-tetramethylbenzidine (TMB) to its oxidized form, which has a blue colour and could be detected using UV–vis spectroscopy or be seen with the naked eye. Based on H_2_O_2_ detection in the TMB system, biomolecules that could generate H_2_O_2_ from their reaction with oxidases, such as glucose (Wang et al., 2012; Liu et al., 2012), dopamine, and ractopamine (Duan et al., 2018), had been indirectly detected through colorimetric assays, as listed in Table 7. In addition, by combining the synergetic effects of the peroxide-like activity of different phosphovanado-molybdate PV_n_Mo_12−n_O_40_^(3+n)-^ (*n* = 1–3) and folic acid (FA), Ji et al. (2015b) developed folate-functionalized hybrids (listed as FA-PV_n_Mo_12-n_ in Table 7), successfully used as an indicator for colorimetric immunoassay of the cancer cells, where the FA enhanced the biocompatibility and improved the target to tumour cells of FA-PMoVn hybrids. The authors also demonstrated that without the synergistic effect of FA, the phosphovanado-molybdates could not target the tumour cells for detection. Later, this work led to the development of a simple FA-PMo_4_V_8_ system that showed excellent peroxidase-like activity, which was employed to colorimetric detection of sarcosine, a possible biomarker in urine and blood that indicates the malignancy of prostate cancer cells (Mbage et al., 2020). Also, the combination of peroxide-like activity of POMs with metal-organic frameworks (POMOFs) has been reported for bioenzyme free colorimetric sensing. The colorimetric sensor was conceived based on a POMOF, the [Ni_4_(Trz)_6_(H_2_O)_2_][SiW_12_O_40_].4H_2_O (Trz:1,2,4-triazole), and polydiallyldimethylammonium (PDDA) chloride functionalized reduced graphene oxide (PDDA-rGO). In the nanocomposite, listed as Ni4(Trz)/SiW_12_/PDDA-rGO (Tong et al., 2020) in Table 7, PDDA acted as a bridging agent to loading rGO nanosheet on the surface of Keggin [SiW_12_O_40_]^4-^ which offered excellent catalytic activities under extreme conditions (pH value 2.5), due to the nature and synergies from POMs, MOFs, and PDDA-rGOs. This bi-functional nanocomposite allowed the successful establishment of a platform for colorimetric sensing of H_2_O_2_ and citric acid (CA), with higher sensitivity (1–60 μM), fast response (10 min), and lower detection limit (2.07 μM) to CA than all other materials reported by the authors (Tong et al., 2020).

An exceptional ratiometric photoacoustic imaging (PAI) nanoprobe for glutathione (GSH), which plays important roles in a variety of diseases and cellular functions, was successfully achieved by Tang et al. (2019) through the self-assembly of croconaine (CR) dye and molybdenum-based polyoxometalate cluster into uniform nanoparticles, represented in Table 7 as Mo-based POM/CR. The authors discovered that the CR dye could be reduced specifically by GSH, showing a distinct GSH concentration-dependent decrease in the absorbance at 700 nm. In contrast, the Mo-based POM clusters were reduced by the GSH, increasing their absorbance at 866 nm due to the GSH-activated Mo(VI) to Mo(V) conversion. Thus, the photoacoustic (PA) signal ratio of CR-POM at these two wavelengths (PA866/PA700) was much higher than most existing ratiometric PAI probes. Furthermore, the relatively low LOD (0.51 mM) and the linear range up to 14 mM, revealed the capability of Mo-based POM/CR for GSH quantification, which covered exactly the range of the GSH concentration *in vivo* (0.5–10 mM), which was highly competent for noninvasive quantification of GSH *in vivo*.

However, not only molecules with biological relevance have been successfully detected by POM-based colorimetric sensors. High toxic metals and food contaminants have also deserved the researcher’s attention. A metal-oxo cluster, (n-Bu_4_N)_2_ [Mo_5_NaO_13_(OCH_3_)_4_(NO)], organically-derivatized from a monolacunary Lindqvist (ML)-type polyoxomolybdate, listed as MLPOM in Table 7, was reported to specifically react with Hg^2+^ in methanol, displaying a colour change from purple to brown within seconds, after mixing, with a detection limit of 0.05 μM, which was below the guideline value of Hg^2+^ for contaminated sewage from mercury industries (Chen et al., 2015b). By comparing the structure of polyoxomolybdate, before and after reaction, the colour change was revealed to be caused by the structural transformation of MLPOM accelerated by Hg^2+^. Additionally, the developed POM-based colorimetric sensor showed a remarkably high selectivity over other environmentally relevant metal ions, such as Fe^2+^, Fe^3+^, Cr^3+^, Zn^2+^, Pb^2+^, Ni^2+^, Ag^+^, Al^3+^, Mn^2+^, Cd^2+^, Ca^2+^, Co^2+^, and Cu^2+^.

Furthermore, a Keggin POM-based optical sensor for ZnCl_2_.2H_2_O, working both in solution as in solid-state, based on [Himi]_4_ [SiMo_12_O_40_] (imi = imidazole), was recently reported by Sabarinathan et al. (2021), where the Molybdenum blue (reduced Mo^5+^) appeared only in the presence of ZnCl_2_.2H_2_O which confirmed the involvement of water molecules in the reduction mechanism. This POM-based sensor, listed as imi-SiMo_12_ in Table 7, achieved a much lower LOD (0.15 μM) than results published in the literature and a superior selectivity over metal chloride solutions of 15 different metals and other salts of Zn^2+^. Besides, the imidazole ring possesses potent antimicrobial activity against multiple pathogenic microbes, and in particular against *Staphylococcus aureus*.

An optical fibre sensor, based on a cladding stripped tip coated with a Keggin-type [(C_4_H_9_)_4_N]_4_H [PMo_10_V_2_O_40_], specially designed to be insoluble in water, incorporated into a plasticized polyvinylchloride (PVC) membrane containing *o*-nitrophenyl octyl ether (NPOE), was reported for formaldehyde detection in milk (Veríssimo et al., 2020a). The UV–Vis spectrum of the POM-coating membrane, listed in Table 7 as PMo_12_V_2_/PVC/NPOE, changed with formaldehyde. The LOD for formaldehyde determined with the optical sensor was 0.2 mg L^−1^, similar to the value of the conventional acetylacetone spectrophotometric method, though the limit of quantification (LOQ) was slightly lower for the spectrophotometric method, 0.5 mg L^−1^ and 0.6 mg L^−1^, respectively. In addition, the described methodology has the advantage of not requiring a heating step, one of the disadvantages of the conventional acetylacetone spectrophotometric method, which prevents its use in the field.

#### 2.2.2 POM-Based Fluorescence Sensors

Fluorescence readouts are particularly interesting for very sensitive sensing applications. The changes in the intensity of light emitted at longer wavelengths than the excitation can be quantitatively related to the concentration of the analyte. Rare-earth (RE)-based materials are often used to manufacture fluorescence (FL) chemosensors because RE ions with rich electron energy levels show outstanding luminous properties under light excitation. Therefore, the combination of RE and POMs, that provide numberless oxo-active sites to capture RE ions, appears as the ideal combination.

##### 2.2.2.1 Ln-POMs-Based Fluorescence Sensors

Table 8 summarizes the Lanthanide (Ln)-based materials (Ln-POMs-based fluorescence sensors) reported so far in the literature. Table 8 includes the POM archetype, the detection limit, the operation mode of the sensors and the matrix where sensors were tested (more details can be found in Supplementary Table S8 in Supplementary Material). Among Ln-POMs reported probes in Table 8, the Lindqvist europium decatungstate Na_9_ [EuW_10_O_36_].32H_2_O (EuW_10_) was the most prevalent due to Eu fascinating property of changing colour and fluorescence dependence on its valency and coordination environment. However, as pure EuW_10_ exhibited only weak photoluminescence in water, due to luminescence quenching of water molecules, quenching in solution must be prevented by self-assembly of Eu-POM with organic materials, such as polymers, metal-organic frameworks (MOFs) and surfactants.

**TABLE 8:** Lanthanides POM-based fluorescence optical sensors.

Targets	POM or POM hybrid material	POM archetype	Matrix	Detection limit	Operation mode	References
Zn^2+^ and UV light	EuW_10_/PyC_10_C_12_N	d	no	NR	luminescent logic gate with dual output	Zhang et al. (2006)
solar UV-light	EuW_10_/PVP/PEI/AV^2+^	d	no	NR	portable solar UV-light sensor	Liu et al. (2017)
HCl and NH_3_	EuW_10_/agarose	d	no	NR	luminescence sharply decreases with HCl gas and recover upon subsequently exposing the films to NH_3_ gas	Wang et al. (2010)
	TbW_10_/agarose	d	no	0.2731 mM	luminescence sharply decreases with HCl gas and recover upon subsequently exposing the films to NH_3_ gas	Wang et al. (2019a)
Metanil Yellow,Allura red, Auramine O,Orange II	PrW_10_/CNO	d	no	3.83 nmol ml^−1^ 2.90 nmol ml^−1^ 4.73 nmol ml^−1^ 4.14 nmol ml^−1^		Dutta and Sarkar, (2016)
Fe^3+^ and amino-acids	EuW_10_/UiO-67	d	no	37 μΜ	luminescence intensity quenched by Fe^3+^ and enhanced by amino-acids	Salomon et al. (2018)
MnO_4_^−^and Cr^3+^	EuW_10_/[C_14_-2-C_14_im]Br_2_	d	no	1.70 μΜ and 0.926 mM	off-luminescence chemical sensor	Sun et al. (2019)
Cr^3+^ and Ca^2+^	EuPW_11_/PHBA	a	no	1.423 mM and 0.676 mM	luminescence intensity quenched by Cr^3+^, and enhanced by Ca^2+^	Wu et al. (2019)
Ascorbic acid and NO_2_^−^	EuSiMoW_10_	—	urine, spinach	0.53 μΜ (UV-Vis) and 4.67 μΜ (fluorescence) 1.16 mM (UV-Vis) and 5.39 mM (fluorescence)	reversible change of colour and luminescence	Fu et al. (2019)
Cu^2+^	EuMnMo_6_/PPCT	c	no	24 nM		Yuan et al. (2019)
Vitamin C and H_2_O_2_	TbP_2_Mo_18_	b	no	NR		Bin et al. (2019)
Ba^2+^	Eu-arsenotungstates/H_2_tpdc	—	no	1.19 × 10^−3^ mM	good recognition responses toward detecting the Ba^2+^ ion in the absence of Ca^2+^ or Sr^2+^ ions in aqueous system	Wang et al. (2020d)
Cu^2+^ and L-cysteine	EuSe_3_W_14_ ^a^	b	no	1.24 × 10^−3^ mM and 2.17 × 10^−4^ mM	turn-off/on	Zhang et al. (2020a)
	EuTeW_9_ ^b^	—	no	8.82 × 10^−6^ mM and 1.75 × 10^−4^ mM	turn-off/on	Zhang et al. (2020b)
Temperature	EuW_10_/Tb-TATB	d	no	NR		Viravaux et al. (2021)
Ag^+^ and cholyglycine	Eu_4_W_8_/EB-TFP	—	tap and river water	0.014 μg ml^−1^ and 0.024 μg ml^−1^	luminescence turn-on/off	Wang et al. (2021)

Abbreviations as reported by the authors. AV^2+^, N,N′-bis(δ-aminopropyl)-4,4′-bipyridine bromide hydrobromide; CNO, carbon nano-onion; EB, ethidium bromide; glu, D-gluconic acid; NR, not reported; PEI, polyethyleneimine; PHBA, *p*-hydroxybenzoic acid; PPCT, 4′ 2,2':6′,2″para-phenylcarboxyl-terpyridine; PVP, polyvinylpyrrolidone; PyC_10_C_12_N, *trans*-10-(4-(4′-pyridylvinylene)-phenyl)oxydecyldodecyldimethylammonium bromide; TATB, triazine-1, 3,5-tribenzoic acid; TFP, 1,3,5-triformylphloroglucinol; tpdc, 2,5- thiophenedicarboxylic acid; UiO-67, zirconium luminescent metal-organic framework.

a [H_2_N(CH_3_)2]_10_H_3_{SeO_4_Eu_5_(H_2_O)_8_ [Se_2_W_14_O_52_]_2_}·40H_2_O.

b K_14_H_10_ [Eu_4_(H_2_O)_4_W_6_(H_2_glu)_4_O_12_(B-α-TeW_9_O_33_)_4_]·60H_2_O.

POM archetype structure according to the legend of Figure 2: a) Keggin, b) Dawson, c) Anderson, d) Lindqvist and -) unspecified type.

Self-assembly of Eu-POM with polymers was reported by Wang et al. (2010). A highly transparent flexible self-supporting decatungsteuropate thin film, listed in Table 8 as EuW_10_/agarose, was fabricated by a facile hydrogel casting technique. The strong interactions between agarose and EuW_10_ by hydrogen bonds at the hydroxyl sites and the densely packed 3D network structure of agarose in the gel contributed to the homogenous distribution of EuW_10_ and to the good mechanical properties of the nanocomposite films. When excited with UV-light, the thin-films of EuW_10_/agarose displayed a strong red emission of Eu^3+^ that can be reversibly modulated, quenched by HCl gas, and recovered by NH_3_ gas, behaving as a luminescent switch. Also a TbW_10_/agarose composite thin film reported more recently by Bin et al. (Wang et al., 2019a), showed the same behaviour in the presence of HCl and NH_3_ gases, with the green luminescent thin-film sensor presenting a detection limit of 0.2731 mmol L^−1^ for HCl, showing that other Ln-POM (Dutta and Sarkar, 2016), besides Eu-POMs, could be used for sensing.

The self-assembly of Eu-POMs with 3D coordination networks (MOFs) also appears as a promising approach due to their crystalline nature, permanent porosity, chemical tunability, and robustness, offering an advantageous unique platform for the development of solid-state luminescent materials. Thus, POMs incorporated in the cavities of a metal-organic framework (POM/MOFs) have been used to prevent luminescence quenching. Salomon et al. (2018) reported the introduction of the luminescent EuW_10_ into the cavities of highly porous zirconium luminescent MOF UiO-67, combining dual-luminescent properties of EuW_10_/UiO-67, as listed in Table 8. The hybrid material proved to be a solid-state luminescent sensor for amino acids. Enhancement of the EuW_10_/UiO-67 luminescence is observed in the presence of amino acids globally following the increase of the amino-acid pKa. Due to the strong quenching effect of Fe^3+^ (K_SV_ 2667 M^−1^), the EuW_10_/UiO-67 proved to be also a reusable sensor for Fe^3+^ in an aqueous solution, with an estimated LOD of 37 µM. Recently, the same group reported the encapsulation of the EuW_10_ into a mesoporous MOF, a Tb-TATB, built of terbium tetranuclear units connected by TATB ligands (H_3_TATB = triazine-1,3,5-tribenzoic acid). The dual-luminescent EuW_10_/Tb-TATB composite (as listed in Table 8) behaved like a highly sensitive luminescent thermometer in the physiological domain and gave rise to a new family of hybrid dual-emitting LnPOM/LnMOF materials (Viravaux et al., 2021).

An example of surfactant-encapsulated polyoxometalates was reported by Hui et al. (Zhang et al., 2006), where the luminescent polyoxometaloeuropate EuW_10_ was connected through electrostatic interaction with the multi-functional surfactant, *trans*-10-[4-(4′-pyridylvinylene)-phenyl] oxydecyldodecyldimethyl-ammonium bromide (PyC_10_C_12_N). The combined composite, listed as EuW_10_/PyC_10_C_12_N in Table 8, worked as a luminescent logic gate with dual output, operated by light and zinc ion as inputs. Another example was reported by Panpan et al. (Sun et al., 2019), where the polyoxometaloeuropate was used to develop a sensitive, selective off-luminescence chemical sensor, listed as EuW_10_/[C_14_-2-C_14_im] Br_2_ in Table 8, for the label-free detection of Cr^3+^ and MnO_4_^−^ in aqueous solution, with low detection limits of 0.926 and 1.70 μM, respectively, and a wide pH application range. The introduction of [C_14_-2-C_14_im]Br_2_ did increase the luminescence effect, and the strongest luminescence was observed for EuW_10_/[C_14_-2-C_14_im]Br_2_, which was 32 times that of pure EuW10.

Although the luminescent EuW_10_ dominate in the Ln-POM based fluorescence optical sensors group, other luminescent Eu-POMs have been reported (Table 8). Two systems, a penta-Eu^III^ sandwiched Dawson-type selenotungstate (Zhang et al., 2020a) [H_2_N(CH_3_)2]_10_H_3_{SeO_4_Eu_5_(H_2_O)_8_ [Se_2_W_14_O_52_]_2_}·40H_2_O, represented in Table 8 as EuSe_3_W_14_, and a polyhydroxycarboxylic acid ligand bridged multi-Eu^III^-incorporating tellurotungstate (Zhang et al., 2020b) K_14_H_10_ [Eu_4_(H_2_O)_4_W_6_(H_2_glu)_4_O_12_(B-*α*-TeW_9_O_33_)_4_]·60H_2_O (H_6_glu = D-gluconic acid), represented in Table 8 as EuTeW_9_, were reported as sensors to detect Cu^2+^ ions in aqueous solution. Both systems exhibited high fluorescence signals and good selectivity for detecting Cu^2+^ ions in an aqueous solution (Zhang et al., 2020a; Zhang et al., 2020b). The Eu-tellurotungstate showed the best performance due to the hexagonal packing of the tetrameric polyoxoanions, providing excellent porous channels, which greatly increased the specific surface area of the whole framework and fluorescence sensing. This is the most sensitive POM-based fluorescence sensor for detecting Cu^2+^ ions in an aqueous solution reported so far (LOD 8.82 × 10^−6^ mM). Furthermore, the same sensors could be used in Cu^2+−^quenching systems. These “off-on” fluorescence sensors were used to detect Cysteine (Cys) in an aqueous solution with similar LODs (2.17 × 10^−4^ and 1.75 × 10^−4^ mM, with EuSe_3_W_14_ and EuTeW_9_, respectively).

##### 2.2.2.2 Other Luminescent POMs-Based Sensors

Besides Ln-substituted POMs, other luminescent hybrid POMs have been reported by binding chromophore species to POMs, which are summarized in Table 9, along with the POM archetype, their target analyte and the matrix where they were tested, the operation mode of the sensor and the detection limit (LOD) (more details can be found in Supplementary Table S9 in Supplementary Material).

**TABLE 9:** POM-based fluorescence optical sensors.

Target	POM or POM hybrid material	POM archetype	Matrix	Substrates	Operation mode/Limit of detection	References
Cu^2+^ and Pb^2+^	SiW_10_/dansyl	a	no		Fluorescence quenched by Cu^2+^ and enhanced by Pb^2+^	Carraro et al. (2012)
pH	Mo_8_/norfloxacine	j	no		Acid-base switch	Liu et al. (2015)
VOCs	Mo_8_/[Ir^III^(PPy)_2_ (bpy)]^+^	—	no		Depending on VOC polarity	Bolle et al. (2016)
Picric acid and Pd^2+^	V_10_O_28_/Cu-pyno-NEt	e	no		0.18 ppb and 0.80 ppb, for picric acid and Pd^2+^, respectively	Raizada et al. (2017)
H_2_O_2_	SiW_9_	a	water	BA TH HPPA	6.7 × 10^−9^ M 2.2 × 10^−7^ M 9.6 × 10^−6^ M	Tian et al. (2019)
Hg^2+^	Zn-dbt/P_2_W_18_ Cd-dbt/P_2_W_18_ Cd-dbt-Cl/PW_12_ Cd-dbt/SiW_12_	b	no		NR	Ying et al. (2019)
Hg^2+^	Ag- Py_2_TTz/PMo_12_	a	no		NR	Mou et al. (2020)
Hg^2+^	Cu-dm4bt/PMo_12_	a	no		NR	Wang et al. (2020b)
Hg^2+^	Zn-MET/CrMo_6_ Cu-MET/CrMo_6_	j j	no		For both POM composites, the fluorescence is quenched to a large extent by Hg^2+^	Zhang et al. (2021a)
Dopamine	FeMo_6_/rGO	c	Human serum and dopamine hydrochloride injection	OPD ABTs TMB	two consecutive “turn on” fluorescence 0.0112 μM	Li et al. (2021)

Abbreviations as reported by the authors. ABTs, 2,2′-azino-bis(3-ethylbenzthiazoline-6-sulfonic acid); BA, benzoic acid; dm4bt, 2,2′-dimethyl-4, 4′-bithiazole; HPPA, 3-(4-hydroxyphenyl) propionic acid; MET, 4-(3-imidazol-1-yl-ethyl)-4H-[1,3,4]triazole; NEt, Triethylamine; NR, not reported; OPD, *o*-phenylenediamine, PPy, polypyrrole; Py2TTz, 2,5-bis(4-pyridyl)thiazolo[5,4-*d*]thiazole; Pyno, 4-picoline N-oxide; rGO, reduced graphene oxide; TH, thiamine, TMB, 3,3′,5,5′-tetramethylbenzidine.

POM archetype structure according to the legend of Figure 2: a) Keggin, b) Dawson, c) Anderson, e) decavanadate, j) *γ*-octamolybdate, and -) unspecified type.

Carraro et al. (2012) reported a bis-lacunary Keggin polyoxotungstate [*γ*-SiW_10_O_36_]^8–^ as a molecular nanosurface where the dansyl chromophore was anchored with a tweezer-type arrangement, which acted as a selective fluorescence sensor for Cu^2+^ and Pb^2+^ ions, in quenching and enhancing mode, respectively. Hong et al. (Liu et al., 2015) reported the use of Norfloxacin, a known fluorescence medicine, to produce a Norfloxacin-derivative functionalized octamolybdate, (dNF)_2_ [*γ*-Mo_8_O_26_(dNF)_2_].10H_2_O, where dNF stands for decarboxylated norfloxacin. The combination, listed as SiW_10_/dansyl in Table 9, showed to be an acid-base switch system, as both the addition of acid or base modulated its fluorescence. Another simple ionic association of a photoluminescent compound, the [Ir^III^(ppy)_2_ (bpy)]^+^ complex incorporating 2-phenyl- pyridine (ppy) and 2,20-bipyridine diimine (bpy), with an octamolybdate result in a strong modulation of its emission wavelength in the solid-state, varying from green to yellow, orange, orange-red, and red, by changing the nature of the POM and the design of the frameworks. The resulting hybrid materials, listed as Mo_8_/[Ir^III^(ppy)_2_ (bpy)]^+^ in Table 9, turned to be an efficient selective chemosensor for VOC detection (Bolle et al., 2016).

Furthermore, Tian et al. (Tian et al., 2019) reported the synergetic combination of organic species such as benzoic acid (BA), thiamine (TH), and 3-(4-hydroxyphenyl)propionic acid (HPPA), with the Keggin-type polyoxotungstate intrinsic peroxidase Na_10_ [*α*-SiW_9_O_34_] decomposing H_2_O_2_ into ▪OH radicals, which converted weakly fluorescent substrates to strongly fluorescent substrates, under basic pH conditions. Recently, S-/N-containing ligands, such as 2,2′-dimethyl-4,4′-bithiazole (Ying et al., 2019; Wang et al., 2020b) and 2,5-bis(4-pyridyl)thiazolo [5,4-*d*] thiazole (Mou et al., 2020), and N-containing ligands, such as 2,2′-bipyridyl (bpy) and 4-(3-imidazol-1-yl-ethyl)-4H-[1,3,4]triazole (MET) (Zhang et al., 2021a) were used to modify a series of different archetype POM compounds, in order to build fluorescence sensors for Hg^2+^. All compounds showed selective response to Hg^2+^, explained by the preferred interaction between the soft acidic Hg^2+^ ions and sulphur (soft base). In addition, all hybrid POMs proved to be multi-functional materials, showing not only photocatalytic activity for degradation of dyes (Ying et al., 2019; Wang et al., 2020b; Mou et al., 2020; Zhang et al., 2021a) but also redox properties, which makes them probe to act as electrochemical sensors for NO_2_^−^ (Ying et al., 2019; Mou et al., 2020; Wang et al., 2020b; Zhang et al., 2021a), for H_2_O_2_ (Zhang et al., 2021), and Cr(VI) (Wang et al., 2020b).

Hybrid metal-POMs showing fluorescence properties and applied to sensing have also been reported. The terminal and bridging oxygen atoms on the surface of POMs not only can act as versatile proton acceptors and donors but can also coordinate with other metal ions. Mukul et al. (Raizada et al., 2017) synthesized a decavanadate hybrid material with 4-picoline N-oxide (Pyno) and triethylamine (NEt_3_), the {Cu(Pyno)_4_}{NEt_3_H}_2_ [H_2_V_10_O_28_] cluster, denoted in Table 9 as V_10_O_28_-Cu-pyno-NEt, in which the metal ion linkers belong to distinct coordination complexes with peripheral organic ligands. This water-soluble inorganic-hybrid compound was investigated as the first aqueous-phase sensor for picric acid and Pd^2+^, with a low detection limit of 0.18 and 0.80 ppb, respectively, within WHO/US EPA prescribed limit for palladium. Another example was recently reported by Qian et al. (Li et al., 2021), where the Anderson type (NH_4_)_3_ [H_6_Fe(III)Mo_6_O_24_] (FeMo_6_), working as an oxidase-mimic nanoenzyme, exhibited the ability to catalytic oxidase of *o*-phenylenediamine (OPD), 2,2′-azino-bis(3-ethylbenzthiazoline-6-sulfonic acid) (ABTs), and 3,3′,5,5′-tetramethylbenzidine (TMB). The proposed sensor based on two consecutive “turn on” fluorescence was developed for DA by employing the FeMo_6_-OPD system, and the linear range was from 1 to 100 μM with the detection limit 0.0227 μM. In addition, by loading the FeMo_6_-OPD system with 10% of reduced graphene oxide (rGO), listed in Table 9 as FeMo_6_/rGO, the authors increased the oxidase-mimic activity of FeMo_6_, with an enhancement of the detection limit to 0.012 μM.

#### 2.2.3 POM-Based Surface-Enhanced Raman Scattering Sensors

While several POM-based fluorescence sensors and POM-based absorbance optical sensors for UV-Vis spectroscopy could be found in the literature, only one paper reported the use of a POM in a reduced graphene oxide (rGO)/Ag film as a surface-enhanced Raman scattering probe for the selective detection of trace formaldehyde in the presence of other aldehydes (Zhang et al., 2017a). The use of the Keggin H_3_PW_12_O_40_ (PW_12_) as a photoreduction agent helped to improve the reduction degree of the GO. Compared with surface-enhanced Raman scattering probes prepared with the PW_12_/rGO film and the PW_12_/Ag film, the PW_12_/rGO/Ag film displayed a higher sensitivity and the detection limit for formaldehyde reached 1.0 × 10^−8^ M.

### 2.3 POM-Based Mass Sensors

Piezoelectric crystals, like quartz, vibrate with the application of an oscillating electric potential. The acoustic wave propagates on the bulk of the crystal, but a change of mass at the surface of the crystal changes the frequency of oscillation. Other mass-sensitive sensors are based on the launch of a surface acoustic wave from a transmitter consisting of interdigitated electrodes that travel on the surface of the piezoelectric material to another interdigitated set of electrodes, the receiver. These surface acoustic wave devices operate at much higher frequencies but are not as popular as the bulk acoustic wave devices (BAW), also known as quartz crystal microbalances (QCM) when used as gravimetric sensors.

QCM is a powerful technique to study the dynamics of adsorption processes. It was used to monitor organic-inorganic hybrid films growth by recording the quartz crystal’s frequency decrease during each adsorption cycle. QCM sensors were used to study the adsorption of Keggin phosphotungstic acid POM onto a copolymer-coated QCM as a function of time at several pH conditions (Raj et al., 2015). Also, the organic-inorganic hybrid polyoxometalate (NBu_4_)_3_ [PW_11_O_39_{(SiC_6_H_4_NH_2_)_2_O}], carrying two amine functions, allowed the construction of an ordered array of amine groups on the sensor surface for benzo [a]pirene detection. The ordered surface enabled better accessibility of the immobilized molecules compared with a reference layer built from an amine-terminated self-assembled monolayer on gold and, consequently, a significant increase in biosensor sensitivity (Mercier et al., 2015). However, papers that use a QCM coated with a POM (POM@QCM), where the POM is the recognition element, are very scarce. Veríssimo *et al.* (Veríssimo et al., 2017) reported the use of an acoustic wave sensor coated with a sensitive layer of a Keggin-type decamolybdodivanadophosphate (*α*-[PMo_10_V_2_O_40_]^5-^), that has been previously reported as an effective redox catalyst for volatile organic compound (VOCs) oxidation (Gamelas et al., 2012). This PMo_10_V_2_@QCM sensor was used to detect 5-hydroxymethylfurfural (HMF), a potentially mutagenic, carcinogenic and genotoxic compound, an excellent indicator of honey ageing, poor storage conditions, excessive heat-treatment, or possible adulteration with other sugars or syrups. Sensor lifetime was at least 6 weeks without sensitivity loss, and the quantification limit was well below the legislation threshold of 11.4 μg g^−1^ for HMF in honey. Another POM@QCM sensor was also published by Veríssimo et al. (2018), where the sensitive coating was a POM salt specially tailored to be insoluble in water. The Keggin-type polyoxotungstate, with tetrabutylammonium (TBA) as counter-cation, [(C_4_H_9_)_4_N]_4_ [PW_11_Mn^III^(H_2_O)O_39_], was used as the sensitive membrane of the piezoelectric quartz crystal for acetaldehyde quantification in cider. Results were not statistically different from those obtained with Gas Chromatography-Flame ionization Detection (GC-FID), and LOD and LOQ were similar. The sensor was stable for at least 8 weeks.

## 3 Summary and Outlook

**FIGURE 5:**
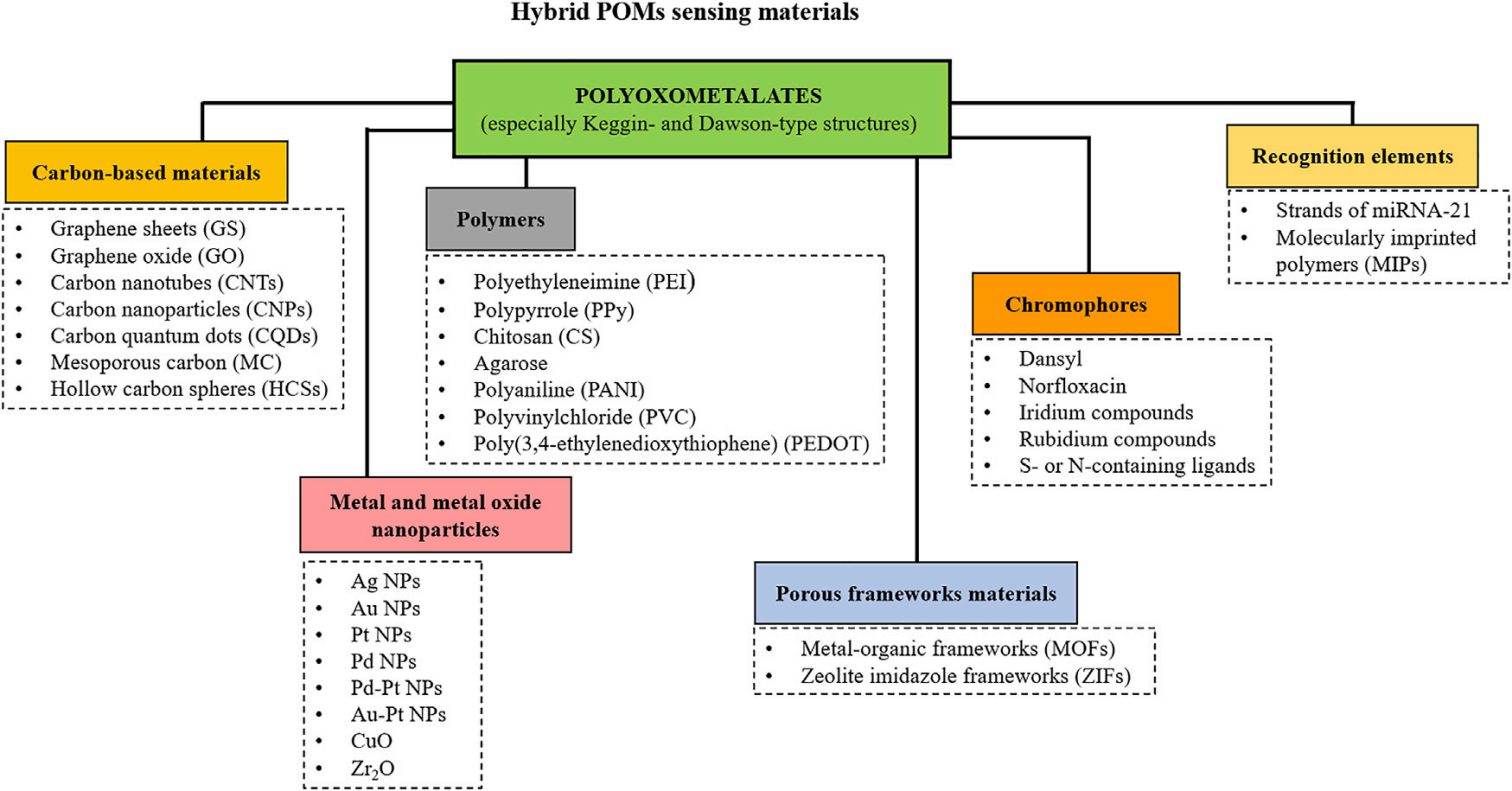
Summary of the most reported materials used in polyoxometalate functionalized sensors.

Looking at the vast list of applications summarized in this review, a first conclusion emerges that POM-based composites used for sensing applications are mainly based on Keggin [(XM_12_O_40_)^n−^]- and Wells-Dawson [(X_2_M_18_O_62_)^n−^]-type structures, with Keggin-type being responsible for more than 60% of the listed POMs in Table 1, Table 2, Table 3, Table 4, Table 5, Table 6, Table 7, Table 8, and Table 9. Listed POMs were combined with the most diverse materials, such as metals, polymers, carbon-based materials, and porous framework materials, such as metal-organic frameworks, zeolites and molecular imprinted polymers. Figure 5 schematically summarizes POM hybrid structures used for sensing. These POMs’ modifications enhanced redox, conductive and catalytic properties, included chromophores to enhance optical signals, changed material shape or built cavities with particular geometries and chemical functional groups. Besides, the careful choice of the counter-ion imparts the desired insolubility, preventing the leaching of the sensor sensitive layer.

POM-based electrochemical sensors provided low-cost and straightforward systems competing with expensive and sophisticated technologies, with detection limits for most applications as low as micromolar, with some special applications reaching pM and fM. The additions of POMs to solid electrodes is a result of their multielectron redox properties, giving rise to fast and sensitive responses. POM-hybrid materials used for CMEs were the most diverse. The highlight goes to those combined with nanocarbon materials or metal nanoparticles, which undoubtedly enhanced stability and improved electrochemical performance. Furthermore, the immobilization of specific DNA strands, or the combination with molecularly imprinted polymers, largely influenced the sensor’s sensitivity and selectivity.

POM-based optical sensors were reported for various targets, from metals to biomolecules. Concerning the POM-based absorption optical sensors, which were mainly Keggin-type structures, the standing out goes to the “heteropoly blues”, which acted as inorganic building blocks and were used to fabricate organic-inorganic hybrids to mimic peroxidase with success. Regarding the POM-based fluorescence sensors, those with Eu-POM, have recently been in the spotlight, due to their ability to act as efficient photoswitches or very selective fluorescent probes. Anyway, it is worth highlighting the bifunctionality of most optical sensors based on POM, an asset that allows the sensing of multiple analytes.

POM-based mass sensors, although less explored, also have a say in the world of POM functionalized sensors. Besides sensing for quantitative analysis, these piezoelectric sensors could be used for adsorption studies with great effectiveness. Furthermore, one of the advantages of QCM detection lays on their applicability both in gaseous and liquid media, which may spark new interest in this line of research in the coming years.

This overview of the literature concerning functionalized POM sensors revealed that, in general, the analytical properties of the proposed sensors are significantly better than others previously reported, based on other types of compounds. The design of POM-hybrid materials conceived having in mind the final target, and considering the most appropriate transducer for each application, allowed the development of POM-based sensors with extremely low limits of detection (pM and fM) in line with more sophisticated and expensive analytical techniques. Still, researchers do not always address critical issues such as selectivity, nor do they validate sensors by applying them to real samples.

It is expected that, in the future, POM hybrid materials contribution to the sensing area will increase, with different organic-inorganic hybrid materials providing different coordination modes to construct more specific structures, which will potentially exhibit enhanced performances. Besides, it can be envisaged that POM-based sensors future trends will rely on multi-functional nanomaterials with multi-stimuli responsive materials, contributing to a new era of smart sensors. The possibility to interrogate a POM platform in multiple ways, combining different transducers is an interesting possibility, still waiting for the pioneers.
