# Supplementary material for: Polyoxometalate Functionalized Sensors: A Review
Source: Front Chem. 2022 Mar 8;10:840657. doi: 10.3389/fchem.2022.840657 (PMC8964365; doi:10.3389/fchem.2022.840657)
Supplement: Supplementary file 1 [file DataSheet1.docx]

Polyoxometalate functionalized sensors: A Review

Marta I. S. Veríssimo^1*^, Dmitry V. Evtuguin^2^, M. Teresa S. R. Gomes^1*^

^1^CESAM, Department of Chemistry, University of Aveiro, Aveiro, Portugal

^2^CICECO, Department of Chemistry, University of Aveiro, Aveiro, Portugal

*** Correspondence:**Corresponding authors
[mverissimo@ua.pt](mailto:mverissimo@ua.pt) (Marta I. S. Veríssimo); [mtgomes@ua.pt](mailto:mtgomes@ua.pt) (M. Teresa S. R. Gomes)

Table S1- POM-based electrochemical sensors for H_2_O_2_ detection.

| **Hybrid material@Electrode** | **POM**  **archetype** | **Matrix** | **pH** | **Linear**  **range** | **Limit of  detection** | **Sensitivity** | **Response**  **time** | **Stability**  **studies** | **References** |
| --- | --- | --- | --- | --- | --- | --- | --- | --- | --- |
| P_2_W_17_V/graphite/organoceramic@CPE | b | no | acidic | 0.1-20 μM | 4x10^-5^ M | 0.753 μA mM^-1^ | < 5s | 3 months | Wang et al. (2000) |
| PMo_12_@Pt | a | no | acidic | 2x10^-5^-3x10^-2^M | 7x10^-6^M | 3.6 μA mM^-1^ | NR | NR | Song et al. (2000) |
| Fe_4_POM^*^/poly(1,8 DAN)@GE | a | no | 2.5 | Up to 50 mM | 2 mM | 0.11 mA M^-1^ | < 1s | no | Turdean et al. (2002) |
| (H_6/5_bppy)_5-_P_2_W_18_@CPE | b | no | acidic | 2x10^-4^-2.2x10^-3^ M | 1.3x10^-5^ M | 1.591 μA mM^-1^ | < 4s | 1 month | Tian et al. (2007) |
| P_2_Mo_18_/OMC@GCE | b | no | acidic | 160 -44000 μM | 53.4 μM | NR | NR | NR | Zhou et al. (2007) |
| SWCNTs/SiMo_12_/[Cu(bpy_)2_]^2+^@GCE | a | no | 1 | 10 nM-18 μM | 1 nM | 5.5 nA nM^-1^ | NR | 30 days | SAlimi et al. (2009) |
| APS/PFeW_11_@CPE | a | no | 2 | 10-200 μM | 7.4 μM | 0.183 nA μM^-1^ | NR | NR | Hamidi et al. (2009) |
| VMo_12_/[BMIM][PF_6_]@CPE | a | no | 4 | 0.05-2 mM | 2.33 μM | 50.83 μA mM^-1^ | 10s | 2 weeks | Ji et al. (2009) |
| MWCNTs/[C_8_Py][PF_6_]/PMo_12_@GCE | a | no | 1 | 0.02-8 mM | 12 μM | 5.68 μA mM^-1^ | < 2s | 20 times a day  / 5 days | Hadhighi et al. (2010) |
| MPS/B/PFe_3_Mo_9_@Au | a | no | 6.2 | Up to 0.9 M | NR | 0.4 ± 0.01 mA M^-1^ | < 1min | NR | Turdean and Popescu, (2012) |
| K_5_[Ru(bpy)_3_]-PW_18_@GCE | b | no | 7 | 0.5 μM−90 mM | 0.5 μM | 0.78 μA mM^-1^ mm^-2^ | < 5s | 5 weeks | Ammam and Easton, (2012) |
| P_2_W_17_Fe/PdNPs@ITO | b | no | 2 | 1.5 μM−3.9 mM | 1 μM | 66.7 μA mM^-1^ | < 3.5s | 1 month | Zhu et al. (2013) |
| P_8_W_48_/chitosan/PEI@ITO | n | no | 5 | 25 μM−2.3 mM | 1.3 μM | 0.53 μA mM^-1^ | ~ 5s | 2 months | Kang et al. (2015) |
| AuNPs/PW_12_/OMC@GCE | a | Disinfectant solution | 7 | 1-20 μM | 0.36 μM | 72.44 μA mM^-1^ | 2s | two weeks | Zhang et al. (2015) |
| P_2_W_18_/CNTs/AuNPs@ITO | b | no | 2 | 1-98 μM | 52 nM | 596.1 μA mM^-1^ cm^-2^ | < 1s | 20 days | Guo et al (2015a) |
| PW_12_/PEI@ITO | a | no | 5 | 3.5x10^-3^-3.5x10^-2^ mg/mL | 8.4x10^-4^ mg/mL | NR | NR | NR | Xu et al. (2015) |
| PtNPs/PMo_12_/OMC@GCE | a | no | 7 | 5-5400 μM | 1.9 μM | 10.64 μA mM^-1^ | NR | 2 weeks | Li et al. (2016) |
| PMo_12_/PANI@Au | a | no | acidic | 0.1-20 mM | 8.1 μM | 6.3 μA mM^-1^ cm^-2^ | 3s | NR | Yang et al. (2016b) |
| PMo_12/_rGO@GCE | a | no | acidic | 100 μM−20 mM | 10.2 μM | 95.5 μA mM^-1^ cm^-2^ | 5s | NR | Yang et al. (2016a) |
| PMo_12_/PEI@ITO | a | no | 5 | 3.75x10^-4^-3.13x10^-2^ mg/mL | 0.2 μg mL^-1^ | NR | NR | 100 cycles | Hao et al. (2017) |
| NENU-5-KB^**^@GCE | a | no | 7.4 | 10 μM−20 mM | 1.03 μM | 33.77 μA mM^-1^ | NR | 4h | Wang et al. (2018a) |
| PMo_12_/AuNPs/rGO@GCE | a | no | 6 | < 100 μM | 56 nM | 740.8 μA mM^-1^ cm^-2^ | NR | NR | Berbéc et al. (2018) |
| Ag_4_L^a^_5_SiW_12_@CPE  Ag_3_L^a^_4_PW_12_@CPE  Ag_6_L^b^_6_PMo_12_@CPE  Ag_4_L^a^_2_L^b^_4_GeMo_12_@CPE | a  a  a  a | no  no  no  no | acidic | 3.2x10^-3^-8x10^-3^ M  2x10^-4^-4x10^-3^ M  2x10^-4^-4x10^-3^ M  1x10^-3^-6x10^-3^ M | 5.54x10^-6^ M  1.28x10^-6^ M   4.95x10^-6^ M   5.45x10^-6^ M | 1.47 μA mM^-1^  4.69 μA mM^-1^  12.97 μA mM^-1^  11.25 μA mM^-1^ | 4.2s 4.5s   4.2s   4.5s | NR  NR  NR  NR | Tian et al. (2018a) |
| Cu_2_(H_2_bdpm)_2_P_2_W_18_@CPE  (Cu_3_(pdp)_6_Cl_2_)PCuMo_11_@CPE | b  a | no  no | acidic | 0.3-1.2 mM  0.1-1.6 mM | 1.4x10^-5^ M  1.7x10^-5^ M | 83.21 μA mM^-1^  54.21 μA mM^-1^ | 4.2s  4.5s | NR  NR | Tian et al. (2018b) |
| PEI/rGO/AuNPs/P_8_W_48_@ITO | n | no | 7 | < 22.15 mM | 0.31 μM | 74.56 μA mM^-1^ cm^-2^ | NR | NR | Zhang et al. (2019a) |
| MWCNTs/[C_12_Py][PF_6_]/PMo_12_@GCE | a | no | 1 | 400-3000 μM | 241 μM | 2.4 μA mM^-1^ cm^-2^ | NR | 100 cycles day  / 5 days | Feizy and Haghighi, (2019) |
| {K(H_2_O)}_2_{Cu_2_(bim)_2_}_2_P_2_W_18_@GCE | b | no | acidic | 1.2-11.7 mM | 72.1 mM | 0.094 μA mM^-1^ cm^-2^ | 4s | 1 month | Gao et al. (2020a) |
| [Ag(bpy)][{Ag(Hbpy)}_2_AlW_12_@GCE [H_2_en][{Cu(bpy)}_3_AlW_12_@GCE | a  a | no  no | acidic | 1.2-3.2 mM  19.95 μM−0.90 mM | 0.93 μM  0.86 μM | NR  NR | 4s  2s | 1000 cycles  1000 cycles | Gong et al. (2020) |
| [Mo-oxo]n/N-MPC@GCRDE | a | no | 7 | 50 μM−5 mM | 0.23 μM | 2.2 mA mM^-1^ cm^-2^ | 2s | 2 months | Liu et al. (2020a) |
| Ag-Fe_2_O_3_/PMo_12_/rGO@GCE | a | local river | 6.8 | 0.3-3.3 mM | 0.2 μM | 271 μA mM^-1^ cm^-2^ | < 5s | NR | Ross and Nqakala, (2020) |
| (Ag_7_bpy_7_Cl_2_)AsW_12_@GCE | a | human serum | 7.4 | 1.43 μM−1.89 mM | 0.48 μM | 484.02 μA mM^-1^ cm^-2^ | 2s | 3 days | Cui et al. (2020) |
| {P_5_W_30_}/Mn/H_2_bimb@GCE  {P_5_W_30_}/Co/H_2_bimb@GCE  {P_5_W_30_}/Cu/H_2_bimb@GCE  {P_5_W_30_}/Zn/H_2_bimb@GCE | k  k  k  k | no  no  no  no | 7 | 1-100 mM  0.5-200 mM  1-30 mM  1.120 mM | 0.44 mM  0.13 mM  0.47 mM  0.62 mM | 0.875 μA mM^-1^  4.35 μA mM^-1^  1.66 μA mM^-1^  1.37 μA mM^-1^ | 5s  1s  3s  9s | no  10hours  no  no | Zhu et al. (2021) |
| [Cu(MET)_2_]Mo_8_@CPE  [Cu(bpy)]Mo_2_@CPE | j  - | no  no | acidic | 0.1x10^-3^-92.1x10^-2^ M  0.2x10^-3^-1.9x10^-2^ M | 6.65x10^-5^ M  8.9x10^-4^ M | 1.475 μA mM^-1^  54.632 μA mM^-1^ | 4.46s  3.28s | NR  NR | Zhang et al. (2021a) |
| Cu_3_(OH)_4_(Ptla)_2_TeMo_6_@CPE  Cu_2_(OH)(Ptep)_2_Mo_8_@CPE | c  - | no  no | acidic | 5x10^-6^-1.15x10^-4^ M  8x10^-6^-1.84x10^-4^ M | 9.77x10^-4^ M  4.52x10^-3^ M | 9.325 μA mM^-1^  25.777 μA mM^-1^ | 4.5s  2.5s | NR  NR | Ying et al. (2021) |

Abbreviations as reported by authors. **[BMIM][PF_6_]:** 1-butyl-3-methylimidazolium hexafluorophosphate; **[C_8_Py][PF_6_]:** n-octylpyridinium hexafluorophosphate; **APS:** 3-aminopropyl(triethoxy)silane; **Au:** gold; **AuNPs:** Au nanoparticles; **B:** ethylamine; **bim:** biimidazole; **bimb:** 1,4-bis(1H-imidazol-1-yl)benzene; **bppy:** 4-(5-(4-bromophenyl)pyridin-2-yl-)pyridine); **bpy:** 4,40-bipyridyl; **CPE:** carbon paste electrode; **en:** ethylenediamine; **GCE:** glassy carbon electrode; **GCRDE:** glassy carbon rotating disk electrode; **GE:** graphite electrode; **H_2_bdpm:** 1,1′-bis(3,5-dimethyl-1H-pyrazolate)methane; **ITO:** indium tin oxide electrode; **L^a^:** 2,3-diphenylpyrazine; **L^b^:** 2,3-diphenylquinoxaline; **MET:** 4-(3-imidazol-1-yl-ethyl)-4H-[1,3,4]triazole; **MPS:** 3-mercapto-1-propanesulfonic acid; **MWCNTs:** multi walled carbon nanotubes; **NENU-5-KB:** POM-based metalorganic framework hybridized with Ketjenblack; **N-MPC:** nitrogen-doped mesoporous carbon; **NR:** not reported; **OMC:** ordered mesoporous carbon; **PANI:** polyaniline; **PdNPs:** Pd nanoparticles; **pdp**: 4-propyl-4,5-dihydro-1H-pyrazole; **PEI:** Poly(ethyleneimine); **Pt:** platinum; **Ptep:** 1-[2-(3-pyridin-4-yl-[1,2,4]triazol-4-yl)-ethyl]-piperazin; **Ptla:** 2-(3-pyridin-4-yl-[1,2,4]triazol-4-yl)-ethylamine; **rGO:** reduced graphene oxide; **SWCNTs:** single walled carbon nanotubes**.**

^*^ Na_6_[H_4_Fe_4_(PMo_9_O_34_)_2_(H_2_O)_2_].H_2_O; ^**^[Cu_2_(BTC)_4/3_(H_2_O)_2_]_6_[H_3_PMo_12_O_40_]

**POM archetype** according to the legend of Figure 2: a) Keggin, b) Dawson, c) Anderson, j) γ-octamolybdate, and including n) crown-shape, k) Preyssler and -) unspecified type.

Table S2-POM-based electrochemical sensors for NO_2_^-^ detection.

| **Hybrid material@Electrode** | **POM**  **archetype** | **Matrix** | **pH** | **Linear**  **range** | **Detection**  **limit** | **Sensitivity** | **Response**  **time** | **Stability**  **studies** | **References** |
| --- | --- | --- | --- | --- | --- | --- | --- | --- | --- |
| P_2_Mo_18_/OMC@GCE | b | no | acidic | 5.34-24000 μM | 1.78 μM | NR | < 25s | NR | Zhou et al. (2007) |
| PMo_12_/BC@PE | a | no | acidic | Up to 14.28 mM | 1.0x10^-4^ M | NR | NR | 100 cycles | Liang et al. (2009) |
| RuSiW_10_/PEI@ITO | a | no | acidic | 0.25–1.25 mM | 0.1 mM | 39.7 μA mM^-1^ | < 5s | NR | Ma et al. (2010) |
| P_2_W_18_/PVA@ITO | b | no | acidic | 0.1-1.5 mM | 0.96 μM | 10.95 μA mM^-1^ cm^-2^ | 5s | 100 cycles  / 2 months | Cao et al. (2012) |
| PEI/PSS/PDDA/P_2_W_17_V/CNTs@ITO | b | juices, milk, sausage,  pickled vegetable | 7.0 | 5x10^-8^-2.13x10^-3^ M | 0.0367 μM | 0.35 mA mM^-1^ | 2s | 150 cycles  / 50 days | Zhang et al. (2013) |
| SiMo_12_/rGO@ITO | a | tap water | acidic | 33.3-632.7 μM^-1^ | 7.73 μM | 0.651 μA mM^-1^ | 4s | 100 cycles | Guo et al. (2014) |
| PPD/SiW_11_@BDDE | a | river water | acidic | 40-4x103 M | 20 μM | NR | NR | no | Sahraoui et al. (2015) |
| PMo_11_/ox-SWCNTs@GCE | a | no | 1 | 3.0x10^-5^-1.6x10^-2^ M | 3.0x10^-5^ M | 44.41 mA mM^-1^ | NR | 1 month | Boussema et al. (2016) |
| PEI/PMo_9_V_3_/PEDOT/AuNPs@GCE | a | tap and mineral water,  apple juice, milk, yoghurt | 5.1 | 2.5x10^-9^-1.43x10^-3^ M | 1 nM | 0.67 μA μM^-1^ | 0.6s | 100 cycles  / 20 days | Zuo et al. (2016) |
| Cu_2_(H_2_bdpm)_2_P_2_W_18_@CPE  (Cu_3_(pdp)_6_Cl_2_)PCuMo_11_@CPE | b  a | no | acidic | 0.012-0.068 mM  0.004-0.06 mM | 4.9 x10^-5^ M  8.7 x10^-5^ M | 390.45 μA mM^-1^   575.17 μA mM^-1^ | 3.5s  2.7s | NR  NR | Tian et al. (2018b) |
| Ag_4_L^a^_5_SiW_12_@CPE  Ag_3_L^a^_4_PW_12_@CPE  Ag_6_L^b^_6_PMo_12_@CPE  Ag_4_L^a^_2_L^b^_4_GeMo_12_@CPE ( | a  a  a  a | no | acidic | 2.6x10^-5^-8x10^-3^ M  6x10^-4^-7.2x10^-3^ M  6x10^-4^-7.2x10^-3^ M  3.2x10^-3^-8x10^-3^ M | 9.22x10^-5^ M  3.19x10^-5^ M  7.55x10^-6^ M  8.74x10^-6^ M | 315.18 μA mM^-1^  232.82 μA mM^-1^  87.75 μA mM^-1^  162.46 μA mM^-1^ | 4.2s  4.5s  4.2s  4.5s | NR  NR  NR  NR | Tian et al. (2018a) |
| P_2_W_18_/Zn/dbt@CPE  PW_12_/Cd/dbt@CPE  SiW_12_/Cd/dbt@CPE | b  a  a | no  no  no | acidic | 0.012-0.092 mM  0.016-0.092 mM  0.008-0.092 mM | 2.6x10^-5^ M  3.3x10^-5^ M  2.2x10^-5^ M | 14.19 μA mM^-1^  26.54 μA mM^-1^   1.26 μA mM^-1^ | 3.3s   2.3s   1.6s | NR  NR  NR | Ying et al. (2019) |
| MWCNTs/[C_12_Py][PF_6_]/PMo_12_@GCE | a | no | 1 | 100-23000 μM | 57 μM | 8.2 μA mM^-1^ cm^-2^ | NR | 100 cycles day  / 5 days | Feizy and Haghighi, (2019) |
| SWNTs/ILC_12_/PMo_12_@GCE  SWNTs/ILC_8_/PMo_12_@GCE  SWNTs/ILC_4_/PMo_12_@GCE | a  a  a | no  no  no | acidic | 0.02-4.33 mM  0.02-1.58 mM  0.24-0.99 mM | 1.3 μM  1.3 μM  1.3 μM | 0.019 μA mM^-1^   0.029 μA mM^-1^   0.43 μA mM^-1^ | 5.8s  6.1s  6.5s | 100 cycles  100 cycles  100 cycles | Wang et al. (2019b) |
| PMA/MoS_2/_rGO@GCE | a | lake water | acidic | 0.5-8000 μM | 0.2 μM | 3.79 μA mM^-1^ cm^-2^ | NR | 1 month | Xu, (2019) |
| rGO/PANI/AsM@GCE | - | beverages,  cucumber extract, water | 4 | 25–7500 μM | 10.71 μM | 0.803 μA μM^-1^ cm^-2^ | NR | 2 months | Suma et al. (2019) |
| Zn_2_(bte)_4_SiMo_12_@CPE | a | no | acidic | 8x10^-3^-9.2x10^-2^ M | 6.1x10^-3^ M | 38.33 μA mM^-1^ | 2.3s | NR | Mou et al. (2019) |
| Cu^II^_4_(btmc)(ctcm)_4_Mo_8_@ CPE  Cu^II^_4_(mct)_2_(ctcm)_2_(H_2_O)_6_Mo_8_@CPE  Cu^II^(dm_4_bt)Mo_3_@CPE  Co^II^(dm_4_bt)Mo_2_@CPE Co^II^(H_2_bdpm)Mo_2_@CPE | -  -  -  -  - | no  no  no  no  no | acidic | 0.008-0.08 mM  0.008-0.08 mM  0.004-0.092 mM 0.004-0.088 mM  0.004-0.088 mM | 1.4x10^-7^ M  5.6x10^-7^ M  1.135x10^-7^ M  1.264x10^-6^ M  4.26x10^-8^ M | NR  NR  NR  NR  NR | 2.07s  2.25s  2.7s  3.6s  4.5s | NR  NR  NR  NR  NR | Wang et al. (2020b) |
| Ag(Py_2_Piz)_2_PW_12_@GCE  Ag_4_(AcyPh_)4_SiMo_12_@GCE  Ag_2_(Py_3_Piz)_2_(H_2_O)_2_SiMo_12_@GCE  Ag/Py_2_TTz/PMo_12_@GCE | a  a  a  a | no  no  no  no | acidic | 1.2x10^-2^-9.6 x10^-2^ mM  8x10^-3^-8x10^-2^ mM  4x10^-3^-5.6x10^-2^ mM  2.8x10^-2^-9.6x10^-2^ mM | 2.2x10^-4^ M   2.0x10^-4^ M  2.26x10^-4^ M  1.2x10^-5^ M | 153.049 μA mM^-1^  169.135 μA mM^-1^  10766.4 μA mM^-1^  28.606 μA mM^-1^ | 1.17s  1.35s  1.98s  0.99s | NR  NR  NR  NR | Mou et al. (2020) |
| [Cu(MET)_2_]Mo_8_@CPE  [Cu(bpy)]Mo_2_@CPE | j  - | no  no | acidic | 0.4x10^-6^-9.6x10^-5^ mM  0.4x10^-6^-9.6x10^-5^ mM | 8.45x10^-5^ M  8.75x10^-4^ M | 6.932 μA mM^-1^  71.756 μA mM^-1^ | 4.12s  5.06s | NR  NR | Zhang et al. (2021a) |
| Cu_3_(OH)_4_(Ptla)_2_TeM_o6_@CPE  Cu_2_(OH)(Ptep)_2_Mo_8_@CPE | c  - | no  no | acidic | 1.2x10-5-9.6x10^-5^ M  5x10^-6^-1.15x10^-4^ M | 1.57x10^-4^ M   1.02x10^-2^ M | 15.241 μA mM^-1^   66.905 μA mM^-1^ | 4.5s  2.5s | NR  NR | Ying et al. (2021) |
| {Cu^I^_5_[4-atrz]_6_}^5+^-PMo_12_@GCE  {Cu^I^_5_[4-atrz]_6_}^5+^-PW_12_@GCE  {Cu^I^_5_[4-atrz]_6_}^5+^-SiW_12_@GCE | a  a  a | no  no  no | acidic | 0.044-0.092 mM  0.036-0.088 mM  0.032-0.096 mM | 1.3x10^-5^ M  2.2x10^-5^ M  1.2x10^-5^ M | 145 μA mM^-1^  97 μA mM^-1^  131 μA mM^-1^ | 3.2s  3.7s  2.8s | 1000 cycles  1000 cycles  1000 cycles | Yang et al. (2021) |
| (bdpy)PW_11_Co/MWCNTs-COOH@GCE | a | mineral and industrial water | 1.5 | 10-1600 μM | 0.63 μM | 17.9 μA mM^-1^ | NR | 220 cycles  / 1 month | Karimi-Takallo et al. (2021) |

Abbreviations as reported by the authors. **[C_12_Py][PF_6_]**: n-dodecyl pyridinium hexafluorophosphate; **4-atrz**: 4- amino-triazole; **AuNPs**: Au nanoparticles; **BC**: bacterial cellulose; **BDDE**: boron doped diamond electrode; **bdpy**: 1,10-(1,4-Butanediyl)dipyridinium; **bpy**: 4,40-bipyridyl; **bte**: 1,2-bis(1,2,4-triazol-1-yl)ethane; **btmc**: 1,4-bis(1,2,4-triazol-1-methyl)cyclohexane; **CNTs**: carbon nanotubes; **CPE**: carbon paste electrode; **ctcm**: C-(4-[1,2,4]Triazol-4-ylmethylcyclohexyl)-methylamine; **dbt**: 2,2’-dimethyl-4,4’-bithiazole; **dm_4_bt**: 2,2′-dimethyl-4,4′-bithiazole; **GCE**: glassy carbon electrode; **H_2_bdpm**: 1,1′-bis(3,5-dimethyl-1H-pyrazolate)methane; **ILC_n_**: CH_3_N(CH_2_CH_2_OH)_2_(C_n_H2_n+1_) Br (n = 4, 8, 12); **ITO**: indium tin oxide electrode; **L^a^**: 2,3-diphenylpyrazine; **L^b^**: 2,3-diphenylquinoxaline; **mct**: 4-(4-Methyl-cyclohexylmethyl)-4H-[1,2,4]triazole; **MET**: 4-(3-imidazol-1-yl-ethyl)-4H-[1,3,4]triazole; **MWCNTs**: multi walled carbon nanotubes; **NR**: not reported; **OMC**: ordered mesoporous carbon; **ox-SWCNts**: oxidized single walled carbon nanotubes; **PANI**: polyaniline; **PDDA**: poly diallyl dimethyl ammonium; **pdp**: 4-propyl-4,5-dihydro-1H-pyrazole; **PE**: plastic electrode; **PEDOT**: poly(3,4-ethylenedioxythiophene); **PEI**: Poly(ethyleneimine); **PPD**: p-phenylenediamine; **PSS**: poly(styrenesulfonate); **Ptep**: 1-[2-(3-pyridin-4-yl-[1,2,4]triazol-4-yl)-ethyl]-piperazine; **Ptla**: 2-(3-pyridin-4-yl-[1,2,4]triazol-4-yl)-ethylamine; **PVA**: poly(vinyl alcohol); **Py_2_Piz**: 4,5-bis(2-pyridinyl)imidazole; **Py_2_TTz**: 2,5-bis(4-pyridyl)thiazolo[5,4-*d*]thiazole; **Py_3_Piz**: 2-(4-pyridyl)4,5-di(2-pyridinyl)imidazole; **rGO**: reduced graphene oxide; **SWCNTs**: single walled carbon nanotubes.

**POM archetype** according to the legend of Figure 2: a) Keggin, b) Dawson, c) Anderson, j) γ-octamolybdate, and -) unspecified type.

Table S3 - POM-based electrochemical sensors for other oxidants species.

| **Target** | **Hybrid material@Electrode** | **POM**  **archetype** | **Matrix** | **pH** | **Linear**  **range** | **Limit of  detection** | **Sensitivity** | **Response**  **time** | **Stability**  **studies** | **References** |
| --- | --- | --- | --- | --- | --- | --- | --- | --- | --- | --- |
| **BrO_3_^-^** | MWNTs/PMo_12_@PGE | a | no | acidic | 5 μM-15 mM | 0.5 μM | 760.9 μA mM^-1^ cm^-2^ | < 2s | NR | Li et al. (2006) |
|  | P_2_Mo_18_/OMC@GCE | b | no | acidic | 2.77-4000 μM | 0.922 μM | NR | NR | NR | Zhou et al. (2007) |
|  | SWCNT/SiMo_12_/[Cu(bpy)_2_]^2+^@GCE | a | no | 1 | 10-200 nM | 1.1 nM | 6.7 nA nM^-1^ | < 4s | 30 days | Salimi et al (2009) |
|  | SiNiW_11_/cysteamine@Au | a | no | acidic | 0.014 - 13.58 mM | 14.88μM | NR | 10s | no | Chen et al. (2009) |
|  | Cu_2_(H_2_bdpm)_2_P_2_W_18_@CPE  (Cu_3_(pdp)_6_Cl_2_)PCuMo_11_@CPE | b  a | no | acidic | 0.02-0.0482 mM   0.036-0.076 mM | 1.8x10^-5^ M  2.3x10^-6^ M | 201.53 μA mM^-1^   62.94 μA mM^-1^ | 4.1s  4.4s | NR  NR | Tian et al. (2018b) |
|  | Ag_4_L^a^_5_SiW_12_@CPE  Ag_3_L^a^_4_PW_12_@CPE  Ag_6_L^b^_6_PMo_12_@CPE | a  a  a | no | acidic | 1.2x10^-5^-5.8x10^-3^ M  3.4x10^-4^-8x10^-3^ M  4x10^-4^-8x10^-3^ M | 5.61x10^-6^ M  1.69x10^-5^ M  2.28x10^-6^ M | 12.66 μA mM^-1^  101.67 μA mM^-1^  10.91 μA mM^-1^ | 4.2s  4.5s  4.2s | NR  NR  NR | Tian et al. (2018a) |
|  | MWCNTs/[C_12_Py][PF_6_]/PMo_12_@GCE | a | no | 1 | 100-55000 μM | 21 μM | 24 μA mM^-1^ cm^-2^ | NR | 100 cycles a day  / 5 days | Feizy and Haghighi, (2019) |
|  | SWNTs/ILC_12_/PMo_12_@GCE  SWNTs/ILC_8_/PMo_12_@GCE  SWNTs/ILC_4_/PMo_12_@GCE | a  a  a | no  no  no | acidic | 0.2-3.84 mM  0.2-2.64 mM  0.2-1.71 mM | 1.3 μM  1.3 μM  1.3 μM | 0.023 μA mM^-1^  0.045 μA mM^-1^  0.104 μA mM^-1^ | 3.6s  4.0s  4.2s | 100 cycles  100 cycles  100 cycles | Wang et al. (2019b) |
| **IO_3_^-^** | P_2_Mo_18_/OMC@GCE | b | no | acidic | 1.13-6250 μM | 0.377 μM | NR | NR | NR | Zhou et al. (2007) |
|  | MWCNTs/[C_8_Py][PF_6_]/PMo_12@_GCE | a | no | 2.59 | 0.02-2 mM | 15 μM | 14.81 μA mM^-1^ | < 2s | 20 times a day  /5 days | Haghighi et al. (2010) |
|  | CoSal/SiW_12_@CPE | a | no | 0.5 | 0.05-5 μM | 48 nM | 10.09 μA mM^-1^ cm^-2^ | NR | 50 cycles | Kakhki and Shams, (2013) |
|  | PMoA/PEI@ITO | a | table salt | 5 | 5.00x10^-4^-7.5x10^-2^ mg/mL | 0.1 μg mL^-1^ | NR | NR | 100 cycles | Hao et al. (2017) |
|  | P_2_W_17_V/CNTs/CuONPs | b | table salt | 2.5 | 1.25x10^-7^-1.63x10^-4^ M | 1.5x10^-8^ M | 4.82 μA mM^-1^ | 2s | 100cycles  /60 days | Wang et al. (2018b) |
|  | MWCNTs/[C_12_Py][PF_6_]/PMo_12_@GCE | a | no | 2.50 | 10-1000 μM | 2 μM | 106 μA mM^-1^ cm^-2^ | NR | 100 cycles day  / 5 days | Feizy and Haghighi, (2019) |
|  | SWNTs/ILC_12_/PMo_12_@GCE  SWNTs/ILC_8_/PMo_12_@GCE  SWNTs/ILC_4_/PMo_12_@GCE | a  a  a | no  no  no | acidic | 0.004-0.73 mM  0.008-0.59 mM 0.008-0.18 mM | 0.9 μM  0.9 μM  0.9 μM | 3.76 μA mM^-1^  4.56 μA mM^-1^   5.96 μA mM^-1^ | 2.5s  2.8s  2.9s | 100 cycles  100 cycles  100 cycles | Wang et al. (2019b) |
|  | (bdpy)SiW_11_Ni/P-rGO@GCE | a | mineral and tap water, iodized salt | 1.5 | 10-1600 μM | 0.47 nM | 28.1 μA mM^-1^ | NR | 200cycles/1 month | Sharifi et al. (2021) |
| **IO_4_^-^** | MWCNTs/[C_12_Py][PF_6_]/PMo_12_@GCE | a | no | 2.50 | 10-100 μM | 4 μM | 204μA mM^-1^ cm^-2^ | NR | 100 cycles a day  /5 days | Feizy and Haghighi, (2019) |
| **ClO_3_^-^** | PMo_11_V/PR@ITO | a | no | 2.5 | 0-1000 μM | 220 μM | 0.022 μA μM^-1^ | NR | 8 weeks | Trammell et al (2017) |
|  | MWCNTs/[C_12_Py][PF_6_]/PMo_12_@GCE | a | no | 1 | 1000-6000 μM | 486 μM | 0.42 μA mM^-1^ cm^-2^ | NR | 100 cycles a day  / 5 days | Feizy and Haghighi, (2019) |
| **S_2_O_8_^2-^** | SiMo_12_/rGO@ITO | a | tap water | acidic | 1.33 to 19.95 μM | 0.129 μM | 7.84E^-3^ μA μM^-1^ | 4s | 100 cycles | Guo et al. (2014) |
|  | SiMo_12_/CS/rGO@ITO | a | tap and lake water | acidic | 0.67-30.62 μM | 0.05 μM | 0.0448 μA μM^-1^ | 6s | 10 min | Guo et al. (2015b) |
|  | SiMo_12_/PEDOT/rGO@ITO | a | tap and lake water | acidic | 1.5 - 132 μM | 0.48 μM | 0.22 μA μM^-1^ cm^-2^ | 6s | 15 min | Guo et al. (2020) |
| **PO_4_^3-^** | Mo_8_@PE | - | saline and seawater | acidic | 1 - 75 nM | 6.1 nM | NR | < 5min | NR | Figueiredo et al. (2021) |

Abbreviations as reported by the authors. **[C_12_Py][PF_6_]**: n-dodecyl pyridinium hexafluorophosphate; **[C_8_Py][PF_6_]:** n-Octylpyridinium hexafluorophosphate; **Au**: gold electrode; **bdpy**: 1,10-(1,4-butanediyl)dipyridinium; **bpy**: 4,40-bipyridyl; **CNTs**: carbon nanotubes; **CoSal**: N,N'-bis(salicylidene)-1,2-phenylenediaminocobalt (III); **CS**: Chitosan; **CuONPs**: CuO nanoparticles; **GCE**: glassy carbon electrode; **H_2_bdpm**: 1,1′-bis(3,5-dimethyl-1H-pyrazolate)methane; **ILCn**: CH_3_N(CH_2_CH_2_OH)_2_(C_n_H_2n+1_) Br (n = 4, 8, 12); **ITO**: indium tin oxide electrode; **L^a^**: 2,3-diphenylpyrazine; **L^b^**: 2,3-diphenylquinoxaline; **NR**: not reported; **OMC**: ordered mesoporous carbon; **pdp**: 4-propyl-4,5-dihydro-1H-pyrazole; **PE**: plastic electrode; **PEDOT**: poly(3,4-ethylenedioxythiophene); **PEI**: Poly(ethyleneimine); **P-rGO**: phosphorus-doped electrochemically reduced graphene oxide; **PGE**: pencil graphite electrode; **PR**: para-rosaniline acetate dye; **SWCNTs**: single walled carbon nanotubes.

**POM archetype** structure according to the legend of Figure 2: a) Keggin, b) Dawson and -) unspecified type.

Table S4 - POM-based electrochemical sensors for biomolecules and bio-related species.

| **Target** | **Hybrid material@Electrode** | **POM archetype** | **Matrix** | **pH** | **Linear**  **range** | **Limit of  detection** | **Sensitivity** | **Response time** | **Stability**  **studies** | **References** |
| --- | --- | --- | --- | --- | --- | --- | --- | --- | --- | --- |
| **Dopamine** | P_2_W_16_V_2_/Au-PdNPs@ITO | b | serum | 7 | 2.10x10^-6^-2.06x10^-3^ M | 0.83 μM | NR | NR | 300 cycles | Zhou et al. (2013) |
|  | PMo_9_V_3_/PtNPs@ITO | a | dopamine hydrochloride injection | 6.5 | 4.2x10^-7^-1.3x10^-3^ M | 1.3x10^-7^ M | 1.2 μA μM^-1^ | 0.8s | 100 cycles | Li et al. (2013) |
|  | PMo_11_V/PEI/CoTsPc-@ITO | a | blood serum | 6.5 | 8.3x10^-8^-5.0x10^-4^ M | 1.3x10^-8^ M | 0.82μA μM^-1^ | < 1.5s | 500 cycles | Zhu et al. (2013) |
|  | PMo_12_/PEI@ITO | a | serum | 5 | 5.70x10^-4^-3.80x10^-2^ mg/mL | 0.2 μg mL^-1^ | NR | NR | 100 cycles | Hao et al. (2017) |
|  | Cu_3_Mo_5_P_2_/rGO@GCE | - | artificial cerebrospinal fluid, human blood serum | 7 | 1x10^-6^-2x10^-4^ M | 80.4x10^-9^ M | 0.373x10^-3^mA M^-1^ cm^-2^ | NR | 1 week | Zhang et al. (2017b) |
|  | PMo_9_V_3_/Pd-PtNPs/MWCNTs@ITO | a | human serum and dopamine hydrochloride injections | 7.3 | 2.50x10^-8^-1.78x10^-4^ M | 1.25x10^-8^ M | 0.690 μA μM^-1^ | <1.0s | 100 cycles | Jiao et al (2018) |
|  | PVIM-Co_5_POM^*^/N-CNTs@GE | a | dopamine hydrochloride injections | 7.4 | 0.0005-600 μM | 500 pM | NR | NR | 100 cycles | Thakur et al. (2018) |
|  | GeW_12_/CFMWCNTs/Nafion@GCE | a | no | 3.6 | 10-1000 μM | 1.23 μM | NR | NR | 180 cycles | Shi et al. (2019) |
|  | PtNPs/IMo_6_/GO@GCE | c | human serum | 1.3 | 4-750 μM | 0.22 μM | NR | 1.4s | 100 cycles/  20 days | Zhang et al. (2019c) |
|  | P_2_W_17_V/CS@ITO | b | human serum | 7.0 | 1.25x10^-8^-3.04x10^-4^ M | 0.18 μM | 0.23 μA μM^-1^ | 2.2s | 100 cycles | Wang et al. (2019c) |
|  | Ce-POM^**^/CFMWCNTs@GCE | - | no | 7.0 | 10-100 μM | 1.61 μM | NR | NR | 180 cycles | Liu et al. (2020b) |
|  | V_10_O_28_/NU-902@FTO | e | no | 4.5 | 25-400 μM | 2.1 μM | 85 μA mM^-1^ cm^-2^ | NR | 20 cycles | Ho et al. (2020) |
|  | Ce-POM^***^/CFMWCNTs@GCE | - | no | 3.0 | 4−100 μM | 0.053 μM | NR | NR | 100 cycles/  7 days | Jiang et al. (2020) |
|  | [Ag_5_(trz)_4_]_2_·PMo_12_/SWCNTs-COOH@GCE | a | human serum | 7.0 | 0.05 to 100 μM | 8.6 nM | NR | NR | 100 cycles/  1 month | Zhou et al. (2021) |
|  | PMo_12_[6]catenane/rGO@GCE | a | human serum | 2.0 | 1 to 44 μM | 0.065 μM | NR | < 2s | 50 cycles/  1 week | Han et al. (2021) |
| **Ascorbic acid** | PEI/RuSiW_10_@ITO | a | no |  | 0.25–2.25 mM | 0.08 mM | 18.9 μA mM^-1^ | < 5s | NR | Ma et al. (2010) |
|  | [BMIM]_6_-P_2_Mo_18_@GCE | b | no | 0-7 | 0.1μM-22 mM | <0.1 μM | 63 nA μM^-1^ | <9s | 2 weeks | Ammam and Easton, (2011b) |
|  | SiNiW_11_/cysteamine@Au | a | no |  | 0.014 - 18.58 mM | 14.60μM | NR | 10s | no | Chen et al. (2009) |
|  | P_2_W_16_V_2/_Au-PdNPs@ITO | b | fruit juice | 7 | 1.20x10^-6^-1.61x10^-3^ M | 0.43 μM | NR | NR | 300 cycles | Zhou et al. (2013) |
|  | PMo_12_/GS@GCE | a | vitamin C tablets | 7.2 | 1x10^-6^-8x10^-3^ M | 0.5x10^-6^ M | NR | 5-8s | 1 month | Zhang et al. (2014) |
|  | PW_12_/PEI@ITO | a | soft fruit drinks | 5 | 1.0x10^-3^-1.2x10^-2^ mg/mL | 6.4x10^-4^ mg/mL | NR | NR | NR | Xu et al. (2015) |
|  | PMo_12_/PEI@ITO | a | fruit juice | 5 | 8.8x10^-4^-4.3x10^-2^ mg/mL | 0.43 μg mL^-1^ | NR | NR | 100 cycles | Hao et al (2017) |
|  | PtNPs/IMo_6_/GO@GCE | c | human serum | 1.3 | 50-4000 μM | 6.42 μM | NR | 1.2s | 100 cycles/  20 days | Zhang et al. (2019b) |
|  | P_2_Mo_17_V/Ru(bpy)_3_/CS-PdNPs@ITO | b | juice | 7 | 0.1254-118 μM | 0.1 μM | 153.1 μA mM^-1^ cm^-2^ | < 2s | 30 days | Zhang et al. (2021b) |
| **Creatinine** | MIP/AgNPs/PW_12_/rGO@GCE | a | saliva and serum | 6 | 0.05-1.5 nM | 1.51x10^-11^ M | NR | NR | 10 days | Zhang et al. (2018) |
| **Cholesterol** | PVIM-Co_5_POM^*^/N-MPC@GE | a | human blood serum | 7.4 | 1 fM-200 nM | 1 fM | 210 μA μM^-1^ cm^-2^ | 5s | 100 cycles | Thakur et al. (2019) |
| **Bilirubin** | MIP/PW_12_/C_3_N_4_NTs@GCE | a | human plasma | 4.0 | 1.0 pM- 0.1 nM | 0.1 pM | NR | NR | 60 days | Yola et al. (2017) |
| **Xanthine** | Fc/PMo_6_W_6/_rGO@GCE | a | human urine | 6.0 | 50 nM- 39.8 μM | 10.1 nM | 0.147 μA μM^-1^ | NR | 100 cycles/  2 weeks | Zhu et al. (2019) |
| **Glucose** | Fe_4_POM^****^/poly(1,8 DAN)/GOx@GE | a | no | 2.5 | 2.5-20 mM | 1.2mM | 19.8 μA M^-1^ cm^-2^ | < 40s | no | Turdean et al. (2002) |
|  | MPS/B/PFe_3_Mo_9_/B/GOx@Au | a | no | 6.2 | Up to 50 mM | NR | 13.3 μA M^-1^ | < 20s | NR | Turdean and Popescu, (2012) |
|  | PMo_12_/rGO/GOx@GCE | a | no |  | 2-20mM | 67.9 μM | 14.3 μA mM^-1^ cm^-2^ | 5s | NR | Yang et al. (2016a) |
|  | P_2_Mo_18_/PMA/MWCNTs@GCE | b | no | 7.0 | 1-20 mM | NR | 0.198 mA M^-1^ cm^-2^ | NR | 15days | Boussema et al. (2018) |
|  | PW_9_/PAAC/GOx@GE | a | Fizzy drink, Cherry juice | 6.0 | 0.1-10 mM | 0.099 mM | 66.66 μA mM^-1^ cm^-2^ | NR | 4 weeks | Ayranci et al. (2018) |
|  | Co_2_W_11_O_39_/MWCNTs@GE | a | coke, juice |  | 0.1-10 mM | 1.21 μM | 256.4 μA mM^-1^ cm^-2^ | 6s | 5 weeks | Ayranci et al. (2019) |
| **Uric acid** | Ce-POM^**^/CFMWCNTS@GCE | a | no | 7.0 | 10−100 μM | 5.41 μM | NR | NR | 180 cycles | Liu et al (2020b) |
|  | PtNPs/IMo_6_/GO@GCE | c | human serum | 1.3 | 75-300 μM | 0.72 μM | NR | 1.6s | 100 cycles/20 days | Zhang et al. (2019b) |
|  | Cubix/P_2_W_18_@GCE | b | no | 6.0 | 2.5x10^-7^-6.79x10^-4^ M | 4.97x10^-7^ M | 0.017 μA μM^-1^ | NR | 50 cycles/  30 days | Xu et al. (2021) |
|  | rGO/AuNPs/P_2_W_18_@ITO | b | human serum | 7.0 | 0.5−450 μM | 0.15 μM | 0.487 μA μM^-1^ | NR | 50 cycles/  30 days | Bao et al. (2020b) |
|  | bix/P_2_W_18_@GCE | b | human urine | 3.0 | 2.5x10^-7^-6.99x10^-4^ M | 5.85x10^-7^ M | 0.012 μA μM^-1^ | < 2s | 5 cycles/  4 weeks | Liu et al. (2020c) |
|  | AM-LnSTsPOM/CFMWCNTs@GCE | a | no | 7.0 | 1-100 μM | 1.69 μM | NR | NR | 160 cycles | Cui et al. (2021) |
| **NADH** | AuNPs/PW_12_/OMC@GCE | a | no | 7 | 1-110 μM | 0.41 μM | 10.54 μA mM^-1^ | 4s | two weeks | Zhang et al. (2015) |
|  | Ru(bpy)_3_^2+^/ PMo_12_@ITO | a | no | 7.0 | 2.5x10^-7^-5.0x10^-3^ M | 1.67x10^-8^ M | NR | NR | 21 cycles/  2 weeks | Li et al. (2012) |
|  | Ru(bpy)_3_^2+^/ PMo_12_/mrGO@mGCE | a | yes | 7.4 | 2.0x10^-9^-2.0x10^-3^ M | 0.1 nM | NR | NR | 28 cycles/  1 month | Qian et al. (2014) |
| ***Yersinia***  ***pestis*** | SiW_11_Sn-dATPs@Au  SiW_11_Sn-dGTP@Au SiW_11_Sn-dATP/dGTP@Au P_2_W_17_Sn-dATP@Au  P_2_W_17_Sn-dGTP@Au  P_2_W_17_Sn-dATP/dGTP@Au | a  a  a  b  b  b | no  no  no  no  no  no |  |  | 0.6 nM  0.3 nM  0.7 nM  1.12 nM 1.70 nM  1.50 nM | 21.8 nA nM^-1^  15.6 nA nM^-1^  25.3 nA nM^-1^  3.23 nA nM^-1^  2.43 nA nM^-1^  5.20 nA nM^-1^ | NR  NR  NR  NR  NR  NR | NR  NR  NR  NR  NR  NR | Ortiz et al. (2017) |
| **miRNA21** | PMo_12_-MoS_2/_β-FeOOH@Au | a | human serum | 7.4 | 1 fM-5 nM | 0.11 fM | NR | NR | 10 cycles/  15 days | Jia et al. (2020) |
| **Guanine and Adenine** | PNiW_11_/PDDA/MWCNTs@GCE | a | salmon sperm | 2 | 0.4-135.0 μM  0.4-76.0 μM | 0.24 μM  0.1 μM | NR  NR | NR  NR | NR  NR | Ensafi et al. (2017) |
| **Osteopotin** | PPy/Ti_3_C_2_Tx/PMo_12_@GCE | a | human serum | 7.4 | 0.05- 10000 pg mL^-1^ | 0.98 fg mL^-1^ | NR | NR | 10 cycles/  15 days | Zhou et al. (2019) |
| **L-cysteine** | VMo_12_/[BMIM][PF_6_]@CPE | a | food supplement |  | Up to 1000 μM | 0.085 mM | 20.42 μA mM^-1^ | NR | NR | Ji et al. (2009) |
|  | CoSal/SiW_12_@CPE | a | human serum, urine and  N-acetylcysteine tablets | 5.0 | 0.01–1.0 μM | 4.9 nM | 1.57 μA μM^-1^ | NR | 2 months | Kakhki et al. (2013) |
|  | CoSal/SiW_12_@CPE | a | no | 5 | 0.01-1 μM | 967 nM | 0.16 μA mM^-1^ cm^-2^ | NR | 50 cycles | Kakhki and Shams, (2013) |
| **L-tyrosine and  L- tryptophan** | PW_12_/rGO@GCE | a | human serum | 6 | 1.0 x10^-11^-1.0x10^-9^ M | 2x10^-12^ M | NR | NR | 45 days | Yokus et al. (2016) |
| **Folic acid** | PPy/PMo_2_W_9_/AuNPs@Au | a | human serum,  vitamin supplements | 6.0 | 1.0-44.0 nM | 0.12 nM | NR | NR | NR | Babakhanian et al. (2014) |
|  | PEI/P_2_Mo_16_V_2_/rGO@GCE | b | human serum | 7.4 | 1.0x10^-8^-8.0x10^-4^ M | 2.84x10^-10^ M | 226.47 μA μM^-1^ cm^-2^ | NR | 60 days | Xu et al. (2017) |
| **Cardiac troponin I** | {Mo_368_}/FeOOH/Bi_2_S_3_/AuNPs@ITO | q | human serum |  | 1.00 pg mL^-1^-100 ng mL^-1^ | 0.76 pg mL^-1^ | NR | NR | NR | Bao et al. (2020a) |

Abbreviations as reported by the authors.**[BMIM][PF_6_]**: 1-butyl-3-methylimidazolium hexafluorophosphate; **AgNPs**: silver nanoparticles; **AM-LnSTsPOM**: alkali-metal–lanthanide embedded selenotungstates; **AuNPs**: gold nanoparticles; **Au-PdNPs**: gold and palladium nanoparticles; **B**: ethylamine; **bix**: 1,4-bis(imidazol-1-ylmethyl) benzene; **CFMWCNTs:** carboxyl functionalized multi-walled carbon nanotubes; **CoSal**: N,N'-bis(salicylidene)-1,2-phenylenediaminocobalt (III); **CoTsPc**: cobalt(II) tetrasulfonate phthalocyanine; **CS**: Chitosan; **CS-PdNPs**: Chitosan and palladium nanoparticles; **Fc**: ferrocene; **FTO**: fluorine doped tin oxide; **GCE**: glassy carbon electrode; **GO**: graphene oxide; **GOx**: glucose oxidase; **GS**: graphene sheets; **ITO**: indium tin oxide electrode; **mGCE**: magnetic glassy carbon electrode; **MIP**: molecularly imprinted polymer; **MPS**: 3-mercapto-1-propanesulfonic acid; **mrGO**: magnetic reduced graphene oxide; **MWCNTs**: multi walled carbon nanotubes; **N-CNTs**: nitrogen-doped carbon nanotubes; **N-HCSs**: nitrogen-doping hollow carbon spheres; **N-MPC**: nitrogen-doped mesoporous carbon; **NR**: not reported; **OMC**: ordered mesoporous carbon; **PAAC**: 3-Amino-9-ethylcarbazole polymer film; **PDDA**: poly diallyl dimethyl ammonium; **PdNPs**: palladium nanoparticles; **PEI**: Poly(ethyleneimine); **PMA**: 1-pyrenemethylamine; **PPy**: polypyrrole; **PtNPs**: platinum nanoparticles; **Pt-PdNPs**; platinum and palladium nanoparticles; **PVIM^+^**: poly(vinylimidazolium) cation; **rGO**: reduced graphene oxide; **SWCNTs-COOH:** carboxyl functionalized single walled carbon nanotubes; **trz**: 3-mercapto-1,2,4-triazole.

^*^Na_12_[WCo_3_(H_2_O)_2_(CoW_9_O_34_)_2_]; ^**^[H_2_N(CH_3_)_2_]_8_Na[CeNa(H_2_O)_4_(OH)WO(H_2_O)(B-α-SeW_9_O_33_)2]⋅18H_2_O; ^***^Na_16_H_6_{[Ce_3_W_4_O_10_(H_2_O)_9_-(CH_3_COO)_3_]_2_(Se_2_W_7_O_30_) (B-α-SeW_9_O_33_)_4_}·(C_5_H_8_NBO_3_)·119H_2_O; ^****^Na_6_[H_4_Fe_4_(PMo_9_O_34_)_2_(H_2_O)_2_].H_2_O

**POM archetype** structure according to the legend of Figure 2: a) Keggin, b) Dawson, c) Anderson, d) Lindqvist, e) decavanadate, and including q) hedgehog-shape, and -) unspecified type.

Table S5 - POM-based electrochemical sensors for medicines, pesticides, and toxic contaminants.

| **Target** | **Hybrid material@Electrode** | **POM**  **Archetype** | **Matrix** | **pH** | **Linear**  **range** | **Detection**  **limit** | **Sensitivity** | **Response time** | **Stability**  **studies** | **References** |
| --- | --- | --- | --- | --- | --- | --- | --- | --- | --- | --- |
| **Clenbuterol and Ractopamine** | PV_8_Mo_4_/ZrO_2_@GCE | a | pork | 1.0 | 0.1-1000 μM and 3.0-50 mM | 5.03x10^-9^ M and 9.3x10^-7^ M | NR  NR | NR  NR | two weeks | Zhang et al. (2019c) |
| **Acetaminophen** | AuNPs/PW_12_/OMC@GCE | a | paracetamol tablets | 7 | 1-57 μM | 0.29 μM | 29.75 μA μM^-1^ | < 2s | NR | Zhang et al. (2015) |
|  | PMo_11_V/N-CNTs@GCE | a | no | 2.5 | 1.5x10^-6^-3.9x10^-4^ M | 1.0x10^-6^ M | NR | NR | NR | Fernandes et al. (2017) |
|  | PdNPs/PW_12_/N-HCSs@GCE | a | paracetamol tablets | 7.4 | 0.02 μM- 0.63 μM | 3 nM | 508.46 μA mM^-1^ | NR | 1hour  /2 weeks | Wang et al. (2019d) |
|  | La-GeW_12_/CFMWCNT/Nafion@GCE  Tb-GeW_12_/CFMWCNT/Nafion@GCE | a  a | no  no | 8.0 | 10–1000 μM  10–1000 μM | 1.07 μM  1.08 μM | NR  NR | NR  NR | 180 cycles  180 cycles | Li et al. (2019) |
|  | AuNPs/SiW_11_Cu/MWCNTs@GCE | a | paracetamol tablets, mineral and river water | 7 | 1-35 μM | 0.42 μM | NR | NR | 12 days | Dong et al. (2019) |
|  | Ce-POM^*^/CFMWCNTs@GCE | - | no | 3.0 | 8−600 μM | 2.03 μM | NR | NR | 100 cycles  /7 days | Jiang et al. (2020) |
| **Triclosan** | AuNPs/PW_12_/rGO@GCE | a | wastewater, lake water | 7.0 | 0.5–50.0 nM | 0.15 nM | NR | NR | 30 days | Yola et al. (2015) |
| **Ceftizoxime** | NaP_5_W_30_/rGO@PGE | k | ampoules, blood serum | 3.0 | 1.0x10^-11^-3.0x10^-8^ M | 1.8 pM | NR | NR | 1 month | Rouhani and Soleymanpour, (2021) |
| **Methyldopa** | PMo_12_/rGO@PGE | a | human blood serum, urine, and milk | 2.8 | 4.9x10^-10^-1.0x10^-7^ M | 1.2x10^-10^ M | 7.711 μA μM^-1^ | NR | 2 weeks | Dehnavi and Soleymanpour (2020) |
| **Paroxetine** | PW_12_/rGO@PGE | a | paroxetine tablets, human serum, urine | 7.0 | 8.0x10^-9-^1.0x10^-6^ M | 9.0x10^-10^ M | 6.44 μA μM^-1^ | NR | 15 days | Oghli and Soleymanpour (2020) |
| **Sildenafil** | MIP/AuNPs/ NaP_5_W_30_/MWCNTs@PGE | k | human plasma, milk | 7.0 | 0.1-2.0 nM | 0.033 nM | NR | NR | 10x, 1 month | Rouhani and Soleymanpour, (2020) |
| **Simazine** | MIP/PtNPs/PW_12_/MWCNTs@GCE | a | industrial wastewater | 4.0 | 1.0x10^-10^-5.0x10^-9^ M | 2.0x10^-11^ M | NR | NR | NR | Ertan et al. (2016) |
| **Hydrazine** | P_2_W_17_Fe/PdNPs@ITO | b | no | 2 | 2 μM-3.4 mM | 1.5 μM | 0.24 μA mM^-1^ | < 3.5s | 1 month | Ma et al. (2021) |
| **Hydrazine sulfate and Nitrobenzene** | PtNPs/PMo_12_/OMC@GCE | a | no | 7 | 10-840 μM  3.98-672.55 μM | 3.41 μM  3.82 μM | 2.92 μA mM^-1^  102.62 μA mM^-1^ | NR  NR | 2 weeks | Li et al. (2016) |
| **Hydroquinone, Catechol and  Resorcinol** | rGO/SiW_12_@GCE | a | diphenolic compounds, underground and lake water | 4.5 | 0.2-25 μM  0.2-20 mM  0.4-30 mM | 50 nM  40 nM  90 nM | NR  NR  NR | NR  NR  NR | 6 weeks | Cao et al. (2011) |
| **N-hydroxysuccinimide** | PtNPs/PW_12_/2D-hBN@CPE | a | drinking, lake, and river water | 8.0 | 0.1-300 μM | 60 nM | NR | NR | 45 days | Karimi-Maleh et al. (2020) |
| **Chlorogenic acid** | AuNPs/PW_12_/MacroPC@GCE | a | pharmaceutical | 7.0 | 2.28 nM- 3.24 μM | 2.15 nM | 30554.71 μA mM^-1^ | NR | 2 weeks | Zhang et al. (2017c) |
| **Mycertin** | P_2_W_18/_SnO_2/_AuNPs@ITO | b | juice | 3 | 1-110 μM | 67 nM | NR | 4.0s | 20 cycles  /1 week | Xing et al. (2019) |
| **Ochratoxin A** | MIP/AgNPs/PW_12_/rGO@GCE | a | grape juice and wine | 6.0 | 5.0x10^-11^-1.5x10^-9^ M | 1.6x10^-11^ M | NR | NR | 30 days | Yola et al. (2016) |
| **Citrinin** | MIP/PtNPs/PW_12_/rGO@GCE | a | rye samples | 6.0 | 1.0x10^-12^-1.0x10^-10^ M | 2.0x10^-13^ M | NR | NR | 45 days | Atar et al. (2016) |
| **Propylparaben** | PPy/β-CD/PMo_12_@PGE | a | cleansing micellar solution | 6.0 | 0.2-10 μM | 0.04 μM | NR | NR | 5 cycles | Hatami et al. (2021) |
| **Diphenylamine** | PMo_12_/GO@GCE | a | apple juice | 7.0 | 0.05-400 μM | 6.0 nM | 14.52 μA μM^-1^ cm^-2^ | NR | 2 weeks | Gao et al. (2020b) |
| **Diazinon** | MIP/AuNPs/PW_12_/2D-hBN@GCE | a | fruit juice | 6.0 | 1.00x10^-11^-1.00x10^-8^ M | 3.00x10^-12^ M | NR | NR | 45 days | Medetalibeyoğlu et al. (2020) |
| **Bisphenol A** | AgPMo_12_@Au | a | river water, milk, human serum | 7.4 | 0.001-1 pg mL^-1^ | 0.2 fg mL^-1^ | NR | NR | 7 cycles  /15 days | Song et al. (2020) |
|  | AuNPs/SiW_11_Cu/MWCNTs@GCE | a | Mineral and local river water | 7 | 1-30 μM | 0.89 μM | NR | NR | 12 days | Dong et al. (2019) |
| **γ-Lindane** | MIP/PW_12_/C_3_N_4_NTs@GCE | a | orange juice | 7.0 | 1.0x10^-10^-1.0x10^-8^ M | 2.0x10^-11^ M | NR | 12s | 60 cycles  /60 days | Pelin Böke et al. (2020) |

Abbreviations as reported by the authors. **2D-hBN**: two dimensional hexagonal boron nitride nanosheets; **β-CD**: β-cyclodextrin; **AgNPs**: silver nanoparticles; **Au**: gold electrode; **AuNPs**: gold nanoparticles; **C_3_N_4_NTs**: carbon nitride nanotubes; **GCE**: glassy carbon electrode; **GO**: graphene oxide; **ITO**: indium tin oxide electrode; **MacroPC**: macroporous carbon; **MIP**: molecularly imprinted polymer; **MWCNTs**: multi-walled carbon nanotubes; **N-CNTs**: nitrogen-doped carbon nanotubes; **NR**: not reported; **OMC**: ordered mesoporous carbon; **PdNPs**: palladium nanoparticles; **PGE**: pencil graphite electrode; **PPy**: polypyrrole; **PtNPs**: platinum nanoparticles; **rGO**: reduced graphene oxide.

^*^Na_16_H_6_{[Ce_3_W_4_O_10_(H_2_O)_9_-(CH_3_COO)_3_]_2_(Se_2_W_7_O_30_)(B-α-SeW_9_O_33_)_4_}·(C_5_H_8_NBO_3_)·119H_2_O

**POM archetype** structure according to the legend of Figure 2: a) Keggin, b) Dawson, and including k) Preyssler, and -) unspecified type..

Table S6 - POM-based electrochemical sensors for metal ions.

| **Target** | **Hybrid material@Electrode** | **POM archetype** | **Matrix** | **pH** | **Linear**  **range** | **Limit of  detection** | **Sensitivity** | **Time** | **Stability**  **studies** | **References** |
| --- | --- | --- | --- | --- | --- | --- | --- | --- | --- | --- |
| **Cr^6+^** | Co/{P_4_Mo_6_}_2_@GCE  Ni/{P_4_Mo_6_}_2_@GCE  Cd/{P_4_Mo_6_}_2_@GCE | r  r  r | lake water  no  no | acidic | 0.5-378 μM  0.5-378 μM  0.5-504 μM | 0.026 μM  0.321 μM  0.082 μM | 244 μA mM^-1^  107 μA mM^-1^  208 μA mM^-1^ | 1s  3s  2s | 5.5h  NR NR | Wang et al. (2020c) |
|  | Cu^II^_4_(btmc)(ctcm)_4_Mo_8_@ CPE  Cu^II^_4_(mct)_2_(ctcm)_2_(H_2_O)_6_Mo_8_@CPE  Cu^II^(dm_4_bt)Mo_3_@CPE  Co^II^(dm_4_bt)Mo_2_@CPE  Co^II^(H_2_bdpm)Mo_2_@CPE | -  -  -  -  -  - | no  no  no  no  no | acidic | 0.004-0.032 mM  0.008-0.088 mM  0.004-0.092 mM  0.004-0.032 mM  0.004-0.044 mM | 7.4x10^-8^ M  2.5x10^-7^ M 6.5x10^-7^ M 7.35x10^-6^ M  1.03x10^-6^ M | 15.540 μA mM^-1^  27.947 μA mM^-1^  46.993 μA mM^-1^  82.244 μA mM^-1^  91.101 μA mM^-1^ | 2.03s  1.52s   1.9s  1.6s  1.44s | NR  NR  NR  NR  NR | Wang et al. (2020b) |
|  | (H_2_bpp)_2_[Na_4_Fe(H_2_O)_7_]FeP_4_Mo_6_@GCE  (H_2_bpp)_6_(bpp)_2_]FeP_4_Mo_6_@GCE | r  r | lake water  no | acidic | 2-2610 μM  2-2610 μM | 0.174 μM  0.33 μM | 0.117μA mM^-1^  0.074 μA mM^-1^ | 1  NR | NR  NR | Xin et al. (2020) |
|  | H_3_[Cu_2_(4-dpye)_2_PMo_12_@CPE  H[Cu_2_(4-Hdpye)_2_PMo_12_@CPE | a  a | no  no | acidic | 0.5-6000 μM  0.5-6000 μM | 1.27x10^-7^ M  1.71x10^-7^ M | 0.312 μA mM^-1^  0.278 μA mM^-1^ | NR  NR | NR  NR | Liu et al. (2021) |
|  | {P_4_Mo_6_}/Cu/Mn/BBTZ@GCE  {P_4_Mo_6_}/Cu/Mn/BBTZ@GCE  {P4Mo_6_}/Na/Mn/BBTZ@GCE {P_4_Mo_6_}/Na/Mn/BBTZ@GCE | r  r  r  r | lake water  lake water | 0  1-5 0  1-5 | up to 1 μM  up to 1 μM | 1.59 nM  <15 nM   2.91 nM  < 24 nM | 11.08 μA mM^-1^  < 132 μA mM^-1^  119.87 μA mM^-1^  < 111 μA mM^-1^ | NR  NR  NR  NR | 10h  10h | Niu et al. (2021) |
|  | Cu_2_(OH)(Ptep)_2_Mo_8_@CPE | - | no | acidic |  | 1.34x10^-4^ M | -5.508 μA mM^-1^ | 1.8s | NR | Ying et al. (2021) |
|  | {Cu^I^_5_[4-atrz]_6_}^5+^-PMo_12_@GCE {Cu^I^_5_[4-atrz]_6_}^5+^-PW_12_@GCE  {Cu^I^_5_[4-atrz]_6_}^5+^-SiW_12_@GCE | a  a  a | no  no  no | acidic | 0.008-0.1 mM  0.012-0.036 mM 0.006-0.1 mM | 5.4x10^-6^ M 5.4x10^-6^ M  4.2x10^-6^ M | 145 μA mM^-1^  148 μA mM^-1^   242 μA mM^-1^ | 2.1s  3.2s  1.4s | 1000 cycles  1000 cycles  1000 cycles | Yang et al. (2021) |
| **Cd^2+^ and**  **Pb^2+^** | PW_12_/Cys@Au | a | industrial wastewater | acidic | 0.01 - 0.2 μM and  0.01 - 0.2 μM | 9.0 nM and 4.0 nM | 38.3 μA mM^-1^ and 234 mA mM^-1^ | NR | 1 month | Dianat et al (2019) |

Abbreviations as reported by the authors. **4-atrz**: 4- amino-triazole; **Au**: gold, **BBTZ**: 1,4-bis(1,2,4-triazol-1-ylmethyl) benzene; **bpp**: 1,3-bi(4-pyridyl)propane; **btmc**: 1,4-bis(1,2,4-triazol-1-methyl)cyclohexane; **CPE**: carbon paste electrode; **ctcm**: C-(4-[1,2,4]Triazol-4-ylmethylcyclohexyl)-methylamine; **cys**: cysteine; **dm_4_bt**: 2,2′-dimethyl-4,4′-bithiazole; **dpye**: N,N’-bis (4-pyrimidinecarboxamido)-1,2-ethane; **GCE**: glassy carbon electrode; **mct**: 4-(4-Methyl-cyclohexylmethyl)-4H-[1,2,4]triazole; **NR**: not reported; **Ptep**: 1-[2-(3-pyridin-4-yl-[1,2,4]triazol-4-yl)-ethyl]-piperazine.

**POM archetype** structure according to the legend of Figure 2: a) Keggin and including r) hourglass type and -) unspecified type.

Table S7 - POM-based absorption optical sensors.

| **Targets** | **POM or POM**  **hybrid material** | **POM archetype** | **Matrix** | **Chromogenic substrates/ reagents** | **λ (nm)** | **pH** | **Linear**  **range** | **Detection**  **limit** | **Reaction time** | **Stability**  **studies** | **References** |
| --- | --- | --- | --- | --- | --- | --- | --- | --- | --- | --- | --- |
| **H_2_O_2_ and Glucose** | PW_12_ | a | no | TMB  GOx | 652 | 3.0  7.0 and 3.0 | 1.34x10^-7^-6.7x10^-5^ M  1x10^-7^-1x10^-4^ M | NR  NR | 10 min  30 +15 min | NR  NR | Wang et al. (2012) |
|  | SiW_12_ | a | human blood | TMB  GOx | 651 | 4.0  7.0 and 4.0 | 1-20 μM  1-10 μM | 0.4 μM  0.5 μM | 5 min | NR  NR | Liu et al. (2012) |
| **H_2_O_2_ and Citric acid** | Ni_4_(Trz)_6_/SiW_12_/PDDA-rGO | a | orange juice | TMB  H_2_O_2_, TMB | 668  668 | 2.5  2.5 | 1-100 μM  1-60 μM | 0.49 μM  2.07 μM | 10 min  10 min | NR  5 runs | Tong et al. (2020) |
| **H_2_O_2_ and Sarcosine** | PMo_4_V_8_/FA | a | urine | TMB | 652  652 | 4.0  7.3 and 4.0 | 0.007-4 μM 0.2-500 μM | 0.012 μM  0.311 μM | 2 min  30 + 4 min | NR  NR | Mbage et al. (2020) |
| **H_2_O_2_** | PW_12_/GO/FF | a | no | TMB | 652 | 3 | 1-75 μM | 0.11 μM | 10 min | 10 batches | Ma et al. (2015) |
| **NH_3_** | SiW_12_/L-cys | a | no |  | 500, 750 | > 5.2 | NR | NR | immediately | NR | Shen et al. (2012) |
| **Hg^2+^** | POM^*^ | d | industrial sewage | methanol | 427, 539 |  | 0.2-1.4 μM | 0.05 μM | 20 sec | NR | Chen et al. (2015b) |
| **cancer cells** | PV_3_Mo_9_/FA | a | 3 types of cancer cells | TMB | 652 | 7 | NR | NR | 4 h+1 min | NR | Ji et al. (2015b) |
| **UV light** | PW_12_/SPS/PP | a |  | gly, ethanol | 736 |  | NR | NR | NR | NR | Lawrie et al. (2015) |
|  | PMo_12_/LA | a |  |  |  |  | NR | NR | NR | 8 weeks | Zou et al. (2018) |
| **Dopamine and Ractopamine** | SiW_9_Co_3_ | a | no  no | H_2_O_2_  H_2_O_2_ | 475 515 |  | 1.08x10^-4^-5.38x10^-6^ M 1.56x10^-4^-3.73x10^-4^ M | 5.38x10^-6^ M  7.94x10^-5^ M | 15 min 25 min | NR  NR | Duan et al. (2018) |
| **Formaldehyde** | PMo_10_V_2_/PVC/NPOE | a | commercial milk |  | 329 |  | 0.6-8.5 mg L^-1^ | 0.2 mg L^-1^ | NR | 8 days | Veríssimo et al. (2020a) |
| **Dimethoate** | PW_12_/Myr | a | lake water and juice |  | 450 |  | NR  NR | 0.9 ng/mL | NR | NR  NR | Qi et al. (2020) |
| **ZnCl_2_.2H_2_O** | imi-SiMo_12_ | a | no |  | 635, 747 |  | 0.1-1.3 μM | 0.15 μM | NR | NR | Sabarinathan et al. (2021) |
| **Glutathione** | Mo-based POM/CR | - | mice |  | 700, 866 | 7.4 | Up to 14mM | 0.51 mM |  | 48 h | Tang et al. (2019) |

Abbreviations as reported by the authors. **CR**: croconaine; **FA**: folate acid; **FF**: diphenylalanine; **Gly**: glycerol; **GO**: graphene oxide; **GOx**: glucose oxidase; **imi**: imidazole; **L-cys**: L-cysteine; **LA**: lactic acid; **Myr**: myristoylcholine; **NPOE**: 2-nitrophenyl octyl ether; **NR**: not reported; **PDDA**: polydiallyldimethylammonium chloride; **PP:** polypropylene film; **PVC**: polyvinyl chloride; **rGO**: reduced graphene oxide; **SPS:** sulphonated polystyrene; **TMB**: 3,3’,5,5’-tetramethylbenzidine; **Trz**: 1,2,4-triazole.

^*^(n-Bu_4_N)_2_[Mo_5_NaO_13_(OCH_3_)_4_(NO)]

**POM archetype** structure according to the legend of Figure 2: a) Keggin, d) Lindqvist and -) unspecified type.

Table S8 - Lanthanides POM-based fluorescence optical sensors.

| **Targets** | **POM or POM**  **hybrid material** | **POM**  **archetype** | **Matrix** | **λex (nm)** | **λem (nm)** | **Linear range** | | **Detection limit** | | **Operation mode** | | **References** |
| --- | --- | --- | --- | --- | --- | --- | --- | --- | --- | --- | --- | --- |
| **Zn^2+^ and UV light** | EuW_10_/PyC_10_C_12_N | d |  | 260 | 590, 614 |  | | NR | | luminescent logic gate with dual output | | Zhang et al. (2006) |
| **HCl and NH_3_** | EuW_10_/agarose | d |  |  | 590, 594,  614, 621 |  | | NR | | luminescence sharply decreases with HCl gas and recover upon subsequently exposing the films to NH_3_ gas | | Wang et al. (2010) |
|  | TbW_10_/agarose | d |  | 254 | 547 |  | | 0.2731 mM | | luminescence sharply decreases with HCl gas and recover upon subsequently exposing the films to NH_3_ gas | | Wang et al. (2019a) |
| **Metanil Yellow,  Allura red, Auramine O,  Orange II** | PrW_10_/CNO | d |  | 360  360  360  360 | 410  411  410  410 |  | | 3.83 nmol mL^-1^  2.90 nmol mL^-1^  4.73 nmol mL^-1^  4.14 nmol mL^-1^ | |  | | Dutta and Sarkar, (2016) |
| **solar UV-light** | EuW_10_/PVP/PEI/AV^2+^ | d |  | 254 | 600, 630,  660, 705 | |  | | ÑR | | portable solar UV-light sensor | Liu et al. (2017) |
| **Fe^3+^ and amino-acids** | EuW_10_/UiO-67 | d |  | 336 | 471 |  | | 37 μM | | luminescence intensity quenched by Fe^3+^  (K_sv_ 2667 M^-1^) and enhanced by amino-acids | | Salomon et al. (2018) |
| **MnO_4_^-^ and**  **Cr^3+^** | EuW_10_/[C_14_-2-C_14_im]Br_2_, | d |  |  |  | 0-10 μM   0-5 mM | | 1.70 μM and  0.926 mM | | off-luminescence chemical sensor | | Sun et al. (2019) |
| **Ascorbic acid and NO_2_^-^** | EuSiMoW_10_ | a | urine, spinach |  |  | 0.1-0.9 mM and  0.05-0.4 mM | | 0.53 μM (UV-Vis) and 4.67 μM (fluorescence);  1.16 mM (UV-Vis) and 5.39 mM (fluorescence) | | reversible change of colour and luminescence | | Fu et al. (2019) |
| **Cu^2+^** | EuMnMo_6_/PPCT | - |  | 333 | 616 | 30-300 μmol mL^-1^ | | 24 nM | |  | | Yuan et al. (2019) |
| **Cr^3+^ and Ca^2+^** | EuPW_11_/PHBA | c |  | 330  330 | 619  619 | 0-10x10^-6^ M and 1.0x10^-2^-10.0x10^-2^ M | | 1.423 mM and 0.676 mM | | luminescence intensity quenched by Cr^3+^, and enhanced by Ca^2+^ | | Wu et al. (2019) |
| **Vitamin C and H_2_O_2_** | TbP_2_Mo_18_ | b |  | 377 | 547 |  | | NR | |  | | Bin et al. (2019) |
| **Ba^2+^** | Eu-arsenotungstates/ H_2_tpdc | - |  |  | 615 |  | | 1.19x10^-3^ mM | | good recognition responses toward detecting the Ba^2+^ ion in the absence of Ca^2+^ or Sr^2+^ ions in aqueous system | | Wang et al. (2020d) |
| **Cu^2+^ and**  **L-cysteine** | EuSe_3_W_14_^*^ | b |  | 394 | 614 | 1.00x10^-2^-2.50x10^-1^ mM and 0.02-0.14 mM | | 1.24x10^-3^ mM and 2.17x10^-4^ mM | | turn-off /on, K_SV_ 5.92 mM^-1^ | | Zhang et al (2020a) |
|  | EuTeW_9_^**^ | - |  | 394 | 614 | 1.0x10^-5^-8.0x10^-2^ mM and  0.02- 0.14 mM | | 8.82x10^-6^ mM and 1.75x10^-4^ mM | | turn-off/on, K_SV_ 123.17 mM^-1^ | | Zhang et al. (2020b) |
| **Temperature** | EuW_10_/ Tb-TATB | d |  | 330 | 530-560,  602-633 |  | | NR | |  | | Viravaux et al. (2021) |
| **Ag^+^ and**  **cholyglycine** | Eu_4_W_8_/EB-TFP | - | tap and river water | 316  284 | 615 615 | 10^-8^-10^-3^ M and 10^-8^-10^-3^ M | | 0.014 μg mL^-1^ and 0.024 μg mL^-1^ | | luminescence turn-on/off | | Wang et al. (2021) |

Abbreviations as reported by the authors. **AV^2+^**: N,N´-bis(δ-aminopropyl)-4,4´-bipyridine bromide hydrobromide; **CNO**: carbon nano-onion; **EB**: ethidium bromide; **NR**: not reported; **PEI**: Polyethyleneimine; **PHBA**: p-hydroxybenzoic acid; **PPCT**: 4' 2,2':6',2"para-phenylcarboxyl-terpyridine; **PVP**: polyvinylpyrrolidone; **PyC_10_C_12_N**: trans-10-(4-(49-pyridylvinylene)-phenyl)oxydecyldodecyldimethylammonium bromide; **TATB**: triazine-1,3,5-tribenzoic acid; **TFP**: 1,3,5-triformylphloroglucinol; **tpdc**: 2,5- thiophenedicarboxylic acid; **UiO-67**: zirconium luminescent metal-organic framework.

^*^[H_2_N(CH_3_)2]_10_H_3_{SeO_4_Eu_5_(H_2_O)_8_[Se_2_W_14_O_52_]_2_}·40H_2_O

^**^K_14_H_10_[Eu_4_(H_2_O)_4_W_6_(H_2_glu)_4_O_12_(B-α-TeW_9_O_33_)_4_]·60H_2_O

**POM archetype** structure according to the legend of Figure 2: a) Keggin, b) Dawson, c) Anderson, d) Lindqvist and -) unspecified type.

Table S9- POM-based fluorescence optical sensors.

| **Target** | **POM or POM**  **hybrid material** | **POM archetype** | **Matrix** | **Substrates** | **λex (nm)** | **λem (nm)** | **Operation mode/Linear range/Limit of detection** | **References** |
| --- | --- | --- | --- | --- | --- | --- | --- | --- |
| **Cu^2+^ and**  **Pb^2+^** | SiW_10_/dansyl | a | no |  | 324  324 | 457  525 | Fluorescence quenched by Cu^2+^ and enhanced  by Pb^2+^ | Carraro et al. (2012) |
| **pH** | Mo_8_/norfloxacine | j | no |  | 330 | 440 | Acid–base switch | Liu et al. (2015) |
| **VOCs** | Mo_8_/[Ir^III^(ppy)_2_(bpy)]^+^ | - | no |  | 365 | 585 | Depending on VOC polarity | Bolle et al (2016) |
| **Picric acid and Pd^2+^** | V_10_O_28_/Cu-pyno-NEt | e | no |  | 320 | 384 | LOD 0.18 ppb for picric acid LOD 0.80 ppb for Pd^2+^, K_SV_ (Pd^2+^) 4.45x10^1^ M^-1^ | Raizada et al. (2017) |
| **H_2_O_2_** | SiW_9_ | a | water | BA  TH  HPPA | 295  375  330 | 405 440   416 | Linear range 10 nM – 1.6 μM, LOD 6.7x10^-9^ M Linear range 1.6 μM – 10 mM, LOD 2.2x10^-7^ M Linear range 10 μM – 0.25 mM, LOD 9.6x10^-6^ M | Tian et al. (2019) |
| **Hg^2+^** | Zn-dbt/P_2_W_18_  Cd-dbt/P_2_W_18_ Cd-dbt-Cl/PW_12_ Cd-dbt/SiW_12_ | b  b  a  a | no |  | 320  320  320  320 | 420  420  440  440 | NR | Ying et al. (2019) |
| **Hg^2+^** | Ag- Py_2_TTz /PMo_12_ | a | no |  | 365 | 422 | NR | Mou et al. (2020) |
| **Hg^2+^** | Cu-dm4bt/PMo_12_ | a | no |  | 365 | 460 | NR | Wang et al. (2020b) |
| **Hg^2+^** | Zn-MET/CrMo_6_  Cu-2,2'- bpy/CrMo_6_ | j  j | no |  | 310  310 | 360  360 | For both POM-composites the fluorescence is quenched to a large extent by Hg^2+^ | Zhang et al. (2021a) |
| **Dopamine** | FeMo_6_/rGO | c | Human serum and dopamine hydrochloride injection | OPD | 433 | 548 | Linear range 1-80 μM, LOD 0.0112 μM  Fast response time (4 min) | Li et al. (2021) |

Abbreviations as reported by the authors. **BA**: benzoic acid; **dm4bt**: 2,2’-dimethyl-4,4’-bithiazole; **HPPA**: 3-(4-hydroxyphenyl) propionic acid; **MET**: 4-(3-imidazol-1-yl-ethyl)-4H-[1,3,4]triazole; **NEt**: Triethylamine; **NR**: not reported; **OPD**: *o*-phenylenediamine, **PPy**: polypyrrole; **Py2TTz**: 2,5-bis(4-pyridyl)thiazolo[5,4-*d*]thiazole; **Pyno**: 4-picoline N-oxide; **rGO:** reduced graphene oxide; **TH**: thiamine.

**POM archetype** structure according to the legend of Figure 2: a) Keggin, b) Dawson, c) Anderson, e) decavanadate, j) γ-octamolybdate, and -) unspecified type.
